# Comparison and Analysis of Timbre Fusion for Chinese and Western Musical Instruments

**DOI:** 10.3389/fpsyg.2022.878581

**Published:** 2022-07-07

**Authors:** Jingyu Liu, Shuang Wang, Yanyin Xiang, Jian Jiang, Yujian Jiang, Jing Lan

**Affiliations:** ^1^State Key Laboratory of Media Convergence and Communication, Communication University of China, Beijing, China; ^2^Key Laboratory of Acoustic Visual Technology and Intelligent Control System, Ministry of Culture and Tourism, Communication University of China, Beijing, China; ^3^Beijing Key Laboratory of Modern Entertainment Technology, Communication University of China, Beijing, China; ^4^School of Information and Communication Engineering, Communication University of China, Beijing, China; ^5^China Digital Culture Group Co., Ltd, Beijing, China; ^6^Center for Ethnic and Folk Literature and Art Development, Ministry of Culture and Tourism, Beijing, China

**Keywords:** timbre, fusion, auditory perception, acoustic parameters, Chinese and Western instruments, instrument acoustics, cross-cultural

## Abstract

Timbre fusion is the theoretical basis of instrument acoustics and Chinese and Western orchestral acoustics. Currently, studies on timbre fusion are mainly focused on Western instruments, but there are some studies on the timbre fusion of Chinese instruments. In this paper, the characteristics of timbre fusion for Chinese and Western instruments are explored, focusing on the subjective attributes and objective acoustic parameters, and a series of experiments is carried out. First, a database containing 518 mixed timbre stimuli of Chinese and Western instruments was constructed to provide basic data that are necessary for the subjective and objective analyses of timbre fusion. We designed and conducted a subjective evaluation experiment of timbre perception attributes based on the method of successive categories. The experimental data were processed using statistical approaches, such as variance analysis, multidimensional preference analysis, and correlation analysis, and we studied the influence of the temporal envelopes and instrument types on fusion, segregation, roughness, and pleasantness. In addition, the differences between Chinese and Western instruments were compared based on these four perception attributes. The results show that fusion and segregation are the most important attributes for Chinese instrument timbre, while roughness is the most important attribute for Western instrument timbre. In addition, multiple linear regression, random forest, and multilayer perceptron were used to construct a set of timbre fusion models for Chinese and Western instruments. The results show that these models can better predict the timbre fusion attributes. It was also found that there are some differences between the timbre fusion models for Chinese and Western instruments, which is consistent with the analysis results of subjective experimental data. The contribution of acoustic objective parameters to the fusion model is also discussed.

## Introduction

### Background

Since the twentieth century, Western music culture has been gradually introduced in China. Western symphony orchestras, with their rich instrument timbre, powerful expressive force, and standardized orchestral arrangements and sound effects, have led to new inspirations and musical forms of Chinese folk music. The “fusion” of all musical instruments is the basic aesthetic principle of Western symphony. In the long-term development of symphonic creation, both harmony and orchestration have formed relatively mature acoustic theory and have been relatively successful with respect to practical experience. “Fusion” refers to a relationship that occurs after the combination of timbre, that is, the combined effect produced by the simultaneous sound of different instruments. From acoustic theory, fusion can be understood as the degree of harmonic integration of musical instruments (Li, 2020). Most instruments in Western symphony orchestras have a high degree of consonance, which means that each harmonic is, basically, an integer multiple of the fundamental frequency, making the overall sound effect is integrated and unified (Wang et al., 2016).

Instruments in Western symphony orchestras have many timbre characteristics that are not prominent, so it is easy to achieve the effect of fusion by producing compound timbre in orchestration, which refers to the timbre composed of two musical instruments whose timbre is very similar (Li, 2020). When these two instruments play the same or octave melody at the same time, it is difficult to distinguish them. For example, the sound of a violin and viola can be considered a “compound timbre.” Here, “compound timbre” corresponds to “timbral emergence,” which was proposed by Sandell (1995); that is, all sounds are blended and unidentifiable (McAdams, 2019). Due to the differences between Chinese and Western cultural backgrounds, symphony orchestras and Chinese orchestras have different orchestration ideas. For a long time, musicians have explored the diversification of Chinese musical instruments' timbre in practice and philosophy, borrowed compositional techniques from traditional music, and formed their own aesthetic principles. It is difficult to produce the so-called “compound timbre” between Chinese instruments, but a “mixed timbre” can be produced between them. The so-called “mixed timbre,” which is both harmonious and independent and both related and separated, refers to the timbre combined and superimposed by different musical instruments (Li, 2020). Here, “mixed timbre” corresponds to “timbral heterogeneity,” which was proposed by Sandell (1995). Timbral heterogeneity is a unique timbre characteristic of Chinese orchestral music, where a beautifully mixed sound with both individuality and combination is formed. In fact, this paper states that Western instruments more easily achieve the fusion effect in orchestration, which means that the maximum fusion effect between Western instruments is better than that of Chinese instruments. The fusion mentioned in this manuscript does not refer to the composer's orchestration. In other words, for a Western orchestra, if the composer wants a fusion effect, he or she can achieve it through a combination of existing Western instruments. For a Chinese orchestra, it is difficult for the composer to find a combination of two or more instruments to achieve the effect of fusion. We have previously performed experiments on the timbre contrast between Chinese instruments and Western instruments and found that the timbre of Chinese instruments is, overall, rougher than that of Western instruments. Moreover, the distribution of Chinese instruments is more dispersed in the three-dimensional timbre space, and the timbre similarity is lower (Jiang et al., 2020).

Currently, the orchestration theory and music practice of Chinese orchestras are still in the exploration stage, and the problem of the fusion between instruments in Chinese orchestra still exists. The Chinese orchestra is composed of folk instruments, most of which have evolved from ancient Chinese instruments and have a distinctive timbre. Therefore, the sound of the Chinese orchestra as a whole has the auditory feeling of “sharp, dry, messy, and noisy.” In addition, there are some problems in the Chinese orchestra, such as volume imbalance of the vocal part, low degree of integration between timbres, and uncertain composition of the orchestra. Therefore, on the basis of the aesthetic principles of Chinese music and timbre characteristics of Chinese musical instruments, we should further explore the timbre combination rules for musical instruments.

This paper takes musical instrument combinations as the research object to discuss the differences in the timbre fusion of different musical instrument combinations. A comparative analysis of the fusion of timbre combinations in Western symphony and Chinese orchestra is also presented, and references and theory for Chinese orchestra orchestration are provided. Next, the current research status is summarized from three aspects: the definition of fusion, perception experiments of fusion, and subjective and objective parameters that affect fusion.

### Definition of Fusion

Currently, there is more than one definition of timbre fusion within the academic circle. For example, McAdams (2019) proposed that the result of combining sounds concurrently in orchestration is a timbral blend, when events fuse together, or timbral heterogeneity, when they remain separate. Concurrent grouping determines how components of sounds are grouped together into musical events, a process referred to in psychology as auditory fusion. In describing sound quality as a whole, the sense of fusion is one of the important attributes that are used to express the degree of acoustic integration between the whole band or chorus, solo instruments or collaborative instruments, and singing or accompaniment.

The concept of fusion may have been first proposed by Stumpf (DeWitt and Crowder, 1987), who proposed the principle of tonal fusion, defining the fusion as the degree to which two simultaneous monophonic tones are perceived acoustically as one sound. He believed that fusion was the basis of tonal consonance (Apel, 2003). Subsequently, DeWitt and Crowder (1987) further developed Stumpf's theory. They performed three experiments and investigated Stumpf's fusion principle of tonal consonance. The results of this experiment showed that fusion may represent the tendency for people to interpret pitch combinations that could represent harmonics, resulting from a single fundamental as timbres rather than as chords.

Timbre emerges from the perceptual fusion of acoustic components into a single auditory event, including the blending of sounds produced by separate instruments in which the illusion of a “virtual” sound source is created (McAdams, 2019). Bregman and Pinker demonstrated the interplay of concurrent fusion and sequential stream formation and conceived a sort of competition between the two auditory organization processes. Therefore, the attack asynchrony and the decomposition of simultaneities into separate auditory streams, whose events are timbrally similar, work together to reduce the degree of perceptual fusion (Bregman and Pinker, 1978). Timbre is a property of fused auditory events.

### Perceptual Experiments on Fusion

Concerning the term “fusion” and its different interpretations, we structured perceptual experiments on fusion into (1) its involvement in concurrent groupings, as in spectral fusion, which forms a timbral identity and (2) the special case of instrument combinations, where it has commonly been referred to as the timbral blend.

McAdams proposed the concept of spectral fusion (McAdams, 1982), which belongs to the first category. An important perceptual aspect of the formation of auditory images evoked by acoustic phenomena is the distinguishing of different sound sources. To form images of sound sources, the auditory system must be able to perceptually fuse the concurrent elements that come from the same source and separate the elements that come from different sources. Then, the relationship between spectral fusion auditory sensory cues was further studied (McAdams, 1984). The results showed that the acoustic cues that contribute to the formation and distinction of multiple, simultaneous source images that are investigated include the harmonicity of the frequency content, the coherence or low-frequency frequency modulation, and the stability and/or recognizability of spectral form when coupled with frequency modulation. Shields and Roger (2004) studied the relationship of timbre to dissonance and spectral fusion. In this experiment, listeners rated dissonance and blend levels for a set of dyads involving fourteen interval sizes and twenty-five orchestral combinations. The researchers related dissonance and spectral fusion to the timbre of time-variant steady-state dyads. The experimental results show that interval size and orchestration are significantly influenced by both dissonance and blend ratings.

At approximately the same time, Carterette and Kendall (1989) and Kendall and Carterette (1991) also conducted similar experiments on timbre. Sandell (1989a,b) reported preliminary work on the “blend” of “concurrent timbres” using 15 of Grey's (1975) line-segment approximations of brief real instrument tones. The results of interest demonstrated that a blend is related to the summed distribution of energy in the harmonic series of the two tones, with a less blend correlated with more energy in higher harmonics compared to lower harmonics. Sandell's (1991) doctoral thesis provided a detailed overview of the concept of fusion. This study investigated the acoustical correlates of a blend for 15 natural-sounding orchestral instruments presented in concurrently sounding pairs. Sandell's acoustically based guidelines for a blend, which augment instance-based methods of traditional orchestration teaching, provided underlying abstractions that are helpful for evaluating the blend of arbitrary combinations of instruments. Sandell (1995) also proposed three possible perceptual results of instrument combinations: timbral heterogeneity, timbral augmentation, and timbral emergence. Kendall and Carterette (1993) reported on a series of experiments directed toward questions concerning the timbres of simultaneous orchestral wind instruments. In this study, researchers ascertained the degree of a blend and identifiability of soprano orchestral winds. It was found that the degree of a blend corresponded with the positions of instruments in a two-dimensional similarity space.

### Subjective and Objective Parameters Affecting Fusion

Regarding the subjective perception attributes describing the fusion of timbre, different studies have provided representative terms from different perspectives. In the experiment of Bregman and Pinker (1978), compound sounds composed of two pure tones with different frequencies were used as experimental stimuli. Compound sounds are somewhat dissonant and are described as “rough” or “complex.” DeWitt and Crowder (1987) further supplemented Stumpf's theory and proposed three pairs of evaluation terms to describe musical intervals: consonance-dissonance, smoothness-roughness, and pleasant-unpleasant. Kim (2018) investigated how musicians perceive and compensate for the interacting effects of timbre, blend and sensory dissonance when tuning and rating harmonic intervals. In this experiment, timbre terminology, such as rough, unpleasant, smooth, and pleasant, was used to describe the timbre perception properties of the trumpet and vibraphone. Sounds that differ acoustically are organized by the auditory system into separate percepts called auditory streams (Bregman and Campbell, 1971). A physical sound source can produce a sequence of successive acoustic events. To examine this phenomenon, Fischer et al. (2021) used naturalistic orchestral excerpts from the symphonic repertoire to examine perceptual segregation.

Beating is an important factor causing roughness. In this experiment, dyads are in pitch unison or octave. These dyads thus exhibit a very low degree of roughness. In our previous pre-experiment, we found a certain negative correlation between roughness and a degree of fusion. Previous research studies have, indeed, shown that dyads in pitch unison are perceived to be more blended than dyads involving non-unison pitches (Kendall and Carterette, 1993; Jingyu, 2013; Lembke et al., 2019). Combining all of these studies, we have chosen four timbre perception attributes, fusion, roughness, segregation, and pleasantness for subjective evaluation experiments.

Researchers have also explored the relationship between fusion and objective acoustic parameters. Fusion is affected by sensory cues, such as whether the acoustic components begin synchronously, whether they are related by a common period, and whether there is coherent frequency and amplitude behavior (McAdams, 1984). The coherent behavior cues are related to the Gestalt principle of common fate. In other words, sounds that change in a similar manner are likely to have originated from the same source (Bregman, 1994).

The degree of fusion also depends on spectrotemporal relations among the concurrent sounds (Siedenburg et al., 2019). Sandell (1995) demonstrated that sounds blend better when they have similar attack envelopes and spectral centroids, as well as when their composite spectral centroid is lower. This experiment also found that the more similar these parameters are for the two combined sounds, the greater their blend. Tardieu and McAdams (2012) performed two experiments on combinations of pitched impulsive and sustained sounds. They highlighted the audio descriptors, underlying the perception of a blend and the perception of emergent timbre for dyads composed of one impulsive and one sustained sound. In both experiments, the attack time was very important, as it was one of the two most important factors in predicting both a blend and emergent timbre perception. Chon and Huron (2014) proposed the concept of timbre salience. In this paper, they examined the identification of an instrument sound in concurrent unison dyads. As a salient timbre is defined as one that captures listeners' attention easily and tends not to blend well with concurrent sounds (Chon and McAdams, 2012), we can logically expect that a salient timbre will be easily identified.

In addition to the acoustic parameters mentioned above, researchers have proposed other features that describe the fusion of timbre. Rossetti (2016) discussed timbre and sound morphology in live electroacoustic and instrumental music from a compositional standpoint and convergence issues in live electroacoustic music. They proposed that timbre fusion should be addressed based on the concepts of jitter, permeability, and timbre of movement. In addition to the study of timbre fusion for Western symphonies, some researchers have studied the timbre characteristics of African music (Fales and McAdams, 1994). The authors presented the results of perceptual and acoustic investigations of the fusion and “layering” of noise and tone. The results also exemplified the fusion of two extremely different timbres with implications for the blending of instrumental timbres in an orchestral setting.

In addition to global descriptors, such as the spectral centroid, research has been conducted on the role of local descriptors of formant structure (Siedenburg et al., 2019). Goodwin (1980) studied the acoustic parameters of individual voices in choral blends. The phenomenon of a choral blend was investigated by identifying spectral differences between vocal sounds produced in solo singing and in unison ensemble singing to achieve the optimum blend. Reuter (2003) studied the relationship between stream segregation and formant areas. The results are as follows: Alternating timbres with equivalent main formant areas tend to produce one sole, continuous melody in perception. Alternating timbres with non-matching formant areas tend to produce two distinct melodies in perception. Lembke and McAdams (2012, 2015) investigated the acoustical and perceptual factors involved in timbre blending between orchestral wind instruments based on a pitch-invariant acoustical description of wind instruments. A possible perceptual relevance for these formants was tested in their experiments, employing different behavioral tasks. The results showed that the relative frequency location and magnitude differences between formants can be shown to bear a pitch-invariant perceptual relevance to blend for several instruments.

In the context of perceptual blending between orchestral timbres, holistic acoustical descriptions of instrument-specific traits can assist in the selection of suitable instrument combinations (Lembke et al., 2013). Researchers have proposed several parameters, such as spectral maxima or formants, which have been shown to influence timbre blending involving frequency relationships between local spectral features, their prominence as formants, and constraints imposed by the human auditory system. Computational approaches to predict a timbre blend have been proposed that are based on these factors and explain ~85% of the variance in behavioral timbre-blend data.

In summary, research on timbre fusion has mostly focused on Western instruments, and there is, currently, no study on Chinese instruments. To explore the rules of timbre fusion for Chinese instrument combinations and compare the differences between Chinese and Western instruments, a dataset has been constructed for this study that contains a combination of Chinese and Western instruments. Through the statistical processing of experimental data, the differences between Chinese and Western musical instruments in timbre fusion are analyzed, and the subjective and objective acoustic parameters affecting timbre fusion are also analyzed. In addition, a timbre fusion model for Chinese and Western instruments, which provides basic theory and data support for the orchestration of Chinese and Western instruments, is constructed for this study.

The following sections of this paper are arranged as follows. In Section Methods, the second part, the four-part method, which includes the participants, stimuli, apparatus and procedure, is introduced. In Section Subjective Evaluation Experiment and Data Analysis, the statistical analysis of the experimental data, including the factors affecting the fusion and the comparison of the timbre fusion between Chinese and Western instruments, is presented. In Section Construction of the Timbre Fusion Model, the construction of the timbre fusion model is described. Multiple linear regression, random forest, and multilayer perceptron methods were used to construct the fusion model of Chinese instruments and Western instruments. In Section Discussion, the discussion and summary are presented.

## Methods

### Participants

Thirty-two participants, including 15 males and 17 females (between 18 and 35 years of age), took part in this test. All the participants had received routine listening training for more than 1 year (M = 1.62, SD = 0.38). All the participants listened to different types of Chinese music, such as Jiangnan Sizhu, Fujian Nanyin, Guangdong music, and Chinese orchestral symphony, and Western music, such as pop, rock, classical, blues, and R&B. Among the participants, 22 of them listened to Chinese and Western music in a concert hall. All the participants met the required hearing threshold of 20 dB HL by a pure-tone audiometric test with octave-spaced frequencies from 125 to 8 kHz (Martin and Champlin, 2000). The participants, who were university students raised in China, were recruited in Beijing. The participants signed an informed consent form and were compensated for their participation.

### Stimuli

**Figure 1:**
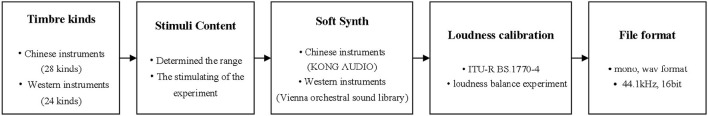
Production of single-tone stimuli.

**Figure 2:**
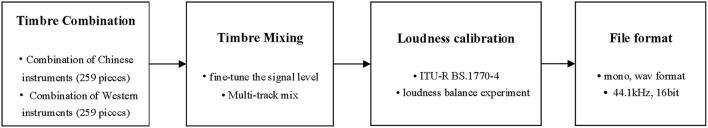
Production of mixed timbre experimental stimuli.

There were 518 mixed timbre (composed of two timbre) stimuli. The process of making mixed timbre stimuli consisted of two steps. The first step was to determine the types of single-tone instruments to be mixed. Then, single-tone stimuli were created. The second step was to combine the single-tone stimuli to form some mixed stimuli based on two tones. The following is a detailed description of the stimuli production process, which is shown in Figures 1, 2.

#### Production of Single-Tone Stimuli

Fifty-two kinds of instrument timbres were selected in this experiment. There were 24 Western instrument timbres, including wood wind instruments, brass wind instruments, bowed string instruments, hammered string instruments, and percussion instruments, and 28 Chinese instrument timbres, including wind instruments, bowed string instruments, plucked instruments, and percussion instruments. The experiment stimuli comprised four phrases in the lyric paragraph of the second part of the Spring Festival prelude. The music score is shown in Figure 1 of the Appendix. The range of each instrument is used most often by composers. The experimental stimuli were made by the combination of MIDI and a sampling sound source. Among them, the stimuli of Western instruments were produced by the Vienna Symphonic Library,^1^ and the stimuli of Chinese instruments were produced by the Kong Audio Sound Library^2^. The audio file format was saved in the WAV format, the sampling frequency was 44.1 kHz, and the quantization accuracy was 16 bits. The timbre types of the Chinese and Western instruments and the specific range of each instrument are shown in Table 1 of the Appendix.

According to the definition of timbre, it is necessary to exclude the influence of pitch and loudness when studying it. Previous studies have shown that, in some cases, timbre and tone are inseparable (Melara and Marks, 1990). Therefore, the timbre perception features extracted in this paper also included the pitch factor. To avoid the influence of loudness on the perception results, all stimuli were first calibrated based on a loudness measurement algorithm (ITU-RBS 1770-4, 2015). Then, three audio engineers with music backgrounds fine-tuned the signal level based on the results of the music loudness balance experiment. The specific process of the loudness balance experiment and the statistical analysis of the experimental results have been detailed in previous research results (Zhu et al., 2018).

#### Production of Mixed Timbre Experimental Stimuli

Twenty-eight single-tone stimuli of Chinese instruments were divided into four groups: wind instruments, bowed string instruments, plucked instruments, and percussion instruments. By combining these stimuli in pairs within and between groups, we obtained 259 mixed timbre stimuli of Chinese instruments. Twenty-four single-tone stimuli of Western instruments were divided into five groups: wood wind instruments, brass wind instruments, bowed string instruments, hammered string instruments, and percussion instruments. By combining these stimuli in pairs within and between groups, we obtained 259 mixed timbre stimuli of Western instruments. In fact, the 24 Western musical instruments and 28 Chinese musical instruments each have more than 259 kinds of combinations. Considering the amount of experimental data, we only evaluated the common timbre combination methods in the orchestration. These dyads are often used by composers. The combination of these dyads references the Chinese National Orchestra Practical Orchestration Manual. Similar to the single-tone stimuli, to avoid the influence of loudness on the perception results, all mixed experimental stimuli were first calibrated based on a loudness measurement algorithm (ITU-RBS 1770-4, 2015). Finally, a collection of 518 mixed stimuli was obtained, as shown in Table 2 of the Appendix.

### Apparatus

The experiment was carried out in a listening room, conforming to standards (EBU-TECH 3253, 2008). The reverberation time of the listening room was 0.3 s, the sound field distribution was uniform, and there was no bad acoustic phenomenon or body noise. A Genelec 1038B three-way active midfield monitoring speaker was used to replay the experimental signals. Its parameters, which conform to international standards, are shown in Table 3 of the Appendix.

Because the experimental results are affected by the listening sound pressure level, it is necessary to ensure that the participants listen at the standard level (EBU-TECH 3253, 2008), and that this level remains unchanged throughout the experiment. The equipment used in the calibration test system is a Lenovo T460 notebook computer, a BK4231 sound calibrator, a BK2250 sound-level meter, and a YAMAHA 01V96i digital mixer. The actual listening pressure level is 74 dBA, which conforms to the international listening standard (EBU-TECH 3253, 2008). Experimental stimuli were played using Adobe Audition software. The seats in the listening room were arranged in triangles. That is, in the listening area, one listener sat in the first row, two listeners sat in the second row, and so on. To avoid presentation level changes caused by the shielding of the front seats from the back seats, the back seats were all 15 cm higher than the front seats. In the process of listening, the ear height of the participants should be at the same level as the midpoint of the vertical line in the high and low sounds of the speakers. We calibrated the test system with the sound-level meter. After the system was calibrated, white noise was used as the test signal. The sound-level meter was located in the center of the listening seat triangle. The system volume was adjusted so that the A-meter sound level of the system was 74 dBA (as read from the sound-level meter).

### Procedure

**Figure 3:**
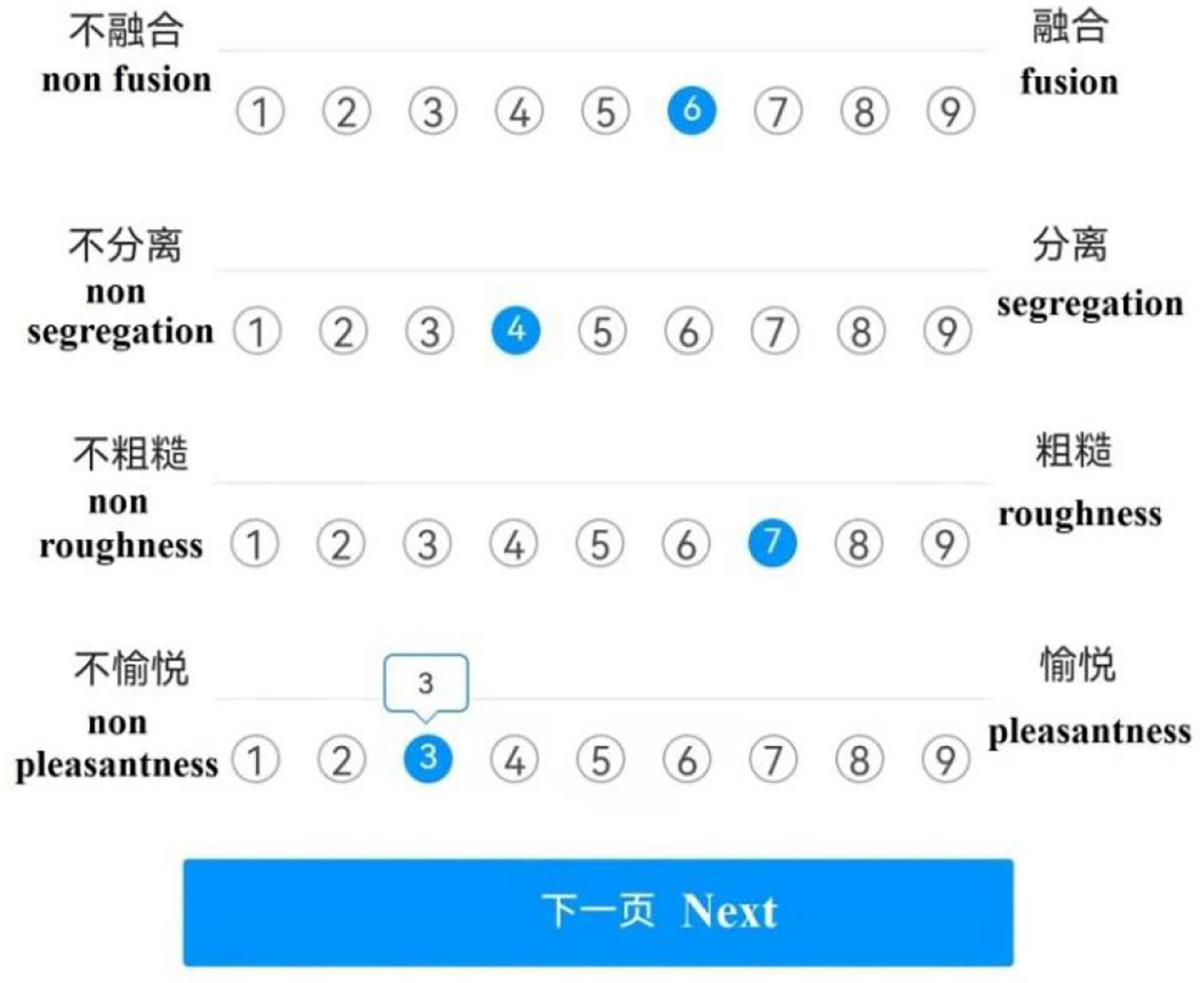
The interface for the listening test.

The experimental steps included four stages: the experimental introduction stage, the pre-experimental stage, the training stage, and the formal experimental stage. The experimental introduction stage: The background of the experiment was introduced, and the participants were informed of the purpose of the experiment to enhance their cognition. Then, we explained the concept of the timbre perception attribute to the participants and used the example audio stimuli as an aid so that the participants could accurately understand the meaning of each attribute. The pre-experimental stage: We explained the corresponding relationship between the value of the 9-level evaluation scale and the degree of integration to the participants. Then, we randomly played all audio stimuli to familiarize the participants with their variation range. The training stage: Three timbre stimuli were randomly selected. The participants were asked to evaluate the fusion, segregation, roughness, and pleasantness of the stimuli according to their subjective feelings using the 9-level evaluation scale (1–9). The purpose of this step was to familiarize the participants with the experimental process and to avoid any experiment impacts related to unfamiliarity with the experimental process in the formal experimental stage. These data were not used for the analysis of the final results. The formal experiment stage: Thirty-two subjects were randomly divided into four groups. Each group consisted of eight subjects. A total of 518 stimuli were randomly divided into 52 stimuli groups. The order within a stimuli group was fixed, which was generated by a random program. And the order of the groups was random. To avoid the possibility of participant fatigue from listening to the sounds for a long amount of time, all experimental stimuli were divided into three sets. The experimental time of each set was no more than 30 min, and the rest, between each set, was 15 min. Each participant used a smartphone app to provide his or her responses. Then, we played the stimuli groups for each subject group. The participants were not allowed to communicate with one another during the test. The participants evaluated the fusion, segregation, roughness, and pleasantness of the timbre stimuli they heard and filled out the forms accordingly. The experimental data were collected by the app, in which we can select scores and export data. The app interface is shown in the figure below (Figure 3). The experiment was carried out according to the above steps, and the data collection was completed. The above steps were followed to conduct subjective evaluation experiments and complete the data collection.

## Subjective Evaluation Experiment and Data Analysis

The steps for the subjective evaluation of the experimental data analysis are as follows. First, the original experimental data were tested for reliability and validity, in which the reliability test was conducted by calculating Cronbach's alpha, and the validity test was conducted by calculating the standard deviation. Then, the method of successive categories was used to statistically analyze the experimental data, and the psychological scales of all samples in the four dimensions of fusion, segregation, roughness and pleasantness were obtained. These data were used for the comparative analysis of the timbre fusion of Chinese and Western instruments and the construction of timbre fusion modeling. Third, analysis of variance was used to statistically analyze the fusion, segregation, roughness, and pleasantness, and the differences in the four-dimensional attributes of different musical instrument timbre combinations were obtained. Finally, correlation analysis and multidimensional preference analysis were used to explore the relationship between the timbre perception dimension, musical instrument types, and temporal envelopes, and conclusions from the analyses were given.

### Reliability and Validity Tests

Cronbach's alpha is used to evaluate the internal consistency of questionnaires and is applicable to the reliability analysis of attitudes, questionnaires or scales. Cronbach's alpha value is between 0 and 1. The higher the alpha coefficient is, the higher the reliability and the better the internal consistency of the questionnaire. Generally, a questionnaire with an alpha coefficient above 0.8 has value that is useful, and a questionnaire with an alpha coefficient above 0.9 shows that the reliability of the questionnaire is very good. The calculated Cronbach's alpha values of the four timbre perception attributes are shown in Table 4 of the Appendix. As seen from this table, the Cronbach alpha values were 0.932 for fusion, 0.941 for segregation, 0.926 for roughness, and 0.918 for pleasantness. These measures indicated that all scales had very good internal consistency among the 32 participants.

The validity test was designed to examine the validity of the experimental results. The higher the validity is, the better the measure shows the characteristics it is intended to measure. Different experiments have different purposes and require different levels of validity. The validity test for this experiment was to calculate the standard deviation of the experimental data for the 32 subjects for each experimental stimulus and to consider the experimental data beyond 1.5 times the standard deviation to be invalid and to eliminate them. Since the statistical model of the method of successive categories to be used next requires that there be no missing values in the experimental data, the data within 1.5 times the standard deviation of each stimulus were averaged, and the mean was used to fill in the missing values that were eliminated. After the reliability and validity tests, the data were statistically analyzed using the method of successive categories.

### Data Statistics Based on the Method of Successive Categories

The experimental data were counted by the method of successive categories (Zihou, 2008). The theoretical basis of this method is to assume that the psychological quantity is a random variable that is subject to a positive Pacific distribution, and the boundary of each category in the method of successive categories is not a predetermined value but a random variable determined according to the experimental data. According to the Thurstone model, the preference of object *a*_*i*_ is the probability variable *X*_*i*_ on the preference scale, which follows the normal distribution, and its preference psychological scale *f* (*a*_*i*_) = *S*_*i*_. The dividing line between category *g* and category *g* + 1 is the random variable *T*_*g*_ on the subjective preference scale, which also follows the normal distribution $\left(T_{g},\;\sigma^{2}\right)$. *T*_*g*_ and *f* (*a*_*i*_) satisfy the following relationship. The category judgment model is shown in Table 1.

**Table 1:** The category judgment model.

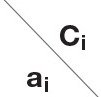	**C_1_**	**C_2_**	**…**	**C_m−1_**
*a*_1_	*t*_1_ − *f*(*a*_1_) = *z*_11_	*t*_2_ − *f*(*a*_1_) = *z*_21_	…	*t*_*m* − 1_ − *f*(*a*_1_) = *z*_*m*−11_
*a*_2_	*t*_1_ − *f*(*a*_2_) = *z*_1_	*t*_2_ − *f*(*a*_2_) = *z*_22_	…	*t*_*m* − 1_ − *f*(*a*_2_) = *z*_*m*−12_
*a*_*n*_	*t*_1_ − *f*(*a*_*n*_) = *z*_1*n*_	*t*_2_ − *f*(*a*_*n*_) = *z*_2*n*_	…	*t*_*m* − 1_ − *f*(*a*_*n*_) = *z*_*m* − 1*n*_
Sum	*nt*_1_ − ∑*f*(*a*_*j*_)	*nt*_2_ − ∑*f*(*a*_*j*_)	…	*nt*_*m* − 1_ − ∑*f*(*a*_*j*_)
Average	$t_{1}-\frac{1}{n}\sum f\left(a_{j}\right)$	$t_{2}-\frac{1}{n}\sum f\left(a_{j}\right)$	…	$t_{m-1}-\frac{1}{n}\sum f\left(a_{j}\right)$

**Figure 4:**
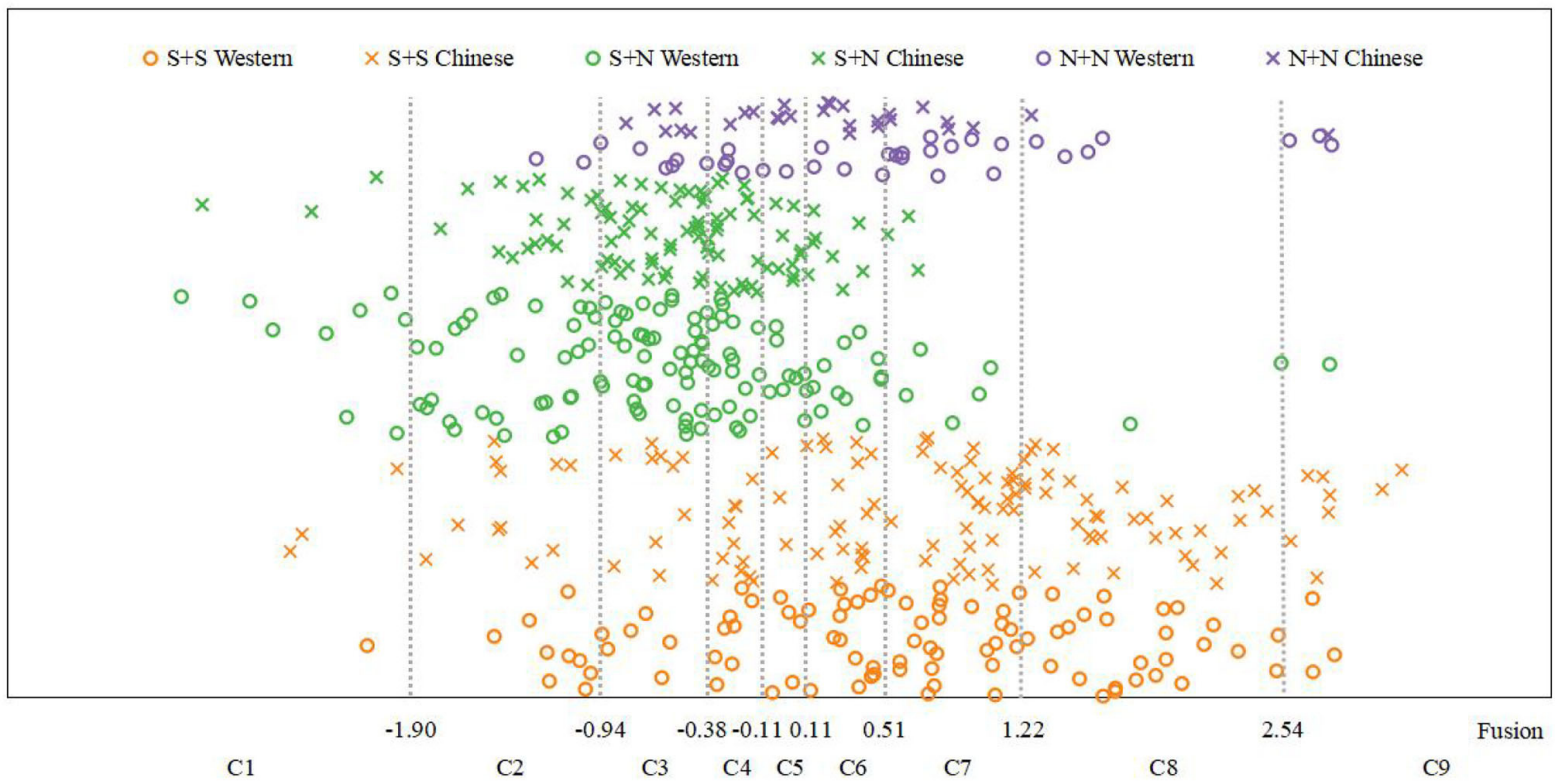
Fusion psychological scales.

**Figure 5:**
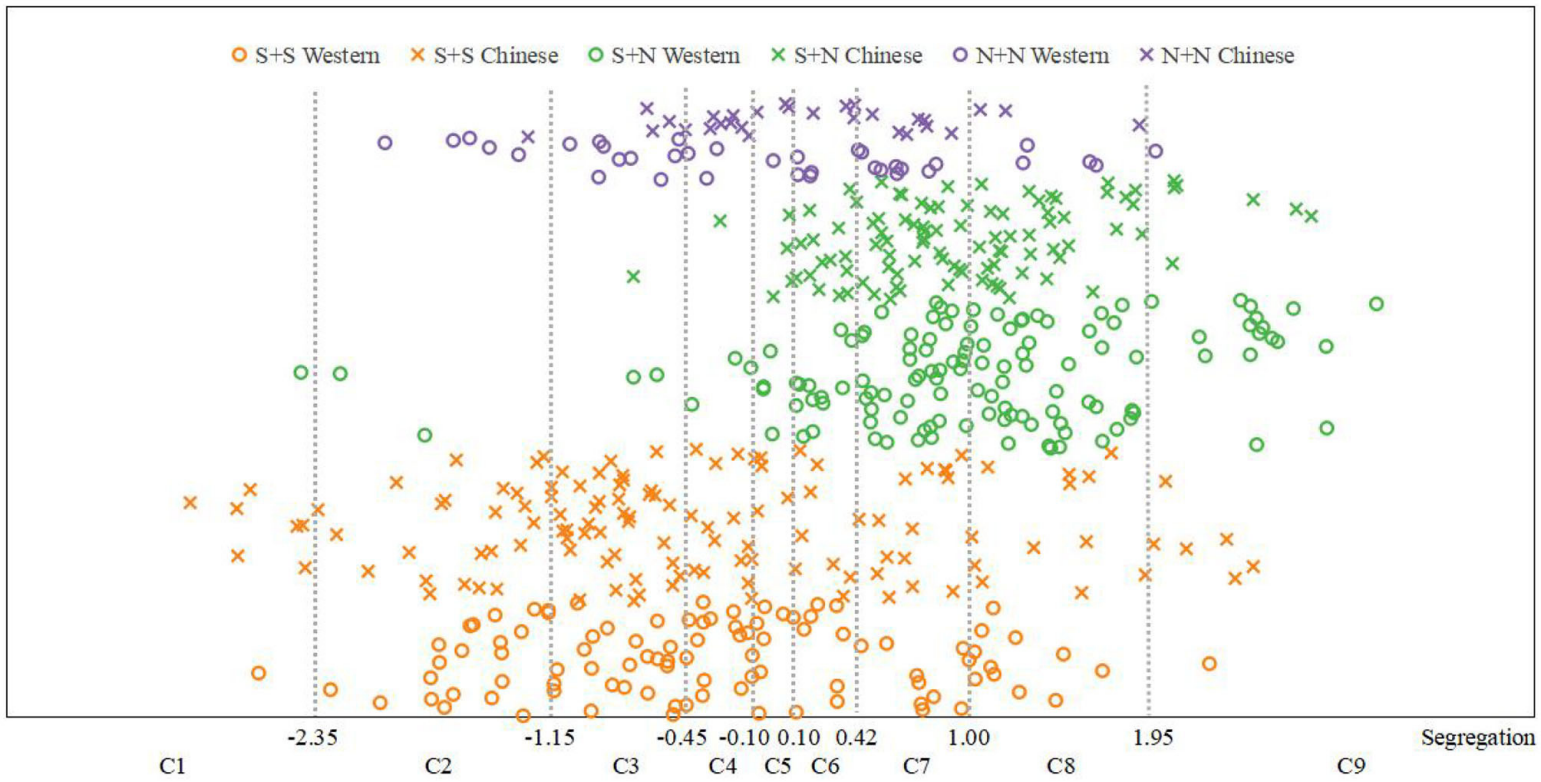
Segregation psychological scales.

**Figure 6:**
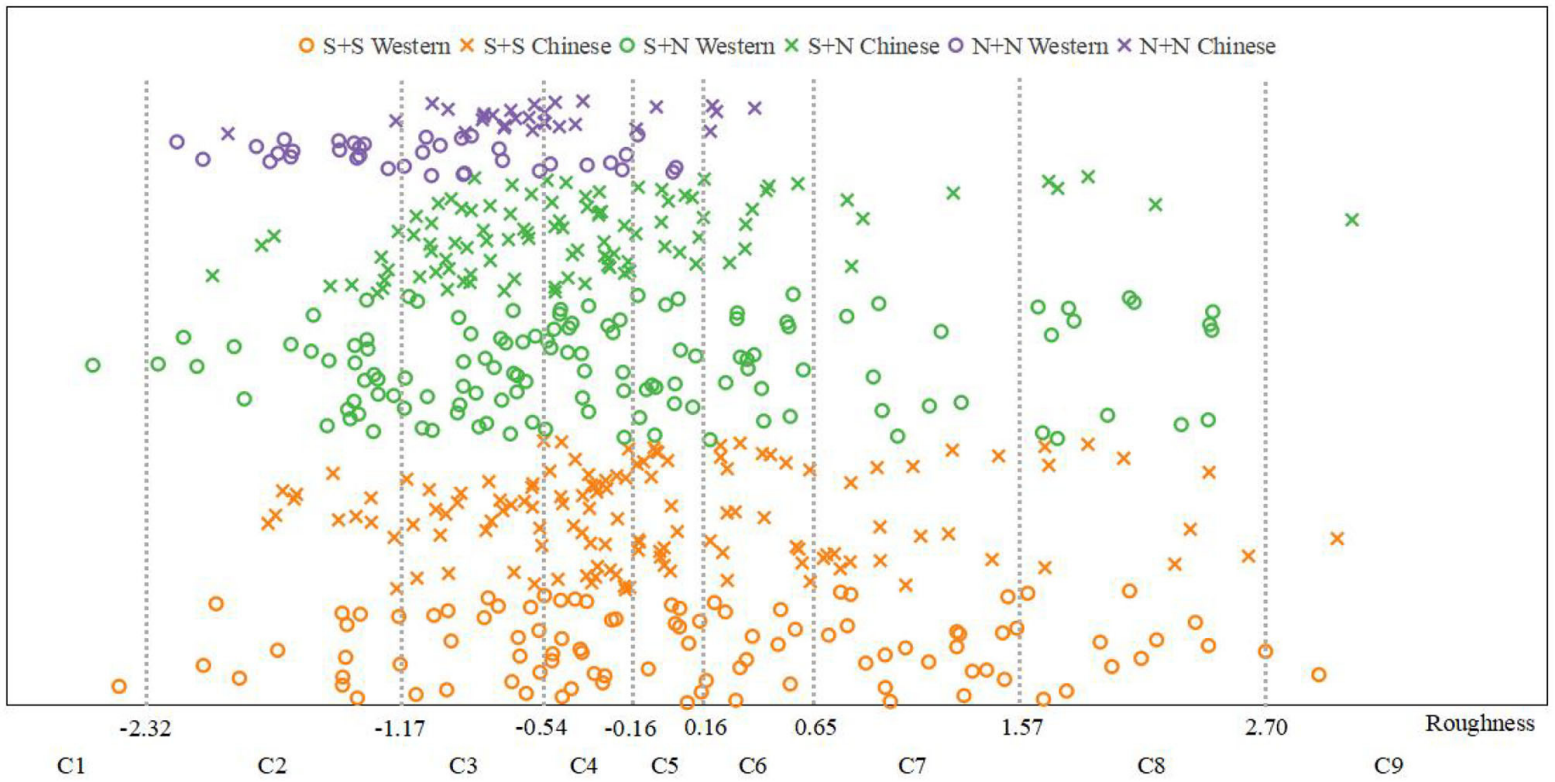
Roughness psychological scales.

**Figure 7:**
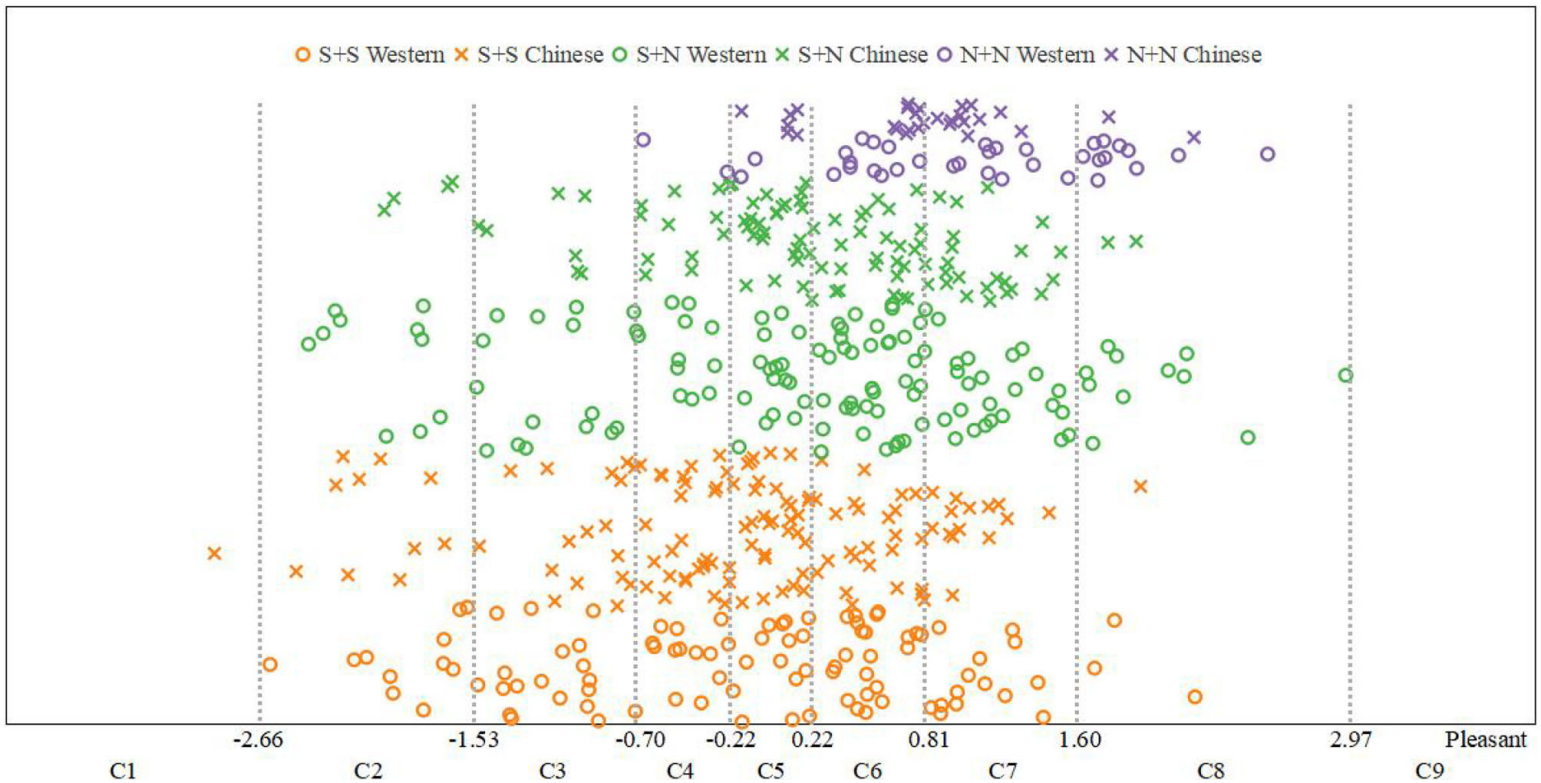
Pleasantness psychological scales.

The category judgment model was used to calculate statistics of the fusion, segregation, roughness, and pleasantness experimental data, and the psychological scale of all samples on each timbre perception attribute was obtained, as shown in Figures 4–7. N + N refers to non-sustaining instruments and non-sustaining instruments, S + N refers to sustaining instruments and non-sustaining instruments, and S + S refers to sustaining instruments and sustaining instruments. The abscissa represents the psychological scale distribution of each dimension, and the ordinate represents the serial number of each stimulus. It can be seen intuitively from the figures that there are certain differences in the distribution of timbre perception attributes with different temporal envelopes. The next section further analyzes these specific differences by combining one-way ANOVA and two-way ANOVA.

### Analysis and Discussion of Experimental Results

The listening test was a 3- × -2 mixed-measures design with two between subjects, the temporal-envelope factors and the instrument-type factors. There were three types of temporal-envelope factors: a combination of sustaining instruments and sustaining instruments (S + S), a combination of sustaining instruments and non-sustaining instruments (S + N), and a combination of non-sustaining instruments and non-sustaining instruments (N + N). There were two types of instrument type factors: Western instruments (W) and Chinese instruments (C).

In this part, the fusion, segregation, roughness, and pleasantness experimental data were analyzed. The analysis idea of each timbre perception dimension was as follows. First, based on the analysis results of the method of successive categories, the timbre combination forms of different temporal-envelope factors were statistically analyzed to compare the timbre perception attributes of different temporal envelopes. Second, one-way ANOVA was used to explore the effects of the temporal-envelope factors and instrument-type factors on each timbre perception attribute. Finally, two-way ANOVA was used to explore the differences in timbre perception attributes under the interaction between the temporal envelopes and instrument types.

#### Fusion

##### Fusion Data Distribution Statistics

**Figure 8:**
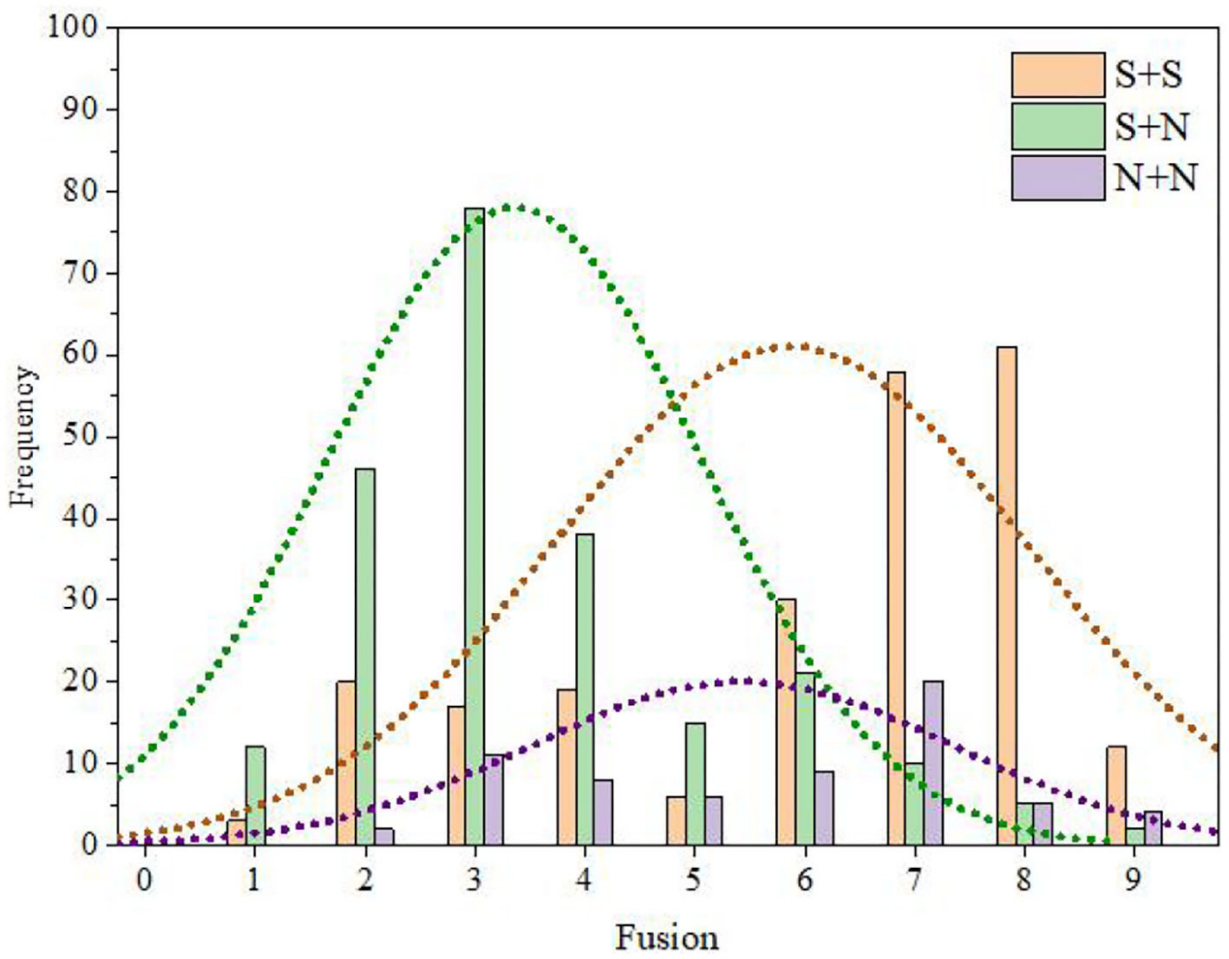
Distribution statistical histogram of fusion category.

To further explore the relationship between the temporal envelope factor and fusion, we calculated the number and percentage of audio stimuli with different temporal envelopes in each category of fusion (Table 5 of the Appendix). Then, the frequency statistical histogram of each category of fusion was drawn according to the data in Table 5 of the Appendix, and the results are shown in Figure 8.

It can be seen from Table 5 of the Appendix and Figure 8 that the fusion of S + S was mainly distributed in categories C6, C7, and C8 and was relatively high (M = 5.97, SD = 3.44). In contrast, the fusion of S + N was mainly distributed in categories C2, C3, and C4 and was relatively low (M = 3.98, SD = 1.61). There were no obvious distribution characteristics for the fusion of N + N instruments, and there was a certain distribution in the range of categories C3–C7 (M = 5.51, SD = 2.07).

**Figure 9:**
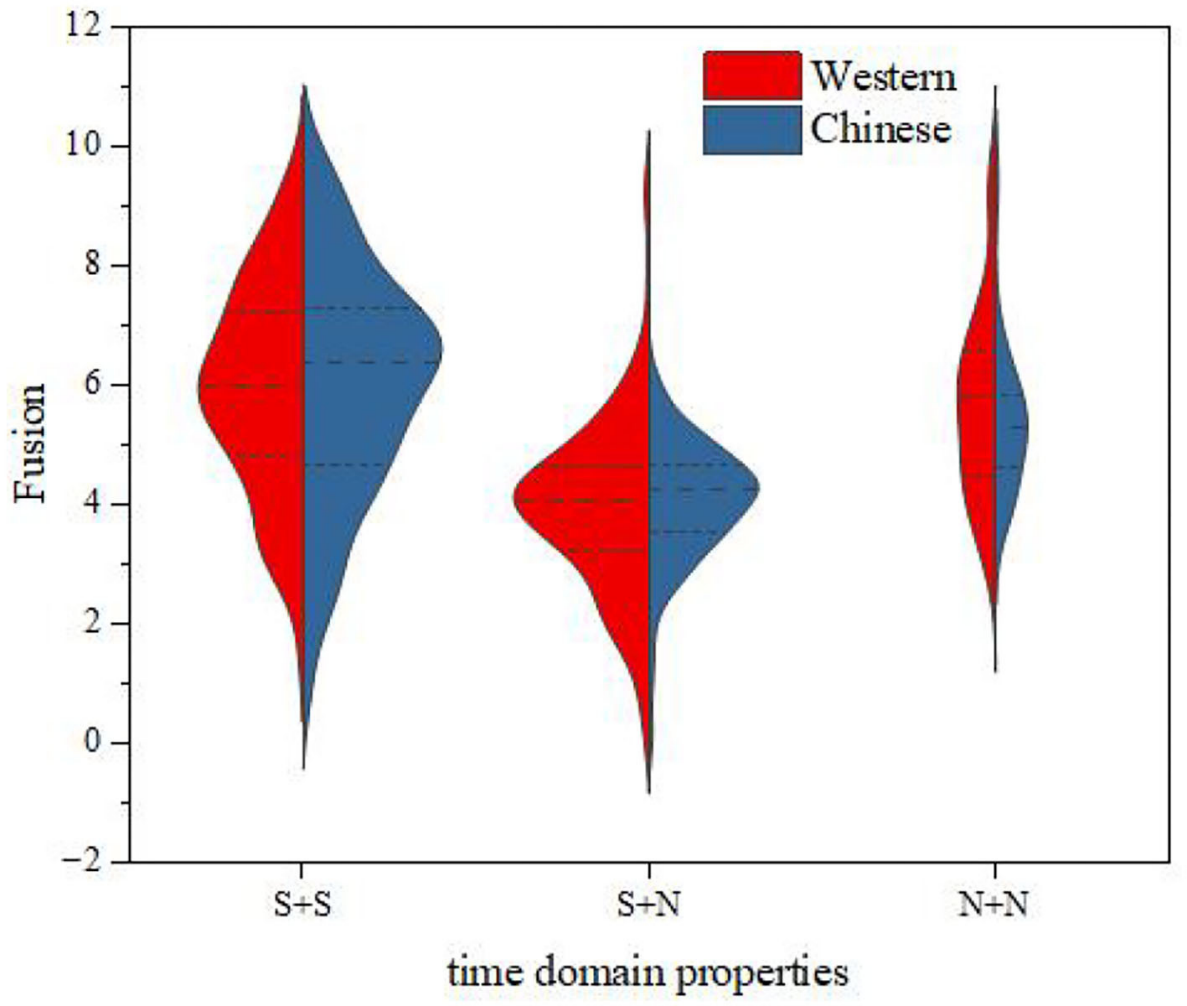
Fusion comparisons between Western and Chinese instruments.

The relationship between the instrument type factor and the distribution of fusion was further discussed. We divided the experimental data into two groups according to the type of a musical instrument (i.e., Chinese instruments and Western instruments) and calculated the statistical characteristics of the fusion for each group. The results are shown in Figure 9. It can be seen from this figure that, for the same temporal envelope, the average fusion values for Chinese instruments and Western instruments were close, and the change law of the fusion from time to time was consistent; that is, the order of fusion from largest to smallest was S + S > N + N > S + N.

##### Influence of Instrument Types and Temporal Envelopes

To explore the influence of instrument types and temporal envelopes on the fusion, the one-way ANOVA model was used for statistical analysis. Before one-way ANOVA, the normality of the experimental fusion data was tested. The experimental data were grouped according to the instrument types and temporal envelopes, the normality of each group of data was tested, and the normal P-P was drawn, as shown in the Figure 2 of the Appendix. In this figure, the ordinate represents the cumulative probability of prediction, and the abscissa represents the cumulative probability of the actual data. If the measured curve is closer to the predicted cumulative probability (i.e., a line with a slope of 1), the actual data distribution is closer to the normal distribution. It can be seen from this figure that the data distribution of fusion met normality for both instrument type and temporal-envelope factors.

First, the experimental data were divided into two groups: Chinese instruments and Western instruments. The data of each group were analyzed by one-way ANOVA. The results are shown in the Table 6 of the Appendix. It can be seen from this table that the temporal envelope had an impact on both Western instruments [*p* < 0.0001, *F*_(2,256)_ = 48.080] and Chinese instruments [*p* < 0.0001, *F*_(2,256)_ = 44.694] with respect to fusion.

We further analyzed the influence of temporal envelopes on the fusion of Chinese and Western instruments and the differences between different temporal envelopes. Here, the Student–Newman–Keuls (SNK) method was used for pairwise comparisons between groups. The results are shown in the Tables 7, 8 of the Appendices.

For Western instruments, the three temporal envelopes were divided into two subgroups. The fusion scores of S + S and N + N were similar, and they were divided into the same subgroup. The significance *p* = 0.326 > 0.05 indicated that there was no difference between the average values of various types in the subgroup. The mean value of fusion in the second subgroup was greater than that in the first subgroup. For Chinese instruments, the three temporal envelopes were divided into three subgroups: S + S, S + N, and N + N. The fusion scores of the three subgroups were different, and the order of fusibility from largest to smallest was S + S > N + N > S + N.

From the above results, it can be concluded that the three temporal envelopes of both Chinese and Western instruments have an impact on the fusion. Moreover, temporal envelopes have a greater impact on the fusion of Chinese instruments but lesser impact on the fusion of Western instruments.

Then, we analyzed the factors of instrument type. The experimental data were divided into three groups: S + S, S + N, and N + N. The results are shown in the Table 9 of the Appendix. It can be seen from this table that under, any time domain property condition, the significant *P*-value of instrument types was >0.05 [S + S: *p* = 0.863 > 0.05, *F*_(1,224)_ = 0.030; S + N: *p* = 0.564 > 0.05, *F*_(1,225)_ = 0.334; N + N: *p* = 0.319 > 0.05, *F*_(1,63)_ = 1.008], indicating that there is no difference in the fusion score between Chinese and Western instruments under the three time domain property conditions; that is, the instrument type has no effect on the fusion.

##### Interaction Between Temporal Envelopes and Instrument Types

The above one-way ANOVA only considered the difference in fusion under the same factor. Next, we further studied the analysis model, considering both temporal envelopes and instrument types. Here, the two-way ANOVA model was used to analyze the fusion. Similar to one-way ANOVA, two-way ANOVA also requires normality testing. The P-P diagram of the normal probability distribution (normal P–P) was calculated and drawn, as shown in the Figure 3 of the Appendix. It can be seen from this figure that the measured curve was close to the predicted cumulative probability, indicating that the distribution of the experimental data met normality.

The results of two-way ANOVA for the experimental data are shown in the Table 10 of the Appendix. It can be seen from this table that the significance of instrument types and temporal envelopes was >0.05 (*p* = 0.581 > 0.05), indicating that the interaction between instrument types and temporal envelopes was not statistically significant. To make the model more concise, this interaction can be removed from the model, and the model can be fitted with only the main effect. The results are shown in the Table 11 of the Appendix. This table shows that the instrument types [*p* = 0.906 > 0.05, *F*_(5,512)_ = 0.014] had no effect on fusion, while the temporal envelope [*p* < 0.0001, *F*_(5,512)_ = 92.469] had an effect on fusion. That is, whether Chinese or Western instruments are utilized, the temporal envelope impacts the fusion. This conclusion is the same as that of one-way ANOVA, which further explains the relationship between the temporal envelope and fusion.

Combining the results of the descriptive statistical analyses, one-way ANOVA and two-way ANOVA, we can draw the following conclusions: (1) The temporal envelopes have a certain influence on the fusion; that is, the fusion of different temporal envelopes is different. The instrument types have no effect on fusion. For both Chinese and Western instruments, the order of fusion from largest to smallest is S + S > N + N > S + N. There is no significant difference in the ranking trend of fusion between Chinese and Western instruments. (2) An interaction between temporal envelopes and instrument types has not been found; that is, the difference in fusion between different temporal envelopes is, basically, the same in different instrument types.

#### Segregation

##### Segregation Data Distribution Statistics

**Figure 10:**
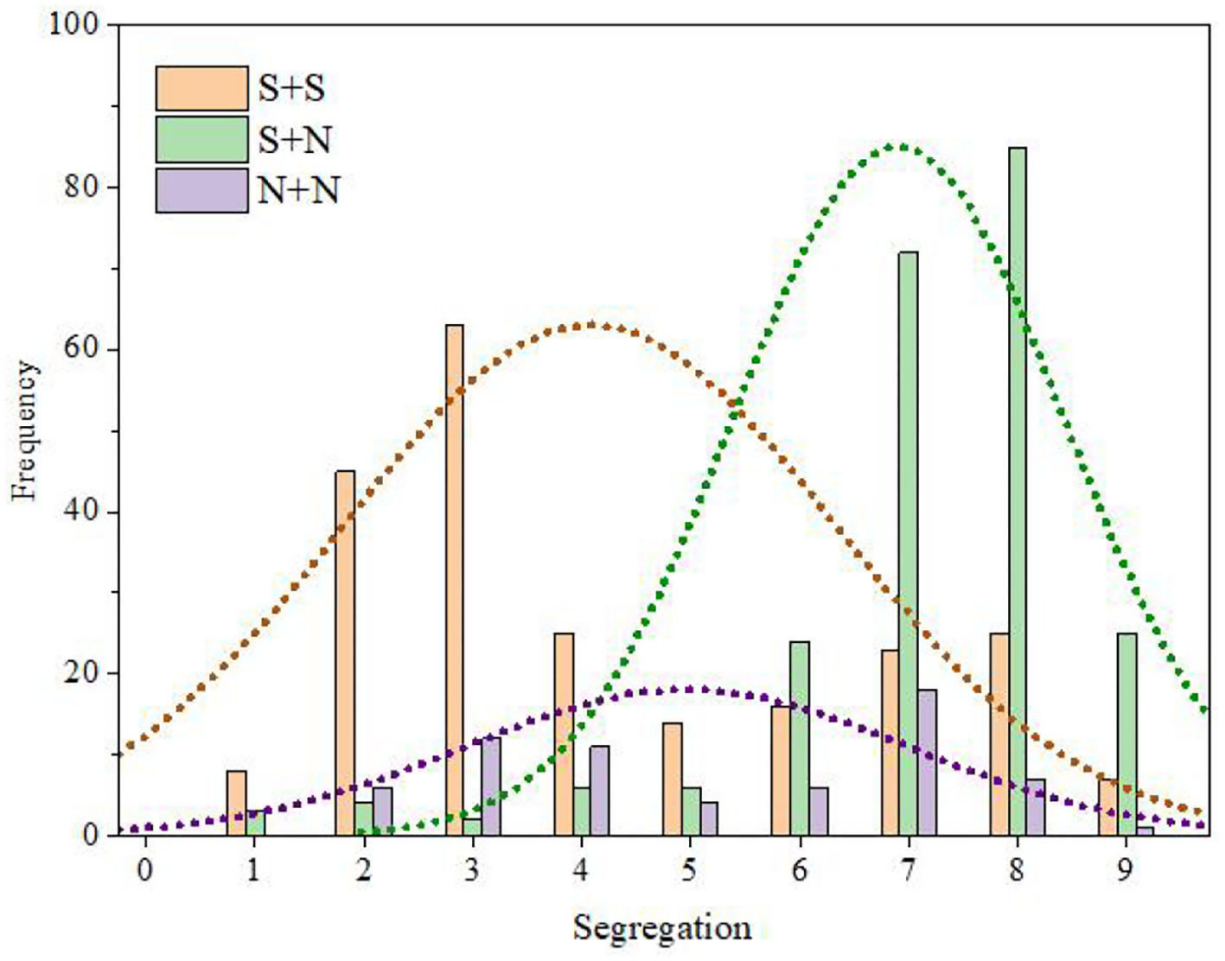
Distribution statistical histogram of segregation category.

Using the same methods as those used for fusion degree analysis, we obtained the frequency distribution statistics of each category of segregation (Table 12 of the Appendix) and the distribution statistical histogram of the segregation category (Figure 10).

It can be seen from Table 12 of the Appendix and Figure 10 that the segregation of S + S was mainly distributed in categories C2, C3, and C4 and was relatively low (M = 4.35, SD = 3.22). In contrast, the segregation of S + N was mainly distributed in categories C7 and C8 and was relatively high (M = 6.53, SD = 1.67). The fusion of N + N did not show obvious distribution characteristics, and there was a certain distribution in the range of categories C2–C8 (M = 5.01, SD = 1.97).

**Figure 11:**
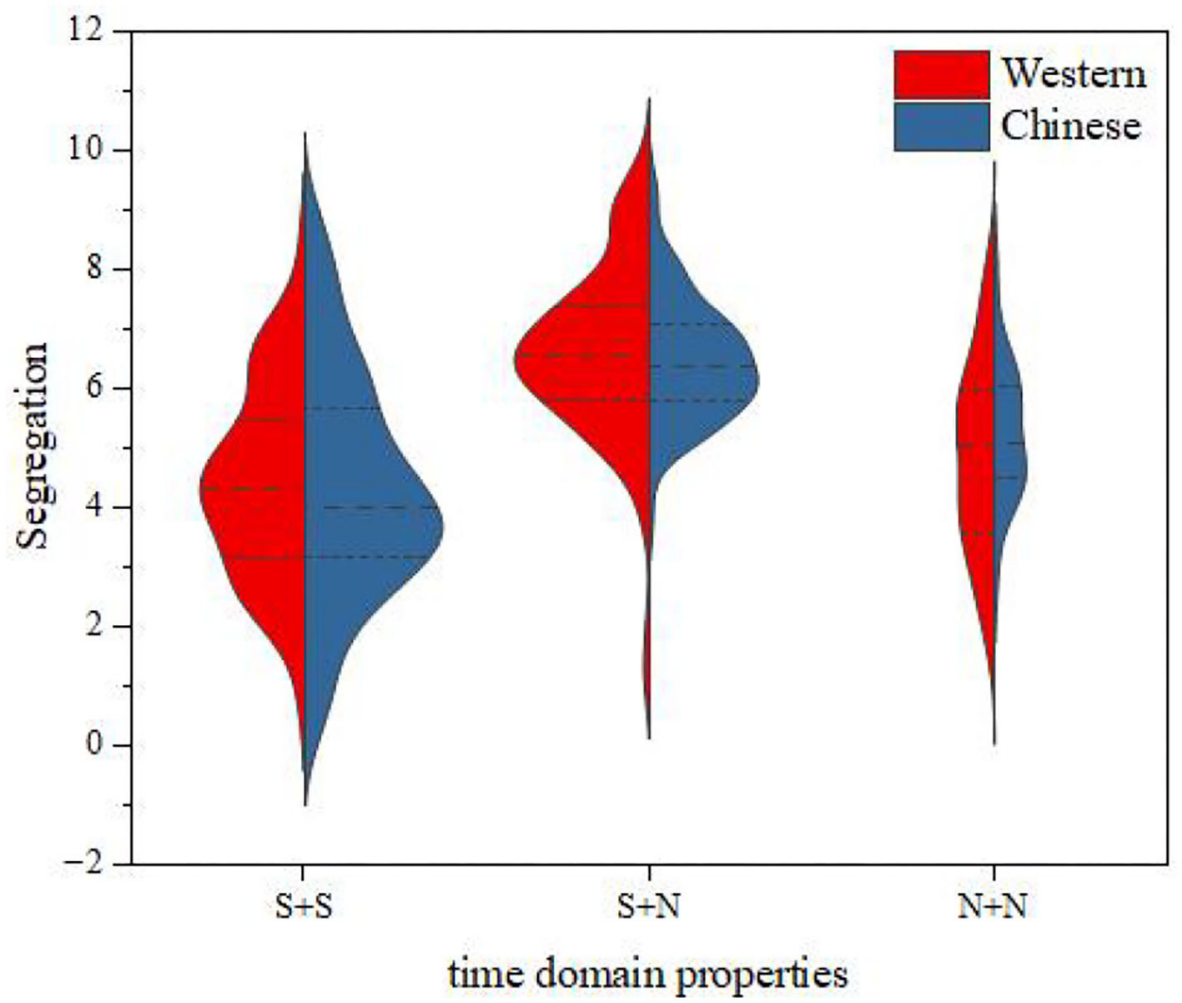
Segregation comparisons between Western and Chinese instruments.

The relationship between the instrument-type factor and the distribution of segregation was further discussed by the same method as that used for fusion degree analysis. The results are shown in Figure 11. It can be seen from this figure that, for the same temporal envelope, the average values of the segregation for Chinese instruments and Western instruments were close, and the change law of the segregation from time to time was consistent; that is, the order of the segregation from largest to smallest was S + N > N + N > S + S.

##### Influence of Instrument Types and Temporal Envelopes

We tested the normality of the experimental data of segregation and then analyzed them by one-way ANOVA. The results are shown in Figure 4 and Table 13 of the Appendices. It can be seen from this table that the temporal envelope had an impact on both Western instruments [*p* < 0.0001, *F*_(2,256)_ = 58.651] and Chinese instruments [*p* < 0.0001, *F*_(2,256)_ = 54.167] with respect to segregation.

We further analyzed the influence of temporal envelopes on the segregation of Chinese and Western instruments and the differences between different temporal envelopes. Here, the Student–Newman–Keuls (SNK) method was used for pairwise comparisons between groups. The results are shown in the Tables 14, 15 of the Appendices.

For Western instruments, the three temporal envelopes were divided into two subgroups. The segregation scores of S + S and N + N were similar, and they were divided into the same subgroup. There was no difference between the average values of various types in the subgroup (*p* = 0.108 > 0.05). The mean value of segregation in the second subgroup was greater than that in the first subgroup. For Chinese instruments, the three temporal envelopes were divided into three subgroups: S + S, S + N, and N + N. The segregation scores of the three were different, and the order of segregation from largest to smallest was S + N > N + N > S + S.

From the above results, it can be concluded that the three temporal envelopes of both Chinese and Western instruments have an impact on segregation. Moreover, temporal envelopes have a greater impact on the segregation of Chinese instruments but a lesser impact on the segregation of Western instruments.

Then, we analyzed the factors of instrument type. The experimental data were divided into three groups: S + S, S + N, and N + N. The results are shown in the Table 16 of the Appendix. It can be seen from the table that, under any time domain property condition, the significant P value of instrument types was > 0.05 [S + S: *p* = 0.732 > 0.05, *F*_(1,224)_ = 0.117; S + N: *p* = 0.505 > 0.05, *F*_(1,225)_ = 0.445; N + N: *p* = 0.268 > 0.05, *F*_(1,63)_ = 1.249], indicating that there is no difference in the segregation score between Chinese and Western instruments under the three time domain property conditions; that is, the instrument type has no effect on the segregation.

##### Interaction Between Temporal Envelopes and Instrument Types

The above one-way ANOVA only considered the difference in segregation under the same factor. Next, we further studied the analysis model, considering both temporal envelopes and instrument types. Here, the two-way ANOVA model was used to analyze segregation. Similar to one-way ANOVA, two-way ANOVA also requires normality testing. The P–P diagram of the normal probability distribution (normal P–P) was calculated and drawn, as shown in the Figure 5 of the Appendix. It can be seen from this figure that the measured curve was close to that of the predicted cumulative probability, indicating that the distribution of the experimental data met normality.

The results of two-way ANOVA for the experimental data are shown in the Table 17 of the Appendix. It can be seen from this table that the significance of instrument types and temporal envelopes was >0.05 (*p* = 0.492 > 0.05), indicating that the interaction between instrument types and temporal envelopes was not statistically significant. To make the model more concise, this interaction can be removed from the model, and the model can be fitted with only the main effect. The results are shown in the Table 18 of the Appendix. This table shows that the instrument types [*p* = 0.785 > 0.05, *F*_(5,512)_ = 0.075] had no effect on segregation, while the temporal envelope [*p* < 0.0001, *F*_(5,512)_ = 112.211] had an effect on segregation. That is, whether Chinese or Western instruments are utilized, the temporal envelope impacts segregation. This conclusion is the same as that of one-way ANOVA, which further explains the relationship between the temporal envelope and segregation.

Combining the results of the descriptive statistical analyses, one-way ANOVA and two-way ANOVA, we can draw the following conclusions: (1) The temporal envelopes have a certain influence on segregation; that is, the segregation of different temporal envelopes is different. The instrument types have no effect on segregation. For both Chinese and Western instruments, the order of segregation from largest to smallest is S + N > N + N > S + S. There is no significant difference in the ranking trend of segregation between Chinese and Western instruments. (2) An interaction between temporal envelopes and instrument types has not been found; that is, the difference in segregation between different temporal envelopes is, basically, the same for different instrument types.

#### Roughness

##### Roughness Data Distribution Statistics

**Figure 12:**
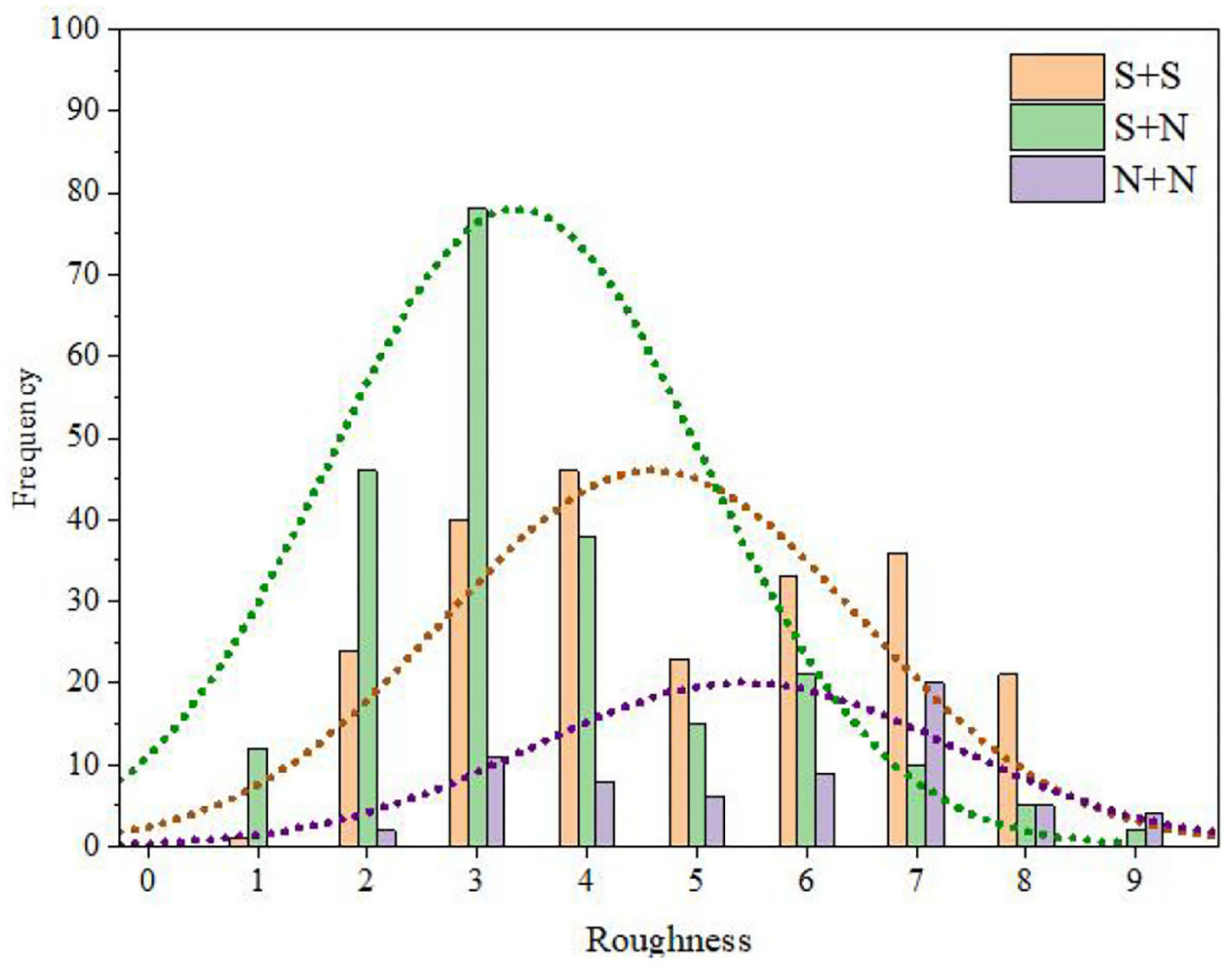
Distribution statistical histogram of roughness category.

Using the same methods as those used for fusion degree analysis, we obtained the frequency distribution statistics of each category of roughness (Table 19 of the Appendix) and the distribution statistical histogram of the roughness category (Figure 12).

It can be seen from Table 19 of the Appendix and Figure 12 that the roughness of S + S was mainly distributed in categories C3 and C4 and was relatively high (M = 4.96, SD = 2.94). In contrast, the roughness of S + N was mainly distributed in categories C2, C3, and C4 and was higher (M = 4.42, SD = 2.60). The roughness of N + N was mainly distributed in categories C3, C4, and C5 and was relatively low (M = 3.54, SD = 0.93).

**Figure 13:**
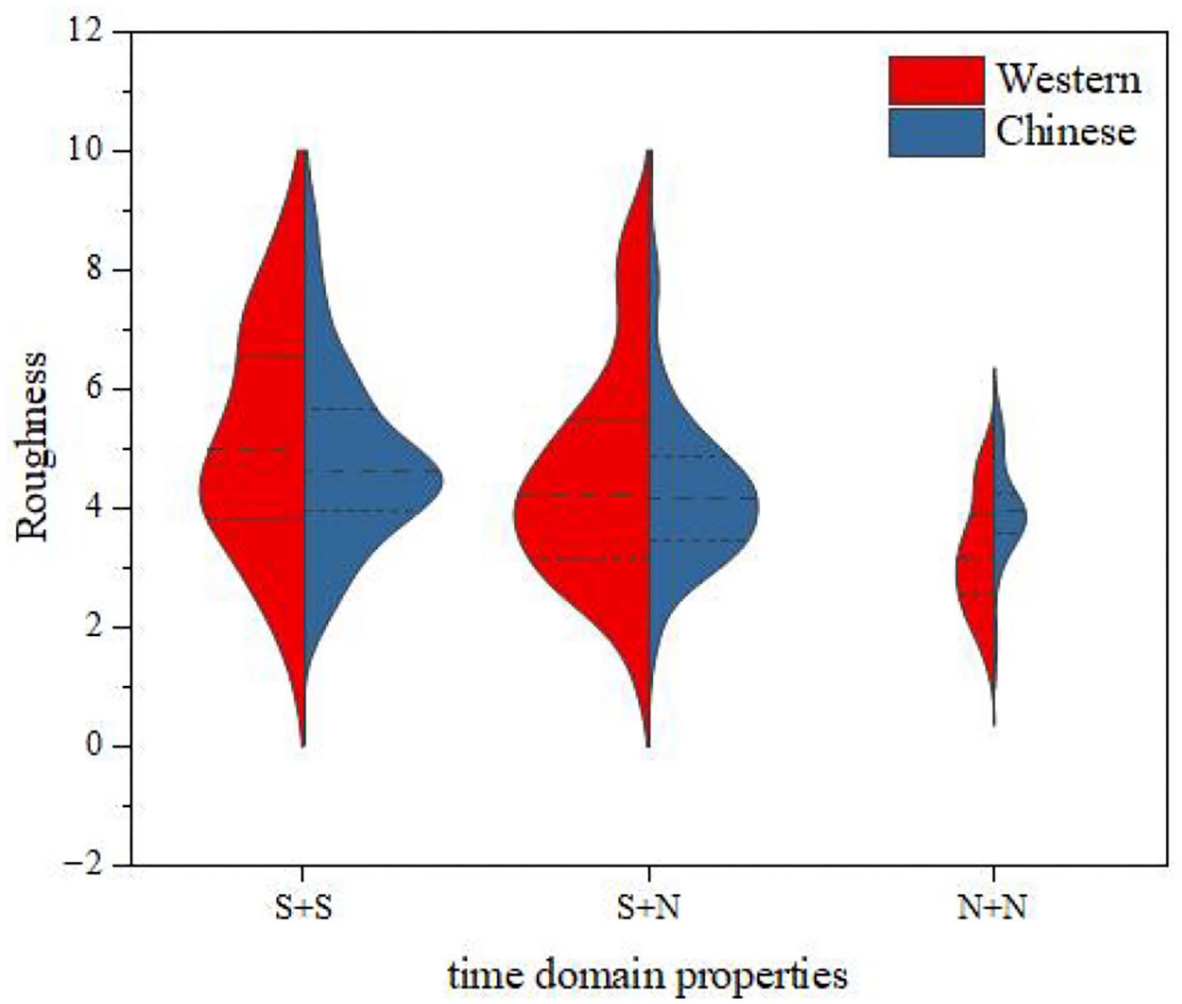
Roughness comparisons between Western and Chinese instruments.

The relationship between the instrument-type factors and the distribution of roughness was further discussed by the same method as that used for fusion degree analysis. The results are shown in Figure 13. It can be seen from this figure that, for S + S and S + N, the mean roughness values for Chinese instruments and Western instruments were close, and the variation law of roughness from time to time was consistent; for N + N, the mean roughness of Chinese instruments was larger than that for Western instruments. However, for all western and Chinese instruments, the roughness still had the same law from largest to smallest, i.e., S + S > S + N > N + N.

##### Influence of Instrument Types and Temporal Envelope

Similarly, we tested the normality of the experimental roughness data and then analyzed them by one-way ANOVA. The results are shown in Figure 6 and Table 20 of the Appendices. It can be seen from this table that the temporal envelope had an impact on both Western instruments [*p* < 0.0001, *F*_(2,256)_ = 15.902] and Chinese instruments [*p* = 0.0001, *F*_(2,256)_ = 6.833] with respect to roughness.

We further analyzed the influence of temporal envelopes on the roughness of Chinese and Western instruments and the differences between different temporal envelopes. Here, the Student–Newman–Keuls (SNK) method was used for pairwise comparisons between groups. The results are shown in the (Tables 21, 22 of the Appendices).

For Western instruments, the three temporal envelopes were divided into three subgroups: S + S, S + N and N + N. The roughness scores of the three subgroups were different, and the order of roughness from largest to smallest was S + S > S + N > N + N. For Chinese instruments, the three temporal envelopes were divided into two subgroups: the roughness scores of S + N and N + N were similar, so they were divided into the same subgroup. There was no difference between the average values of various types in the subgroup (*p* = 0.209 > 0.05). The mean value of roughness in the second subgroup was greater than that in the first subgroup.

From the above results, it can be concluded that the three temporal envelopes of both Chinese and Western instruments have an impact on the roughness. Moreover, temporal envelopes have a greater impact on the roughness of Western instruments but a lesser impact on the roughness of Chinese instruments.

Then, we analyzed the factors of instrument type. The experimental data were divided into three groups: S + S, S + N, and N + N. The results are shown in the (Table 23 of the Appendix). It can be seen from this table that, when the time domain property condition was S + S or S + N, the significant *P*-value of instrument type was >0.05 [S + S: *p* = 0.249 > 0.05, *F*_(1,224)_ = 1.335; S + N: *p* = 0.369 > 0.05, *F*_(1,225)_ = 0.809], indicating that there was no difference in the roughness score between Chinese and Western instruments. However, when the time domain property condition was N + N [*p* = 0.001 < 0.05, *F*_(1,63)_ = 13.010] there were some differences between Chinese and Western instruments under the three time domain property conditions; that is, the instrument type affected the roughness when the time domain property condition was N + N.

##### Interaction Between Temporal Envelope and Instrument Types

The above one-way ANOVA only considered the difference in roughness under the same factor. Next, we further studied the analysis model, considering both temporal envelopes and instrument types. Here, the two-way ANOVA model was used to analyze roughness. Similar to one-way ANOVA, two-way ANOVA also requires normality testing. The P–P diagram of the normal probability distribution (normal P–P) was calculated and drawn, as shown in the Figure 7 of the Appendix. It can be seen from this figure that the measured curve was close to the predicted cumulative probability, indicating that the distribution of the experimental data met normality.

The results of two-way ANOVA of the experimental data are shown in the Table 24 of the Appendix. It can be seen from this table that the significance of instrument types and temporal envelopes was greater than 0.05 (*p* = 0.053 > 0.05), indicating that the interaction between instrument types and temporal envelopes was not statistically significant. To make the model more concise, this interaction can be removed from the model, and the model can be fitting with only the main effect. The results are shown in the Table 25 of the Appendix. The table shows that the instrument types [*p* = 0.478 > 0.05, *F*_(5,512)_ = 0.505] had no effect on the roughness, while the temporal envelope [*p* < 0.0001, *F*_(5,512)_ = 21.654] had an effect on the roughness. That is, whether Chinese or Western instruments are utilized, the temporal envelope has an impact on the roughness. This conclusion is the same as that of one-way ANOVA, which further explains the relationship between temporal envelopes and roughness.

Combining the results of the descriptive statistical analyses, one-way ANOVA and two-way ANOVA, we can draw the following conclusions: (1) The temporal envelopes have a certain influence on the roughness, that is, the roughness of different temporal envelopes are different. For both Chinese and Western instruments, the order of roughness from largest to smallest is S + S > N + N > S + N. There is no significant difference in the ranking trend of roughness between Chinese and Western instruments. (2) Interactions between temporal envelopes and instrument types have not been found; however, when the time domain property is N + N, the instrument type has an effect on the roughness. That is, when the time domain property is N + N, the roughness of Chinese instruments is larger than that of Western instruments.

#### Pleasantness

##### Pleasantness Data Distribution Statistics

**Figure 14:**
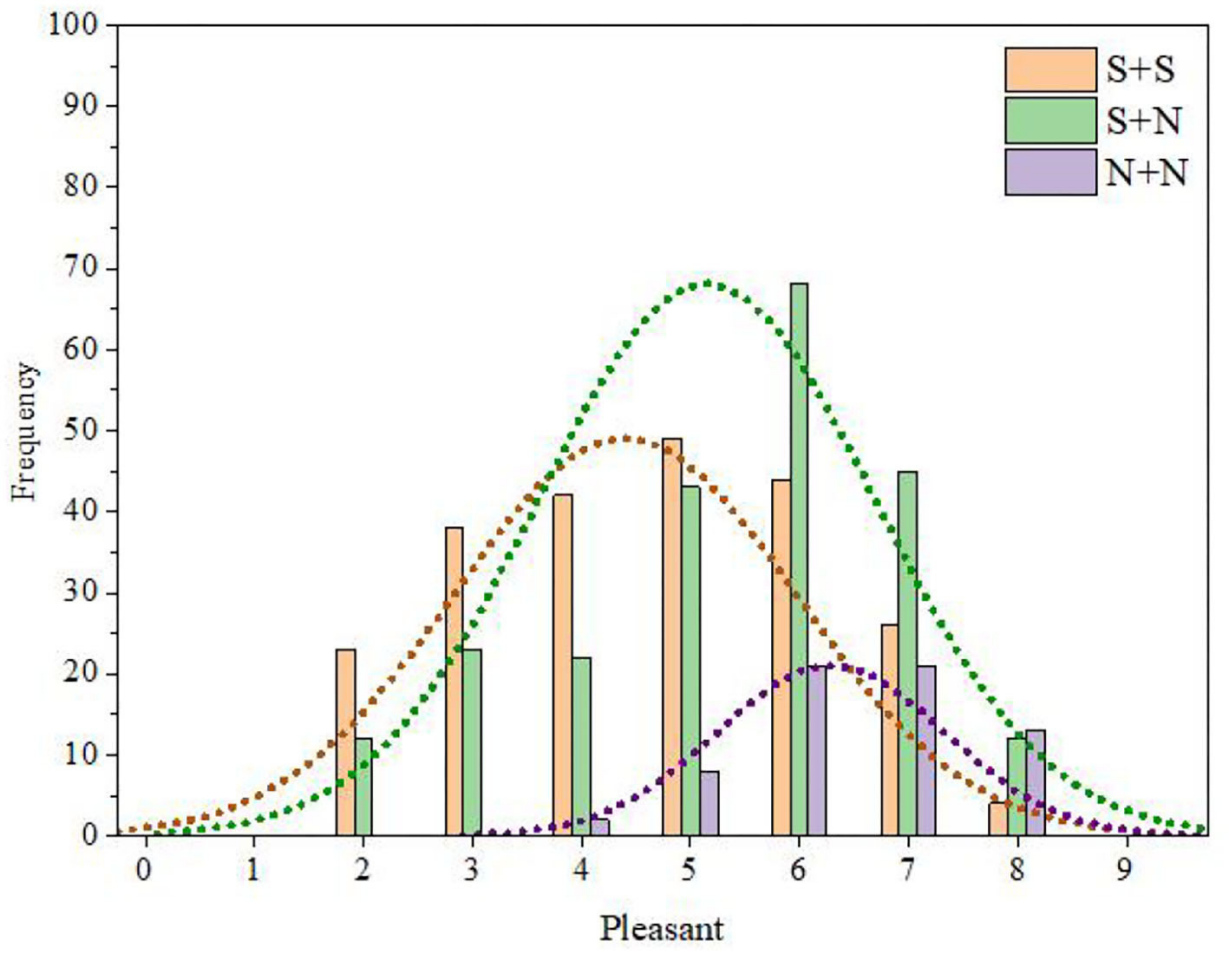
Distribution of statistical histogram of pleasantness category.

Using the same methods as those used for fusion degree analysis, we obtained the frequency distribution statistics of each category of pleasantness (Table 26 of the Appendix) and the distribution statistical histogram of the pleasantness category (Figure 14).

It can be seen from Table 26 of the Appendix and Figure 14 that the pleasantness of S + S was mainly distributed in categories C3–C6 and was relatively low (M = 4.64, SD = 2.23). In contrast, the pleasantness of S + N was mainly distributed in categories C5–C7 and was relatively high (M = 5.26, SD = 2.43). The pleasantness of N + N was mainly distributed in categories C6–C8 and was higher (M = 6.36, SD = 1.07).

**Figure 15:**
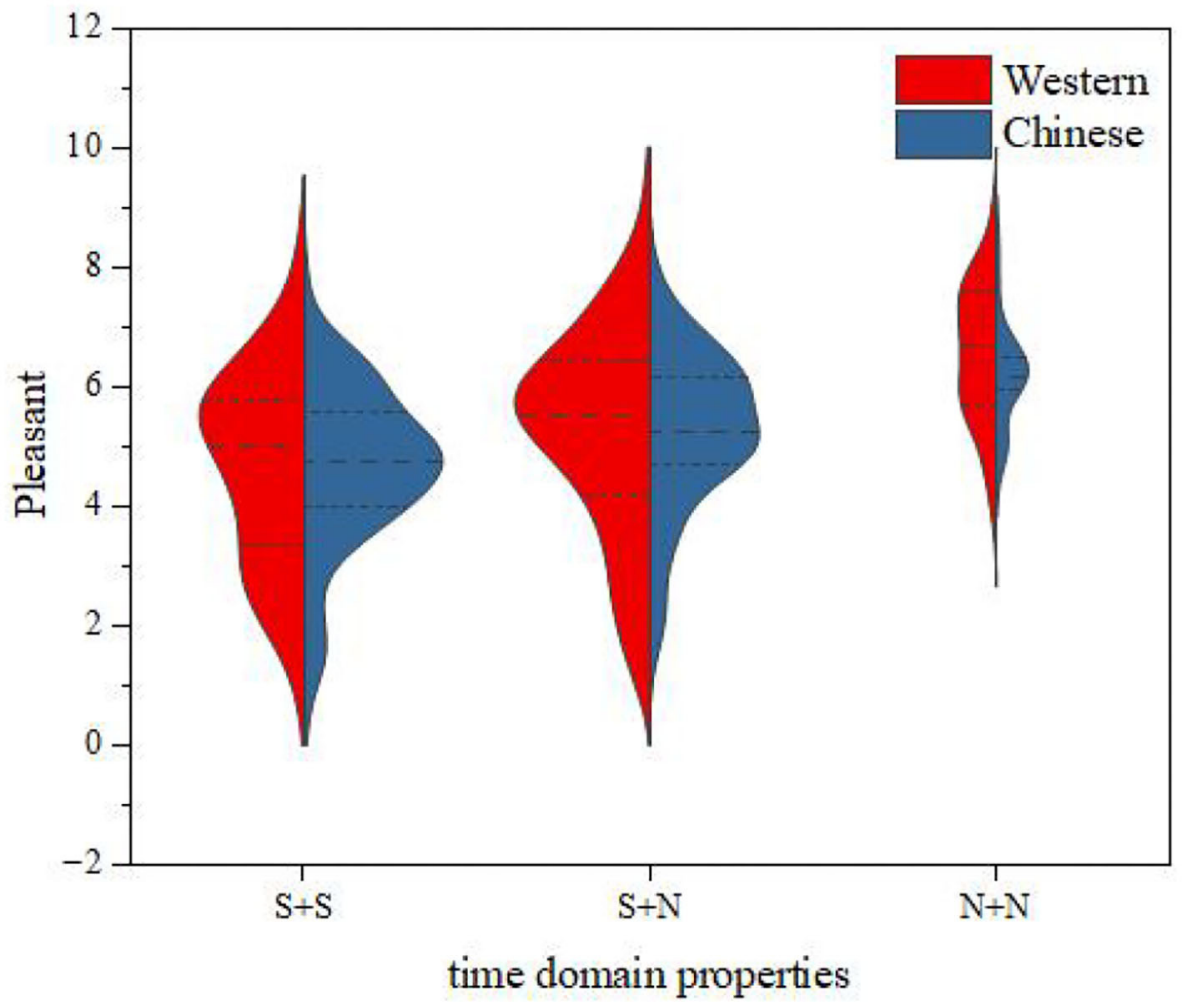
Pleasantness comparisons between Western and Chinese instruments.

The relationship between the instrument-type factors and the distribution of pleasantness was further discussed by the same method as that used for fusion degree analysis. The results are shown in Figure 15. It can be seen from this figure that, for the same temporal envelope, the average values of pleasantness for Chinese instruments and Western instruments were close, and the change law of pleasantness from time to time was consistent; that is, the order of pleasantness from largest to smallest was N + N > S + N > S + S.

##### Influence of Instrument Types and Temporal Envelope

Similarly, we tested the normality of the experimental pleasantness data and then analyzed them by one-way ANOVA. The results are shown in Figure 8 and Table 27 of the Appendices. It can be seen from this table that the temporal envelope had an impact on both Western instruments [*p* < 0.0001, *F*_(2,256)_ = 17.417] and Chinese instruments [*p* < 0.0001, *F*_(2,256)_ = 18.807] with respect to pleasantness.

We further analyzed the influence of temporal envelopes on the pleasantness of Chinese and Western instruments and the differences between different temporal envelopes. Here, the Student–Newman–Keuls (SNK) method was used for pairwise comparisons between groups. The results are shown in the Tables 28, 29 of the Appendices.

For Western instruments, the three temporal envelopes were divided into three subgroups: S + S, S + N, and N + N. The pleasantness scores of the three subgroups were different, and the order of pleasantness from largest to smallest was N + N > S + N > S + S. For Chinese instruments, the three temporal envelopes were also divided into three subgroups: S + S, S + N, and N + N. The pleasantness scores of the three subgroups were different, and the order of pleasantness from largest to smallest was N + N > S + N > S + S.

From the above results, it can be concluded that the three temporal envelopes of both Chinese and Western instruments have the same impact on pleasantness.

Then, we analyzed the factors of instrument type. The experimental data were divided into three groups: S + S, S + N, and N + N. The results are shown in the Table 30 of the Appendix. It can be seen from this table that, under any time domain property condition, the significant *P*-value of instrument type was >0.05 [S + S: *p* = 0.926 > 0.05, *F*_(1,224)_ = 0.009; S + N: *p* = 0.900 > 0.05, *F*_(1,255)_ = 0.016; N + N: *p* = 0.148 > 0.05, *F*_(1.63)_ = 2.147], indicating that there is no difference in the pleasantness score between Chinese and Western instruments under the three time domain property conditions, that is, the instrument type has no effect on the pleasantness.

##### Interaction Between Temporal Envelope and Instrument Types

The above one-way ANOVA only considered the difference in pleasantness under the same factor. Next, we further studied the analysis model, considering both temporal envelopes and instrument types. Here, the two-way ANOVA model was used to analyze pleasantness. Similar to one-way ANOVA, two-way ANOVA also requires normality testing. The P–P diagram of the normal probability distribution (normal P-P) was calculated and drawn, as shown in the Figure 9 of the Appendix. It can be seen from this figure that the measured curve was close to that of the predicted cumulative probability, indicating that the distribution of the experimental data met normality.

The results of two-way ANOVA of the experimental data are shown in the Table 31 of the Appendix. It can be seen from this table that the significance of instrument types and temporal envelopes was >0.05 (*p* = 0.624 > 0.05), indicating that the interaction between instrument types and temporal envelopes was not statistically significant. To make the model more concise, this interaction can be removed from the model, and the model can be refitting with only the main effect. The results are shown in the Table 32 of the Appendix. This table shows that the instrument types [*p* = 0.738 > 0.05, *F*_(5,512)_ = 0.112] had no effect on pleasantness, while the temporal envelope [*p* < 0.0001, *F*_(5,512)_ = 35.505] had an effect on pleasantness. That is, whether Chinese or Western instruments are utilized, the temporal envelope has an impact on pleasantness. This conclusion is the same as that of one-way ANOVA, which further explains the relationship between temporal envelope and pleasantness.

Combining the results of the descriptive statistical analyses, one-way ANOVA and two-way ANOVA, we can draw the following conclusions: (1) The temporal envelopes have a certain influence on pleasantness; that is, the pleasantness of different temporal envelopes is different. The instrument types have no effect on pleasantness. For both Chinese and Western instruments, the order of pleasantness from largest to smaller is N + N > S + N > S + S. There is no significant difference in the ranking trend of pleasantness between Chinese and Western instruments. (2) An interaction between temporal envelopes and instrument types has not been found; that is, the difference in pleasantness between different temporal envelopes is, basically, the same in different instrument types.

#### Interaction of Timbre Perception Attributes

Here, the correlation analysis was carried out by using the Pearson's correlation coefficient for four timbre perception attributes, fusion, segregation, roughness, and pleasantness. The correlation matrix and test results can be calculated by a two-tailed test, as shown in Table 2. As seen from this table, there is a strong negative correlation between fusion and segregation (*r* = −0.94, Sig < 0.01), indicating that these two attributes tend to move in opposite directions. There is also a strong negative correlation between roughness and pleasantness (*R* = −0.0.94, Sig < 0.01), indicating that these two attributes tend to move in opposite directions. In addition to the above two pairs of strong correlations, other correlations among the four timbre perception attributes were weak. To further analyze the relationship between these attributes, multidimensional preference analysis was used to process the experimental data.

**Table 2:** Correlation matrix and test results of timbre perception attributes.

		**Fusion**	**Segregation**	**Roughness**	**Pleasantness**
Fusion	Pearson correlation	1	−0.945^**^	−0.471^**^	0.471^**^
	Sig. (2-tailed)		*p* < 0.0001	*p* < 0.0001	*p* < 0.0001
Segregation	Pearson correlation	−0.945^**^	1	0.371^**^	−0.370^**^
	Sig. (2-tailed)	*p* < 0.0001		*p* < 0.0001	*p* < 0.0001
Roughness	Pearson correlation	−0.471^**^	0.371^**^	1	−0.892^**^
	Sig. (2-tailed)	*p* < 0.0001	*p* < 0.0001		*p* < 0.0001
Pleasantness	Pearson Correlation	0.471^**^	−0.370^**^	−0.892^**^	1
	Sig. (2-tailed)	*p* < 0.0001	*p* < 0.0001	*p* < 0.0001	

** *Correlation is significant at the 0.01 level (2-tailed)*.

Multidimensional preference analysis is also called principal component analysis of classified data. The principle of this algorithm is to combine the idea of optimal scaling transformation and principal component analysis. In essence, this method is an extension of factor analysis and principal component analysis (Bechtel, 2019). Compared with principal component analysis and factor analysis, multidimensional preference analysis has several advantages. Considering various possible factors in data collection, the optimal scaling technique was introduced in multidimensional preference analysis. This allows the analysis of distance (continuous) variables and order (discrete) variables (such as rating and ranking), thus greatly broadening the application scope of this method. In addition, the results of multidimensional preference analysis can be intuitively presented in the form of a perception map. In other words, the sample and variable loadings can be plotted directly on a single diagram, making it easier to read information from it.

**Figure 16:**
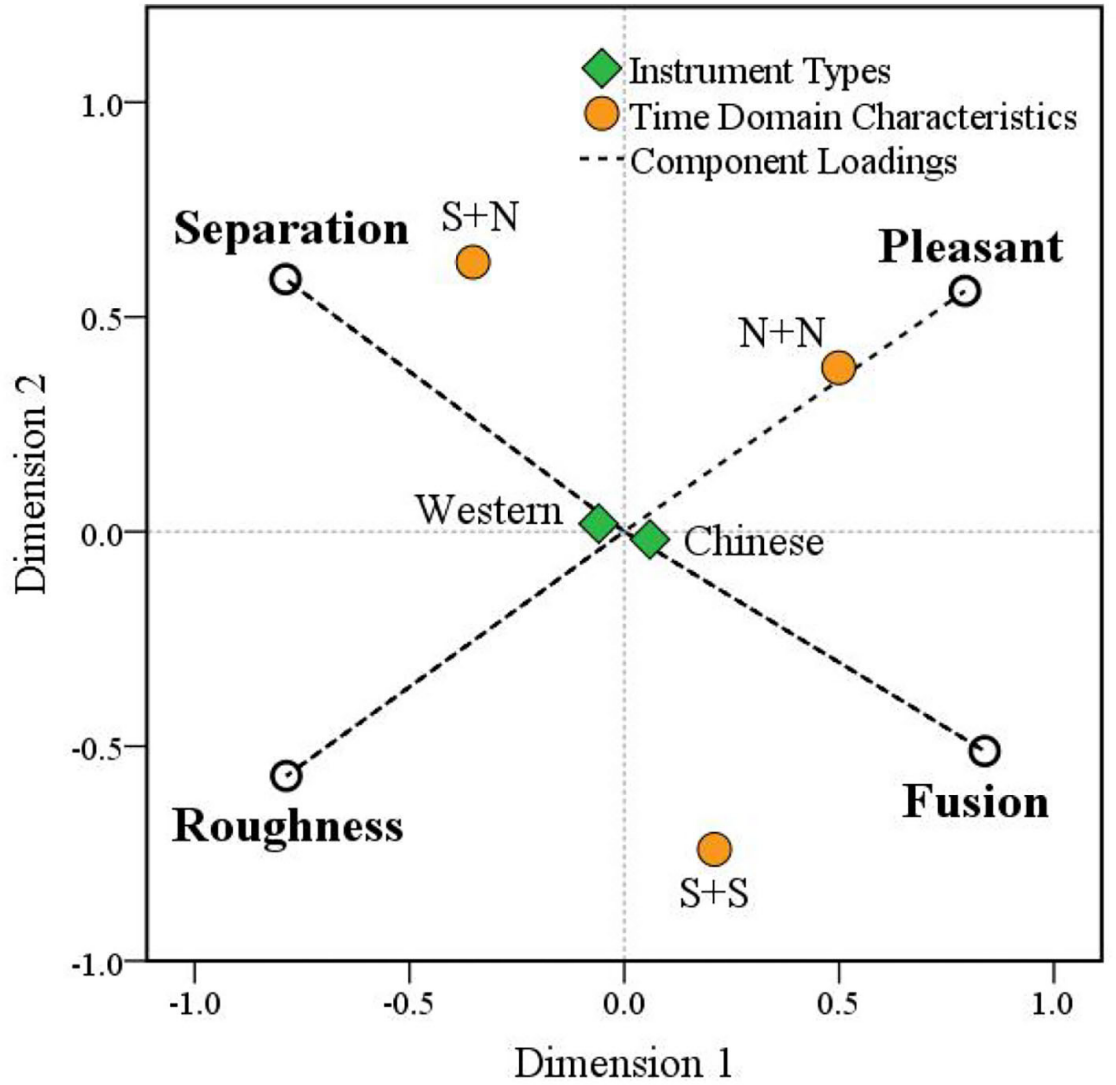
A preference space location map.

The experimental data can be statistically processed by multidimensional preference analysis. The component loadings of the four timbre perception attributes of the perception map and preference space can be obtained (Table 3, Figure 16). In the preference space, the origin represents the average level of the whole sample. Starting from the origin, the further the scatter is from the origin, the stronger its tendency is. Points falling in the same direction from the origin in roughly the same region are related to each other. Variable scatter may represent a potential factor.

**Table 3:** Component loads of timbre perception attributes in two dimensions.

	**Dimension 1**	**Dimension 2**
Fusion	0.839	−0.511
Segregation	−0.789	0.588
Roughness	−0.787	−0.569
Pleasantness	0.793	0.561

As seen from Figure 16, the four variables, which represent the fusion, segregation, roughness, and pleasantness timbre perception attributes, are distributed in the four quadrants of the preference space, which shows that these four attributes are representative for evaluating the combined timbre. In addition, fusion and segregation show opposite distributions, and roughness and pleasantness also show opposite distributions, indicating that these two pairs of attributes tend to move in opposite directions, which further verifies the results of the correlation analysis.

For the time domain characteristic factors, the timbre of the N + N type is distributed near the loading component of the pleasantness attribute, indicating that pleasantness is the main factor affecting this type of timbre. The timbre of the S + N type is distributed above the loading component of segregation, indicating that segregation is the main factor affecting this type of timbre, while the pleasantness attribute also has a slight influence on this type of timbre. The timbre of the S + S type is distributed in the middle of the fusion and roughness components, indicating that both fusion and roughness have a certain influence on the timbre of the S + S type, and the influence of fusion is slightly greater than the influence of roughness. For instrument-type factors, the scatter points of Chinese and Western instruments are very close to the origin, indicating that instrument type is not the main factor affecting timbre perception attributes. In summary, the results based on multidimensional preference analysis are consistent with the previous results of variance analysis, which further demonstrates the reliability of the conclusion.

## Construction of the Timbre Fusion Model

To explain the influencing factors of perception fusion, this paper draws on the analysis ideas of existing research and uses audio information processing methods to extract mixed audio features from time-domain waveforms and frequency spectra. Then, we attempted to establish the correlation between objective acoustic parameters and subjective perception.

### Extracting Acoustic Characteristic Parameters

Timbre is a multidimensional perception attribute that is closely related to the time-domain waveform and spectral structure of sound (Jiang et al., 2020). Objective acoustic parameters refer to any values acquired using a mathematical model, representing a normal sound signal in the time and frequency domains. To establish a timbre fusion model, an objective acoustic parameter set was constructed using 27 parameters extracted from the 518 stimuli in the timbre fusion database. These 27 parameters can be divided into 6 categories (Peeters et al., 2011):

1. *Temporal shape features:* calculated from the waveform or the signal energy envelope (e.g., attack time, temporal increase or decrease, and effective duration).
2. *Temporal features:* autocorrelation coefficients with a zero-crossing rate.
3. *Energy features:* referring to various energy contents in the signal (e.g., global energy, harmonic energy, or noise energy).
4. *Spectral shape features:* calculated from the short-time Fourier transform (STFT) of the signal (e.g., centroid, spread, skewness, kurtosis, slope, roll-off frequency, or Mel-frequency cepstral coefficients).
5. *Harmonic features:* calculated using sinusoidal harmonic modeling of the signal (e.g., the harmonic/noise ratio, the odd-to-even and tristimulus harmonic energy ratio, and harmonic deviation).
6. *Perceptual features:* calculated using a model for human hearing (e.g., relative specific loudness, sharpness, and spread).

Considering that the parameters of the audio stimuli change with time, we calculated the time-varying statistical of these parameters, including the maximum, minimum, mean, variance, standard deviation, interquartile range, skewness coefficient, and kurtosis coefficient so as to produce an objective acoustic parameter set, containing 216 parameters. We screened these parameters before establishing the regression equation. The correlation between 216 parameters and fusion was analyzed, and it was found that the correlation between mean, interquartile range, and fusion was relatively high. Therefore, the mean and interquartile range of 27 parameters were retained, and a total of 54 parameters were retained. Considering the nine parameters attack time, log attack time, decrease time, effective duration, release time, attack slope, decrease slope, frequency modulation, and amplitude modulation are mainly calculated for a single note, whereas stimuli featured a melody, these parameters were relatively unimportant, for which reason the interquartile range was omitted. This produced an objective acoustic parameter set, containing 45 parameters (see Table 4).

**Table 4:** An acoustic parameter list.

**Classification**	**Parameter name**	**Statistics**
Time domain	Temporal centroid	Mean, IQR
	Attack time	Mean
	Log attack time	Mean
	Decrease time	Mean
	Effective duration	Mean
	Release time	Mean
	Attack slope	Mean
	Decrease slope	Mean
	Frequency modulation	Mean
	Amplitude modulation	Mean
	Zero-Crossing rate	Mean, IQR
Frequency domain	Spectral centroid	Mean, IQR
	Spectral spread	Mean, IQR
	Spectral decrease	Mean, IQR
	Spectral skewness	Mean, IQR
	Spectral kurtosis	Mean, IQR
	Spectral roll-off	Mean, IQR
	Spectral-flatness measure	Mean, IQR
	Spectral crest measure	Mean, IQR
	Spectral flux	Mean, IQR
	Root mean square energy	Mean, IQR
Harmonic domain	Harmonic energy	Mean, IQR
	Noisiness energy	Mean, IQR
	Tristimulus	Mean, IQR
	Harmonic spectral deviation	Mean, IQR
	odd-to-even ratio	Mean, IQR
	Noisiness	Mean, IQR

The calculation methods of some important acoustic parameters are as follows:

1. *Zero-crossing rate:* It is defined as the number of times the audio signal waveform crossing the zero amplitude level during a 1-s interval, and it provides a rough estimator of the dominant frequency component of the signal (Alías et al., 2016).
2. *Spectrum centroid:* SC for short, defined as the centroid of spectral energy (Marchetto and Peeters, 2015). It can be defined as the first moment of the amplitude spectrum of the signal frame (the mean value of the frequency position), which represents the geometric center of the spectrum, and the unit is hertz. *f*(*n*) is the frequency after ERBfft transformation, and *P*[*E*(*n*)] is the probability value of the spectral energy of each point on the total energy. *N* is the length of the DFT transform.
  (1) $$\begin{array}{l} \text{SpecCen}\text{t}=\overset{\text{N}}{\underset{\text{n}=1}{\sum }}\text{f}\left(\text{n}\right)\text{P}\left(\text{E}\left(\text{n}\right)\right) \end{array}$$
3. *Spectrum flatness:* It is a measure of the uniformity of the power spectrum frequency distribution. It can be calculated as the ratio of the sub-band geometric average to the arithmetic average (equivalent to the MPEG-7 audio frequency spectrum flatness (ASF) description Character (Grzywczak and Gwardys, 2014).
  (2) $$\begin{array}{l} \text{SFM}\left({\text{t}}_{\text{m}}\right)=\frac{{\left(\prod_{\text{k}=1}^{\text{K}}{\text{a}}_{\text{k}}\left({\text{t}}_{\text{m}}\right)\right)}^{\frac{1}{\text{K}}}}{\frac{1}{\text{K}}\sum_{\text{k}=1}^{\text{K}}{\text{a}}_{\text{k}}\left({\text{t}}_{\text{m}}\right)} \end{array}$$
4. *Harmonic energy:* Harmonic energy is the energy of the signal explained by the harmonic partials (Sharma et al., 2020). It is obtained by summing the energy of the partials detected at a specific time. In the equation, *a*_*h*_(*t*_*m*_) is the amplitude and frequency of partial *h* at time *t*_*m*_. *H* partials are ranked by increasing frequency.
  (3) $$\begin{array}{l} {\text{E}}_{\text{H}}\left({\text{t}}_{\text{m}}\right)=\overset{\text{H}}{\underset{\text{h}=1}{\sum }}{\text{a}}_{\text{h}\;}^{2}\left({\text{t}}_{\text{m}}\right) \end{array}$$
5. *Spectral roll-off:* This parameter was proposed by Scheirer and Slaney (1997). It is defined as the frequency *f*_*c*_(*t*_*m*_) below, which 95% of the signal energy is contained, where sr/2 is the Nyquist frequency and af is the spectral amplitude at frequency *f*. In the case of harmonic sounds, it can be shown experimentally that spectral roll-off is related to the harmonic or noise cutoff frequency. The spectral roll-off also reveals an aspect of spectral shape as it is related to the brightness of a sound.
  (4) $$\begin{array}{l} \overset{{\text{f}}_{\text{c}}\left({\text{t}}_{\text{m}}\right)}{\underset{\text{f}=0}{\sum }}{\text{a}}_{\text{f}}^{2}\left({\text{t}}_{\text{m}}\right)=0.95\overset{\frac{\text{s}\text{r}}{2}}{\underset{\text{f}=0}{\sum }}{\text{a}}_{\text{f}\;}^{2}\left({\text{t}}_{\text{m}}\right) \end{array}$$

In this paper, the Timbre Toolbox (Peeters et al., 2011) and MIRtoolbox (Lartillot and Toiviainen, 2007) were used for feature extraction. The corresponding acoustic parameters were extracted from stimuli in the timbre fusion database, and the acquired data were used to construct a model of timbre fusion.

### Model Parameter Fitting

Subjective and objective correlations were adopted in the construction of the fusion model. The subjective label is the mean value of the fusion value, and the objective data are 45 dimensional objective acoustic parameters. This study uses multiple linear regressions, random forest, and multilayer perceptron to predict the subjective degree of fusion. The following is an introduction of the model and parameter settings.

(1) *Multiple linear regression:* We used multiple linear regression (Olive, 2017) to fit the data of the independent variable's 45 dimensional objective acoustic parameters and the degree of fusion of the dependent variable. The criterion of minimizing the mean square error and the gradient descent method are used to determine the linear regression coefficients. Adding Lasso regularization on the basis of standard multiple linear regression makes it easier to make the weight close to 0, which can be used for feature selection (Fonti and Belitser, 2017).

(2) *Random forest:* The random forest is composed of multiple decision trees (Pal, 2005). The root node of the decision tree is randomly selected from the training sample. The objective acoustic parameters of the sample are randomly selected by tree splitting. There is no correlation between multiple decision trees. Sklearn is used in this paper to fit the random forest model (Feurer et al., 2019). In this model, the adjustable parameters include bootstrapping, the maximum number of features for one decision tree, the maximum number of leaf nodes, and the number of decision trees.

We adjusted the parameters for the number of decision trees and the maximum number of features for one decision tree, and we adopted default values for other parameters. The increasing number of decision trees makes the model perform better, but too many trees may cause overfitting. The objective acoustic parameters in this paper are 45 attributes, and there are obvious category divisions and feature correlations between the features, so we set the maximum number of features of the decision tree as 6. The number “6” is determined by experience. If the number of features is too large, the accuracy of the model will be affected. The number of decision trees is determined according to the empirical value and the number of samples, which ranges from 9 to 11 in this paper. A total of 10 decision trees are optimally selected by testing the integration of Chinese and Western instruments and the results of the integrated model. The output result is determined jointly by each decision tree, which is the mean value of the predicted results of the test samples by the 10 decision trees.

(3) *Multilayer perceptron:* The multilayer perceptron consists of an input layer, a hidden layer, and an output layer. The layers are fully connected (Ramchoun et al., 2016). The units between the layers are connected as weight coefficients and biases, and ReLU is used as the activation function. The optimization of model training uses stochastic gradient descent (SGD) (Wu et al., 2020), and the gradient parameter update learning rate was set to 0.001.

To evaluate the accuracy of the prediction results of the model constructed by different algorithms, the goodness of fit *R*^2^ was used as the evaluation index, which is defined as follows (Brook and Arnold, 2018). The *SSR* is the regression sum of squares, *SSE* is the residual sum of squares, and *SST* is the total deviation of squares. In addition, 0 < R < 1, the closer R is to 1, the better the prediction result.


(5) $$\begin{array}{l} R^{2}=\frac{SSR}{SST}=1-\frac{SSE}{SST}=1-\frac{\sum {\left(y-ŷ\right)}^{2}}{\sum {\left(y-\bar{y}\right)}^{2}} \end{array}$$

Four-fold cross-validation was performed on 518 audio data stimuli. Each time the model was built, 3-folds were taken, and the remaining fold was used for verification. The average value of *R*^2^ was taken as the prediction accuracy of the model. The 259 pieces of Chinese and Western audio data were divided for 4-fold cross-validation, which was the same processing method as described above. The results are as follows (see Table 5).

**Table 5:** Comparison of accuracy of fusion models.

**Name**	***R^**2**^* (Chinese and Western)**	***R^**2**^* (Chinese)**	***R^**2**^* (Western)**
Linear lasso	0.414	0.541	0.305
Random forest	0.417	0.563	0.363
Multilayer perceptron	0.464	0.573	0.443

The constructed linear regression model is expressed as follows. Using this model, objective acoustic parameters with an absolute value of regression coefficient >4 are selected to characterize their contribution to fusion. In the following formula, *X* is an acoustic objective parameter.

The linear model of the fusion of Chinese musical instruments is as follows: where *F*_*Chinese*_ is the fusion degree of Chinese musical instruments.


(6) $$\begin{array}{l} F_{Chinese}=14.2X_{HarmErg}+7.7X_{SpecCent}-7.6X_{SpecFlat}\\ -6.9X_{NoiseErg}+5.6X_{ZcrRate}+4.8X_{SpecRolloff} \end{array}$$

The linear model of the fusion of Western musical instruments is as follows: where *F*_*Western*_ is the fusion degree of Western musical instruments.


(7) $$\begin{array}{l} F_{Western}=18.7X_{HarmErg}-11.6X_{NoiseErg}-10.9X_{SpecFlat}\\ -10.3X_{SpecCrest}-9.2X_{ZcrRate}-8.9X_{RMSEEnv}\\ +7.2X_{SpecSpread} \end{array}$$

The comprehensive linear model of Chinese and Western musical instruments is as follows: where *F*_*all*_ is the fusion of Chinese and Western musical instruments.


(8) $$\begin{array}{l} F_{all}=13.8X_{HarmErg}-13.6X_{SpecKurt}+13.3X_{SpecSkew}\\ -11.1X_{NoiseErg}+10.5X_{SpecCent}-10.3X_{SpecFlat}\\ -7.5X_{SpecCrest}-5.7X_{ZcrRate} \end{array}$$

The parameters in the above equations are the temporal statistical mean of the parameters. The regressors in the equations can be divided into three categories: spectral centroid, spectral roll-off, and zero crossing rate are related to the perceptual brightness and can be classified as brightness factors. Harmonic energy, noisiness energy, and RMS relate to signal energy and can be classified as energy factors. Spectral flatness, Spectral crest, Spectral spread, Spectral skewness, and Spectral kurtosis are related to an Spectral envelope, which can be classified as an Spectral envelope factor.

For Chinese instruments (Equation 6), the spectral centroid, spectral roll-off, and zero crossing rate are positively correlated with the fusion, and these three parameters are brightness factors. The brighter timbre of the dyad, the better the degree of fusion is. This is opposite to the experimental results of Sandell (1995). Sandell's experimental stimulus was western instruments, and the result was that the higher the composite centroid of the spectrum, the worse the fusion. This result shows that the perceptual fusion degree of Chinese instruments is different from that of Western instruments. This may be related to the differences in timbre between Chinese and Western instruments or to the cultural background of the subjects. Previous studies have shown that cultural background is an important factor affecting the timbre with respect to an emotional perception (Wang et al., 2021).

For Western instruments (Equation 7), the zero crossing rate is negatively correlated with the fusion, and this parameter is a brightness factor. That is, the less bright timbre of the dyad, the better the perception of fusion. This is consistent with the experimental results of Sandell (1995). This proves that the higher the composite spectral centroid of western instruments, the worse the fusion.

From the perspective of the energy factor, harmonic energy is positively correlated with fusion, while noisiness energy is negatively correlated with fusion for both Chinese and Western instruments. This shows that the more prominent the musical characteristics of the dyad, the better the perceptual fusion. The more prominent the noise characteristic is, the worse the perception fusion is. This is also one of the main reasons for the worse perception fusion of Chinese-plucked instruments. Plucking instruments produce a large number of dissonant noise components at the moment when fingernails or picks touch the strings. As a result, the fusion of the whole strumming group is poor.

In addition, the RMS of Western instruments is negatively correlated with the fusion. Although RMS is an energy factor, there is a certain relationship between the energy of an instrument's sound (playing intensity) and timbre brightness. When an instrument is played with greater force, more high-frequency components are activated, resulting in a brighter tone. That is, the higher the RMS value, the brighter the tone, the worse the perceptual fusion.

## Discussion

Through the statistical processing of experimental data, the following analysis and discussion can be made:

Sustaining and non-sustaining temporal envelopes are important factors that affect the perception attributes of timbre. Moreover, for different timbre attributes, the temporal envelopes have different effects. For the fusion and segregation attributes, the temporal properties have an impact on both Chinese and Western instruments, although these impacts are more pronounced for Chinese instruments than Western instruments. Specifically, a timbre combination with the same temporal envelope has a higher degree of fusion and a higher degree of segregation. The values of fusion from high to low are S + S > N + N > S + N (W: 5.95 > 5.67 > 3.94; C: 5.99 > 5.31 > 4.04), while the values of the degree of segregation are opposite to those of fusion: S + N > N + N > S + S (W: 6.58 > 4.84 > 4.40; C: 6.47 > 5.23 > 4.32).

For the roughness attributes, the temporal properties have an impact on roughness for both Chinese and Western instruments, although the impact is more pronounced for Western instruments than Chinese instruments. Specifically, timbre combinations with more sustaining instruments have higher roughness. The values of roughness from high to low are S + S > S + N > N + N (W: 5.12 > 4.50 > 3.18; C: 4.85 > 4.31 > 3.98). This may be because sustaining instruments contain more beating, which is an important factor that causes roughness. Similarly, for the pleasantness attributes, the temporal properties have an impact on pleasantness for both Chinese and Western instruments. However, the values of pleasantness are exactly opposite to those of roughness: N + N > S + N >S + S (W: 6.54 > 5.25 > 4.65; C: 6.16 > 5.27 > 4.63).

Moreover, through the correlation analysis and multidimensional preference analysis for the four timbre attributes, it is found that the ranking of segregation is opposite to that of fusion. The ranking of roughness is also opposite to that of pleasantness. The results further confirm the above conclusions. These results further support the conclusion of Tardieu and McAdams (2012) and Lembke et al. (2019) that fusion is reduced in the presence of non-sustaining instruments with mixed timbre. Similarly, the results of our manuscript are consistent with the conclusions drawn by Fischer et al. (2021), namely, decreasing temporal differences reduce segregation ratings. In addition, the comparison of the results of these three papers shows that the higher the similarity of timbre, the higher the fusion, and the lower the segregation, and *vice versa*. This is consistent with the conclusion of the multidimensional preference analysis in our manuscript.

From the experimental results, it can be seen that, in most cases, the instrument type (i.e., Chinese instruments or Western instruments) has less influence on the four timbre attributes. However, when the temporal envelope is N + N, the roughness will be affected by the instrument type, and the roughness of Chinese instruments is greater than that of Western instruments.

According to the variance analysis for the four timbre attributes, there is no interaction between the instrument type and the temporal envelope. That is, the difference in timbre perception attributes caused by different temporal envelopes is, basically, the same between Chinese and Western instruments.

According to the experimental data of the four timbre perception attributes, the values of fusion and segregation vary more for different temporal envelopes of Chinese instruments. However, the value of roughness varies more for different temporal envelopes when using Western instruments. That is, fusion and segregation are important attributes to evaluate the timbre combination of Chinese instruments, while roughness is an important attribute to evaluate the timbre combination of Western instruments.

Comparing the models used for analysis, the random forest and multilayer perceptron models are more effective than the linear regression models. For the model of fusion, the best accuracy is 46.4% for Chinese and Western instruments, 57.3% for Chinese instruments, and 44.3% for Western instruments. It shows that these algorithms have some limitations, and the accuracy of the model can be greatly improved. Comparing the models used for analysis, the random forest and multilayer perceptron models are more effective than the linear regression models. These two machine learning algorithms non-linearly fit the data to achieve better performance. Comparing the models of the fusion of Chinese and Western musical instruments, it can be seen that the linear regression model fits Chinese musical instrument fusion better than Western musical instruments. The model could be even more accurate. This is partly because we have a limited amount of data. However, the algorithm that we used was not state of the art enough. All of these factors have some influence on the accuracy of the model. In future research, we will attempt to further increase the amount of data and adopt deep learning algorithms to improve the accuracy of the model.

Overall, the prediction effect of the integration model for Chinese musical instruments is better than that for Western musical instruments. This difference may be related to the distribution of musical instruments. Chinese instruments are more comprehensive and evenly distributed, so the model can learn more stably and achieve effective predictions, while, in the Western fusion dataset, audio data with a high fusion degree have a larger proportion, and the model-learned features are insufficient. This result is also consistent with the distribution characteristics of the perception results from the auditory perception experiment.

Comparing the coefficients of the linear regression models of Chinese musical instruments and Western musical instruments, we can see the contributions of various objective acoustic parameters to timbre fusion. For Chinese musical instruments, the important parameters that affect fusion are harmonic energy, spectrum centroid, spectrum flatness, noise energy, zero crossing rate, and spectrum roll-off. The fusion of Western musical instruments is mainly affected by objective acoustic parameters, such as harmonic energy, noise energy, spectral flatness, spectral crest factor, zero-crossing rate, root mean square energy, and spectrum expansion.

The important objective acoustic parameters of integrated models of Chinese and Western musical instruments are harmonic energy, spectral kurtosis, spectral skewness, noise energy, spectral centroid, spectral flatness, spectral crest factor, and zero crossing rate. These parameters combine the objective acoustic parameters that have made outstanding contributions to the fusion of Chinese musical instruments and Western musical instruments. It also proves the rationality and effectiveness of the model. Comparing the models of Chinese musical instruments and Western musical instruments, their common parameters are harmonic energy, noise energy, and spectral flatness. These parameters are all related to the perceptual consonance of timbre (Wang and Meng, 2013). Therefore, we believe that objective acoustic parameters related to perceptual harmony are important factors that affect timbre fusion.

## Conclusion

In this paper, the characteristics of the timbre fusion of Chinese and Western instruments were explored, and a subjective evaluation experiment of a timbre perception based on the serial category method was designed and implemented. The effects of time domain characteristics and instrument types on fusion, segregation, roughness, and pleasantness were studied by statistical processing, which included variance analysis, multidimensional preference analysis, correlation analysis, and machine learning algorithms. The differences in the four timbre perception attributes between Chinese and Western instruments were compared. Through carrying out relevant subjective and objective experiments, the following conclusions were obtained.

Sustaining and non-sustaining time domain characteristics are important factors affecting the perception attributes of timbre. Moreover, for different timbre attributes, the time domain characteristics have different effects. According to the experimental data of the four timbre perception attributes, fusion and segregation are important attributes for evaluating the timbre combination of Chinese instruments, while roughness is an important attribute for evaluating the timbre combination of Western instruments. This conclusion further explains why the acoustic theory of symphonic orchestration is mostly based on roughness. For the study of the orchestration theory of Chinese instruments, it is necessary to explore the general rules of timbre fusion for Chinese instruments.

Multiple linear regression, random forest, and multilayer perceptron were used in this paper to construct a set of timbre fusion models for Chinese and Western instruments. The results showed that these models can better predict the timbre fusion attributes. From this research, it was also found that there are some differences between the timbre fusion models for Chinese and Western instruments, which is consistent with the analysis results of subjective experimental data. In addition, the spectrum centroid and spectrum roll-off were found to have an important influence on both the fusion model of Chinese and Western musical instruments. These parameters are all related to the brightness of the tone. Therefore, we can consider the parameter related to timbre brightness as important factors that affect the fusion of Chinese and Western instruments, although the impact is more pronounced in Chinese instruments than Western instruments. The contribution of the above parameters, especially the important parameters of the spectral centroid, was basically consistent with the results of Sandell (1991). However, there is no parameter, such as the attack time, in the regression model of the fusion degree, which was different from previous studies. This may be due to the melody content used in the fusion timbre database used in this experiment. Compared with monophonic audio data, the effect of vibration time on the entire time sequence was less obvious.

In this paper, the research on fusion is still in the exploratory stage, and this work needs to be further improved and supplemented. For example, the amount of data needed to build the database has yet to be expanded. Due to the limitation of data quantity, only a conventional algorithm was implemented in this paper to build the fusion degree model, and it is necessary to adopt deep learning to build the model on the large-scale dataset in later stages. In a follow-up study, we plan to make a special study on the timbre integration of Chinese instruments. In the aspect of database construction, a larger scale timbre fusion stimuli library should be built, and timbre fusion with different harmonies should be discussed. In addition to the timbre combinations of two instruments, the complexities of three or more timbre combinations should be considered. Future experiments should use real instrument sampling so that the research results can be extended to the practice of orchestration of Chinese and Western orchestras. From the perspective of research methods and theory, objective parameters related to timbre fusion should be further explored and analyzed, and the mathematic model should be explained from the perspective of instrument acoustics. It is also necessary to study how to apply relevant models to the orchestration practice and instrument reform of Chinese orchestral music, e.g., the development and construction of computer-aided Chinese orchestration software.

## Data Availability Statement

The raw data supporting the conclusions of this article will be made available by the authors, without undue reservation.

## Ethics Statement

The studies involving human participants were reviewed and approved by the Research Ethics Boards of the Communication University of China. The patients/participants provided their written informed consent to participate in this study.

## Author Contributions

JLi designed the study and conducted acoustic analyses. JLi, YX, SW, JJ, YJ, and JLa collected the data. JLi and YX conducted data analyses and drafted the manuscript. All authors contributed to the article and approved the submitted version.

## Funding

This work was supported by Funds for National Natural Science Foundation of China (62101514), Key Laboratory of Ministry of Culture and Tourism (WLBSYS2005), and Key Laboratory of Audio and Video Repair and Evaluation Ministry of Culture and Tourism (2021KFKT004).

## Conflict of Interest

JJ was employed by China Digital Culture Group Co., Ltd. The remaining authors declare that the research was conducted in the absence of any commercial or financial relationships that could be construed as a potential conflict of interest.

## Publisher's Note

All claims expressed in this article are solely those of the authors and do not necessarily represent those of their affiliated organizations, or those of the publisher, the editors and the reviewers. Any product that may be evaluated in this article, or claim that may be made by its manufacturer, is not guaranteed or endorsed by the publisher.

## Footnotes

^1^Website of the Vienna Symphonic Library: https://www.vsl.co.at/en/.

^2^Website of the Kong Audio Sound Library: http://www.kongaudio.com/.
