# Supplementary material for: Comparison and Analysis of Timbre Fusion for Chinese and Western Musical Instruments
Source: Front Psychol. 2022 Jul 7;13:878581. doi: 10.3389/fpsyg.2022.878581 (PMC9301319; doi:10.3389/fpsyg.2022.878581)
Supplement: Supplementary file 1 [file Data_Sheet_1.docx]

Appendix

Table 1 Instrument range list

| **No.** | **Category** | **Name** | **Register** | **No.** | **Category** | **Name** | **Register** |
| --- | --- | --- | --- | --- | --- | --- | --- |
| 1 | Western-  Wood wind | Piccolo | D5 | 27 | Chinese-  Wind | 梆笛  (Bangdi) | D5 |
| 2 |  | Flute | D4 | 28 |  | 曲笛  (Qudi) | D4 |
| 3 |  | Oboe | D4 | 29 |  | 新笛  (Xindi) | D4 |
| 4 |  | Clarinet | D3 | 30 |  | 高音笙  (Soprano Sheng) | D4 |
| 5 |  | Bassoon | D2 | 31 |  | 中音笙  (Tenor Sheng) | D3 |
| 6 |  | Saxophone | D3 | 32 |  | 高音唢呐  (Soprano Suona) | D5 |
| 7 |  | Organ | D3 | 33 |  | 中音唢呐  (Alto Suona) | D4 |
| 8 | Western-  Brass wind | Trumpet | D4 | 34 |  | 管子  (guanzi) | D4 |
| 9 |  | French horn | D3 | 35 |  | 葫芦丝  (Hulusi) | D4 |
| 10 |  | Trombone | D2 | 36 |  | 南箫  (NanXiao) | D4 |
| 11 |  | Tuba | D1 | 37 | Chinese-  Hammered/Plucked string | 扬琴  (Yangqin) | D4 |
| 12 | Western-  Bowed string | Violin | D4 | 38 |  | 柳琴  (Liuqin) | D5 |
| 13 |  | Viola | D3 | 39 |  | 琵琶  (Pipa) | D4 |
| 14 |  | Cello | D2 | 40 |  | 中阮  (Alto Ruan) | D3 |
| 15 |  | Double bass | D1 | 41 |  | 大阮  (Bass Ruan) | D2 |
| 16 | Western-  Hammered/Plucked string | Piano | D4 | 42 |  | 三弦  (Sanxian) | D3 |
| 17 |  | Harpsichord | D3 | 43 |  | 古筝  (Guzheng) | D4 |
| 18 |  | Harp | D4 | 44 |  | 古琴  (Guqin) | D2 |
| 19 |  | Guitar | D3 | 45 | Chinese-  Bowed string | 高胡  (Gaohu) | D5 |
| 20 | Western-  Percussion | Glockenspiel | D5 | 46 |  | 二胡  (Erhu) | D4 |
| 21 |  | Xylophone | D5 | 47 |  | 中胡  (Zhonghu) | D3 |
| 22 |  | Marimba | D4 | 48 |  | 革胡  (Gehu) | D2 |
| 23 |  | Vibraphone | D4 | 49 |  | 低音革胡  (Bass Gehu) | D1 |
| 24 |  | Chimes | D4 | 50 |  | 板胡  (Banhu) | D5 |
| 25 | Chinese-  Percussion | 编钟  (Bell chimes) | D5 | 51 |  | 马头琴  (Matouqin) | D3 |
| 26 |  | 编磬  (Bianqing) | D5 | 52 |  | 京胡  (Jinghu) | D5 |

Table 2 The combination of timbre experimental stimuli

N + N: nonsustaining instruments and nonsustaining instruments

S + N: sustaining instruments and nonsustaining instruments

S + S: sustaining instruments and sustaining instruments

| NO. | Type | Combination | NO. | Type | Combination |
| --- | --- | --- | --- | --- | --- |
| 1 | Western N+N | Vibraphone D4+Piano D4 | 260 | Chinese S+N | 琵琶(Pipa) D4+曲笛(Qudi) D4 |
| 2 |  | Vibraphone D4+Guitar D3 | 261 |  | 梆笛(Bangdi) D5+大阮(Bass Ruan) D2 |
| 3 |  | Vibraphone D4+Harp D4 | 262 |  | 梆笛(Bangdi) D5+琵琶(Pipa) D4 |
| 4 |  | Vibraphone D4+Harpsichord D3 | 263 |  | 梆笛(Bangdi) D5+三弦(Sanxian) D3 |
| 5 |  | Chimes D4+Piano D4 | 264 |  | 梆笛(Bangdi) D5+扬琴(Yangqin) D4 |
| 6 |  | Chimes D4+Guitar D3 | 265 |  | 柳琴(Liuqin) D5+梆笛(Bangdi) D5 |
| 7 |  | Chimes D4+Harp D4 | 266 |  | 柳琴(Liuqin) D5+曲笛(Qudi) D4 |
| 8 |  | Chimes D4+Harpsichord D3 | 267 |  | 柳琴(Liuqin) D5+新笛(Xindi) D4 |
| 9 |  | Marimba D4+Piano D4 | 268 |  | 柳琴(Liuqin) D5+中音笙(Tenor Sheng) D3 |
| 10 |  | Marimba D4+Guitar D3 | 269 |  | 柳琴(Liuqin) D5+中音唢呐(Alto Suona) D4 |
| 11 |  | Marimba D4+Harp D4 | 270 |  | 琵琶(Pipa) D4+新笛(Xindi) D4 |
| 12 |  | Marimba D4+Harpsichord D3 | 271 |  | 琵琶(Pipa) D4+中音笙(Tenor Sheng) D3 |
| 13 |  | Xylophone D5+Piano D4 | 272 |  | 琵琶(Pipa) D4+中音唢呐(Alto Suona) D4 |
| 14 |  | Xylophone D5+Guitar D3 | 273 |  | 曲笛(Qudi) D4+大阮(Bass Ruan) D2 |
| 15 |  | Xylophone D5+Harp D4 | 274 |  | 曲笛(Qudi) D4+三弦(Sanxian) D3 |
| 16 |  | Xylophone D5+Harpsichord D3 | 275 |  | 曲笛(Qudi) D4+扬琴(Yangqin) D4 |
| 17 |  | Glockenspiel D5+Piano D4 | 276 |  | 曲笛(Qudi) D4+中阮(Alto Ruan) D3 |
| 18 |  | Glockenspiel D5+Harp D4 | 277 |  | 新笛(Xindi) D4+大阮(Bass Ruan) D2 |
| 19 |  | Glockenspiel D5+Harpsichord D3 | 278 |  | 新笛(Xindi) D4+三弦(Sanxian) D3 |
| 20 |  | Glockenspiel D5+Guitar D3 | 279 |  | 新笛(Xindi) D4+中阮(Alto Ruan) D3 |
| 21 | Western S+N | Piano D4+Cello D2 | 280 |  | 扬琴(Yangqin) D4+新笛(Xindi) D4 |
| 22 |  | Piano D4+Double bass D1 | 281 |  | 扬琴(Yangqin) D4+中音笙(Tenor Sheng) D3 |
| 23 |  | Guitar D3+Cello D2 | 282 |  | 扬琴(Yangqin) D4+中音唢呐(Alto Suona) D4 |
| 24 |  | Guitar D3+Double bass D1 | 283 |  | 中音笙(Tenor Sheng) D3+大阮(Bass Ruan) D2 |
| 25 |  | Guitar D3+Viola D3 | 284 |  | 中音笙(Tenor Sheng) D3+三弦(Sanxian) D3 |
| 26 |  | Harp D4+Cello D2 | 285 |  | 中音唢呐(Alto Suona) D4+大阮(Bass Ruan) D2 |
| 27 |  | Harp D4+Double bass D1 | 286 |  | 中音唢呐(Alto Suona) D4+三弦(Sanxian) D3 |
| 28 |  | Harp D4+Viola D3 | 287 |  | 中音唢呐(Alto Suona) D4+中阮(Alto Ruan) D3 |
| 29 |  | Violin D4+Piano D4 | 288 |  | 中音笙(Tenor Sheng) D3+中阮(Alto Ruan) D3 |
| 30 |  | Violin D4+Guitar D3 | 289 |  | 梆笛(Bangdi) D5+中阮(Alto Ruan) D3 |
| 31 |  | Violin D4+Harp D4 | 290 |  | 高音笙(Soprano Sheng) D4+大阮(Bass Ruan) D2 |
| 32 |  | Violin D4+Harpsichord D3 | 291 |  | 高音笙(Soprano Sheng) D4+琵琶(Pipa) D4 |
| 33 |  | Harpsichord D3+Cello D2 | 292 |  | 高音笙(Soprano Sheng) D4+三弦(Sanxian) D3 |
| 34 |  | Harpsichord D3+Double bass D1 | 293 |  | 高音笙(Soprano Sheng) D4+扬琴(Yangqin) D4 |
| 35 |  | Harpsichord D3+Viola D3 | 294 |  | 高音笙(Soprano Sheng) D4+中阮(Alto Ruan) D3 |
| 36 |  | Piano D4+Viola D3 | 295 |  | 高音唢呐(Soprano Suona) D5+大阮(Bass Ruan) D2 |
| 37 | Western S+N | Chimes D4+Cello D2 | 296 |  | 高音唢呐(Soprano Suona) D5+柳琴(Liuqin) D5 |
| 38 |  | Chimes D4+Double bass D1 | 297 |  | 高音唢呐(Soprano Suona) D5+琵琶(Pipa) D4 |
| 39 |  | Chimes D4+Violin D4 | 298 |  | 高音唢呐(Soprano Suona) D5+三弦(Sanxian) D3 |
| 40 |  | Chimes D4+Viola D3 | 299 |  | 高音唢呐(Soprano Suona) D5+扬琴(Yangqin) D4 |
| 41 |  | Marimba D4+Cello D2 | 300 |  | 高音唢呐(Soprano Suona) D5+中阮(Alto Ruan) D3 |
| 42 |  | Marimba D4+Double bass D1 | 301 |  | 管子(guanzi) D4+琵琶(Pipa) D4 |
| 43 |  | Marimba D4+Violin D4 | 302 |  | 管子(guanzi) D4+三弦(Sanxian) D3 |
| 44 |  | Marimba D4+Viola D3 | 303 |  | 管子(guanzi) D4+扬琴(Yangqin) D4 |
| 45 |  | Xylophone D5+Cello D2 | 304 |  | 管子(guanzi) D4+中阮(Alto Ruan) D3 |
| 46 |  | Xylophone D5+Double bass D1 | 305 |  | 管子(guanzi) D4+中阮(Alto Ruan) D3 |
| 47 |  | Xylophone D5+Violin D4 | 306 |  | 柳琴(Liuqin) D5+高音笙(Soprano Sheng) D4 |
| 48 |  | Xylophone D5+Viola D3 | 307 |  | 柳琴(Liuqin) D5+管子(guanzi) D4 |
| 49 |  | Glockenspiel D5+Cello D2 | 308 |  | 南萧(NanXiao) D4+古琴(Guzheng) D2 |
| 50 |  | Glockenspiel D5+Double bass D1 | 309 | Chinese S+S | 板胡(Banhu) D5+梆笛(Bangdi) D5 |
| 51 |  | Glockenspiel D5+Viola D3 | 310 |  | 板胡(Banhu) D5+曲笛(Qudi) D4 |
| 52 |  | Vibraphone D4+Cello D2 | 311 |  | 板胡(Banhu) D5+新笛(Xindi) D4 |
| 53 |  | Vibraphone D4+Double bass D1 | 312 |  | 板胡(Banhu) D5+中音笙(Tenor Sheng) D3 |
| 54 |  | Vibraphone D4+Violin D4 | 313 |  | 梆笛(Bangdi) D5+低音革胡(Bass Gehu) D1 |
| 55 |  | Vibraphone D4+Viola D3 | 314 |  | 梆笛(Bangdi) D5+二胡(Erhu) D4 |
| 56 |  | Glockenspiel D5+Violin D4 | 315 |  | 梆笛(Bangdi) D5+高胡(Gaohu) D4 |
| 57 | Western S+N | Clarinet D3+Guitar D3 | 316 |  | 梆笛(Bangdi) D5+革胡(Gehu) D2 |
| 58 |  | Clarinet D3+Harpsichord D3 | 317 |  | 梆笛(Bangdi) D5+马头琴(Matouqin) D3 |
| 59 |  | Piccolo D5+Piano D4 | 318 |  | 梆笛(Bangdi) D5+中胡(Zhonghu) D3 |
| 60 |  | Piccolo D5+Guitar D4 | 319 |  | 二胡(Erhu) D4+曲笛(Qudi) D4 |
| 61 |  | Piccolo D5+Harp D4 | 320 |  | 二胡(Erhu) D4+新笛(Xindi) D4 |
| 62 |  | Piccolo D5+Harpsichord D4 | 321 |  | 二胡(Erhu) D4+中音笙(Tenor Sheng) D3 |
| 63 |  | Piano D3+Bassoon D2 | 322 |  | 二胡(Erhu) D4+中音唢呐(Alto Suona) D4 |
| 64 |  | Piano D4+Clarinet D3 | 323 |  | 高胡(Gaohu) D4+曲笛(Qudi) D4 |
| 65 |  | Piano D4+saxophone D3 | 324 |  | 高胡(Gaohu) D4+新笛(Xindi) D4 |
| 66 |  | Guitar D3+Bassoon D2 | 325 |  | 高胡(Gaohu) D4+中音笙(Tenor Sheng) D3 |
| 67 |  | saxophone D3+Guitar D3 | 326 |  | 高胡(Gaohu) D4+中音唢呐(Alto Suona) D4 |
| 68 |  | saxophone D3+Harpsichord D3 | 327 |  | 马头琴(Matouqin) D3+中音笙(Tenor Sheng) D3 |
| 69 |  | Harp 4+saxophone D3 | 328 |  | 曲笛(Qudi) D4+低音革胡(Bass Gehu) D1 |
| 70 |  | Harp D3+Bassoon D2 | 329 |  | 曲笛(Qudi) D4+革胡(Gehu) D2 |
| 71 |  | Oboe D4+Piano D4 | 330 |  | 曲笛(Qudi) D4+中胡(Zhonghu) D3 |
| 72 |  | Oboe D4+Guitar D3 | 331 |  | 新笛(Xindi) D4+低音革胡(Bass Gehu) D1 |
| 73 |  | Oboe D4+Harp D4 | 332 |  | 新笛(Xindi) D4+革胡(Gehu) D2 |
| 74 |  | Oboe D4+Harpsichord D3 | 333 |  | 新笛(Xindi) D4+马头琴(Matouqin) D3 |
| 75 |  | Harpsichord D3+Bassoon D2 | 334 |  | 新笛(Xindi) D4+中胡(Zhonghu) D3 |
| 76 |  | Flute D4+Piano D4 | 335 |  | 中音笙(Tenor Sheng) D3+低音革胡(Bass Gehu) D1 |
| 77 |  | Flute D4+Guitar D3 | 336 |  | 中音笙(Tenor Sheng) D3+革胡(Gehu) D2 |
| 78 |  | Flute D4+Harp D4 | 337 |  | 中音笙(Tenor Sheng) D3+中胡(Zhonghu) D3 |
| 79 |  | Flute D4+Harpsichord D3 | 338 |  | 中音唢呐(Alto Suona) D4+低音革胡(Bass Gehu) D1 |
| 80 |  | Harp D4+Clarinet D3 | 339 |  | 中音唢呐(Alto Suona) D4+革胡(Gehu) D2 |
| 81 | Western S+N | Vibraphone D4+Bassoon D2 | 340 |  | 中音唢呐(Alto Suona) D4+马头琴(Matouqin) D3 |
| 82 |  | Vibraphone D4+Clarinet D3 | 341 |  | 中音唢呐(Alto Suona) D4+中胡(Zhonghu) D3 |
| 83 |  | Vibraphone D4+saxophone D3 | 342 |  | 板胡(Banhu) D5+中音唢呐(Alto Suona) D4 |
| 84 |  | Vibraphone D4+Oboe D4 | 343 |  | 曲笛(Qudi) D4+马头琴(Matouqin) D3 |
| 85 |  | Piccolo D5+Vibraphone D4 | 344 |  | 高音笙(Soprano Sheng) D4+马头琴(Matouqin) D3 |
| 86 |  | Piccolo D5+Chimes D4 | 345 |  | 高音唢呐(Soprano Suona) D5+马头琴(Matouqin) D3 |
| 87 |  | Piccolo D5+Marimba D4 | 346 |  | 管子(guanzi) D4+马头琴(Matouqin) D3 |
| 88 |  | Piccolo D5+Xylophone D5 | 347 |  | 板胡(Banhu) D5+高音笙(Soprano Sheng) D4 |
| 89 |  | Piccolo D5+Glockenspiel D5 | 348 |  | 板胡(Banhu) D5+高音唢呐(Soprano Suona) D5 |
| 90 |  | Chimes D4+Bassoon D2 | 349 |  | 板胡(Banhu) D5+管子(guanzi) D4 |
| 91 |  | Chimes D4+Clarinet D3 | 350 |  | 高胡(Gaohu) D4+高音笙(Soprano Sheng) D4 |
| 92 |  | Chimes D4+saxophone D3 | 351 |  | 高胡(Gaohu) D4+高音唢呐(Soprano Suona) D5 |
| 93 |  | Chimes D4+Oboe D4 | 352 |  | 高胡(Gaohu) D4+管子(guanzi) D4 |
| 94 |  | Marimba D4+Bassoon D2 | 353 |  | 高音笙(Soprano Sheng) D4+低音革胡(Gehu) D2 |
| 95 |  | Marimba D4+Clarinet D3 | 354 |  | 高音笙(Soprano Sheng) D4+二胡(Erhu) D4 |
| 96 |  | Marimba D4+saxophone D3 | 355 |  | 高音笙(Soprano Sheng) D4+革胡(Gehu) D2 |
| 97 |  | Marimba D4+Oboe D4 | 356 |  | 高音笙(Soprano Sheng) D4+中胡(Zhonghu) D3 |
| 98 |  | Xylophone D5+Bassoon D2 | 357 |  | 高音唢呐(Soprano Suona) D5+低音革胡(Bass Gehu) D1 |
| 99 |  | Xylophone D5+Clarinet D3 | 358 |  | 高音唢呐(Soprano Suona) D5+二胡(Erhu) D4 |
| 100 |  | Xylophone D5+saxophone D3 | 359 |  | 高音唢呐(Soprano Suona) D5+革胡(Gehu) D2 |
| 101 |  | Xylophone D5+Oboe D4 | 360 |  | 高音唢呐(Soprano Suona) D5+管子(guanzi) D4 |
| 102 |  | Xylophone D5+Flute D4 | 361 |  | 管子(guanzi) D4+低音革胡(Gehu) D2 |
| 103 |  | Flute D4+Vibraphone D4 | 362 |  | 管子(guanzi) D4+二胡(Erhu) D4 |
| 104 |  | Flute D4+Chimes D4 | 363 |  | 管子(guanzi) D4+革胡(Gehu) D2 |
| 105 |  | Flute D4+Marimba D4 | 364 |  | 管子(guanzi) D4+中胡(Zhonghu) D3 |
| 106 |  | Glockenspiel D5+Bassoon D2 | 365 | Chinese N+N | 编磬(Bianqing) D5+南萧(NanXiao) D4 |
| 107 |  | Glockenspiel D5+Clarinet D3 | 366 |  | 编钟(Bell chimes) D5+南萧(NanXiao) D4 |
| 108 |  | Glockenspiel D5+saxophone D3 | 367 |  | 编钟(Bell chimes) D5+中音笙(Tenor Sheng) D3 |
| 109 |  | Glockenspiel D5+Oboe D4 | 368 |  | 编磬(Bianqing) D5+高音笙(Soprano Sheng) D4 |
| 110 |  | Glockenspiel D5+Flute D4 | 369 |  | 编磬(Bianqing) D5+曲笛(Qudi) D4 |
| 111 | Western S+S | Bassoon D2+Cello D2 | 370 |  | 编磬(Bianqing) D5+中阮(Alto Ruan) D3 |
| 112 |  | Bassoon D2+Double bass D1 | 371 |  | 编磬(Bianqing) D5+中音笙(Tenor Sheng) D3 |
| 113 |  | Clarinet D3+Cello D2 | 372 |  | 编钟(Bell chimes) D5+高音笙(Soprano Sheng) D4 |
| 114 |  | Clarinet D3+Double bass D1 | 373 |  | 编钟(Bell chimes) D5+曲笛(Qudi) D4 |
| 115 |  | Clarinet D3+Viola D3 | 374 |  | 编钟(Bell chimes) D5+中阮(Alto Ruan) D3 |
| 116 |  | Piccolo D5+Cello D2 | 375 | Chinese S+N | 板胡(Banhu) D5+大阮(Bass Ruan) D2 |
| 117 |  | Piccolo D5+Double bass D1 | 376 |  | 板胡(Banhu) D5+琵琶(Pipa) D4 |
| 118 |  | Piccolo D5+Violin D4 | 377 |  | 板胡(Banhu) D5+三弦(Sanxian) D3 |
| 119 |  | Piccolo D5+Viola D3 | 378 |  | 板胡(Banhu) D5+中阮(Alto Ruan) D3 |
| 120 |  | saxophone D3+Cello D2 | 379 |  | 大阮(Bass Ruan) D2+低音革胡(Bass Gehu) D1 |
| 121 |  | saxophone D3+Double bass D1 | 380 |  | 大阮(Bass Ruan) D2+革胡(Gehu) D2 |
| 122 |  | saxophone D3+Viola D3 | 381 |  | 二胡(Erhu) D4+大阮(Bass Ruan) D2 |
| 123 |  | Oboe D4+Cello D2 | 382 |  | 二胡(Erhu) D4+琵琶(Pipa) D4 |
| 124 |  | Oboe D4+Double bass D1 | 383 |  | 二胡(Erhu) D4+三弦(Sanxian) D3 |
| 125 |  | Oboe D4+Violin D4 | 384 |  | 二胡(Erhu) D4+扬琴(Yangqin) D4 |
| 126 |  | Oboe D4+Viola D3 | 385 |  | 二胡(Erhu) D4+中阮(Alto Ruan) D3 |
| 127 |  | Violin D4+Bassoon D2 | 386 |  | 高胡(Gaohu) D4+大阮(Bass Ruan) D2 |
| 128 |  | Violin D4+Clarinet D3 | 387 |  | 高胡(Gaohu) D4+琵琶(Pipa) D4 |
| 129 |  | Violin D4+saxophone D3 | 388 |  | 高胡(Gaohu) D4+三弦(Sanxian) D3 |
| 130 |  | Flute D4+Cello D2 | 389 |  | 高胡(Gaohu) D4+扬琴(Yangqin) D4 |
| 131 |  | Flute D4+Double bass D1 | 390 | Chinese S+N | 高胡(Gaohu) D4+中阮(Alto Ruan) D3 |
| 132 |  | Flute D4+Violin D4 | 391 |  | 柳琴(Liuqin) D5+板胡(Banhu) D5 |
| 133 | Western S+S | Flute D4+Viola D3 | 392 |  | 柳琴(Liuqin) D5+低音革胡(Bass Gehu) D1 |
| 134 |  | Viola D3+Bassoon D2 | 393 |  | 柳琴(Liuqin) D5+二胡(Erhu) D4 |
| 135 | Western S+S | Bassoon D2+Tuba D1 | 394 |  | 柳琴(Liuqin) D5+高胡(Gaohu) D4 |
| 136 |  | Bassoon D2+Trumpet D4 | 395 |  | 柳琴(Liuqin) D5+革胡(Gehu) D2 |
| 137 |  | Bassoon D2+French horn D3 | 396 |  | 柳琴(Liuqin) D5+马头琴(Matouqin) D3 |
| 138 |  | Bassoon D2+Trombone D2 | 397 |  | 柳琴(Liuqin) D5+中胡(Zhonghu) D3 |
| 139 |  | Clarinet D3+Tuba D1 | 398 |  | 马头琴(Matouqin) D3+大阮(Bass Ruan) D2 |
| 140 |  | Clarinet D3+French horn D3 | 399 |  | 琵琶(Pipa) D4+革胡(Gehu) D2 |
| 141 |  | Clarinet D3+Trombone D2 | 400 |  | 琵琶(Pipa) D4+马头琴(Matouqin) D3 |
| 142 |  | Piccolo D5+Tuba D1 | 401 |  | 琵琶(Pipa) D4+中胡(Zhonghu) D3 |
| 143 |  | Piccolo D5+Trumpet D4 | 402 |  | 三弦(Sanxian) D3+低音革胡(Bass Gehu) D1 |
| 144 |  | Piccolo D5+French horn D3 | 403 |  | 三弦(Sanxian) D3+革胡(Gehu) D2 |
| 145 |  | Piccolo D5+Trombone D2 | 404 |  | 三弦(Sanxian) D3+马头琴(Matouqin) D3 |
| 146 |  | saxophone D3+Tuba D1 | 405 |  | 三弦(Sanxian) D3+中胡(Zhonghu) D3 |
| 147 |  | saxophone D3+French horn D3 | 406 |  | 扬琴(Yangqin) D4+低音革胡(Bass Gehu) D1 |
| 148 |  | saxophone D3+Trombone D2 | 407 |  | 扬琴(Yangqin) D4+革胡(Gehu) D2 |
| 149 |  | Oboe D4+Tuba D1 | 408 |  | 扬琴(Yangqin) D4+马头琴(Matouqin) D3 |
| 150 |  | Oboe D4+Trumpet D4 | 409 |  | 扬琴(Yangqin) D4+中胡(Zhonghu) D3 |
| 151 |  | Oboe D4+French horn D3 | 410 |  | 中胡(Zhonghu) D3+大阮(Bass Ruan) D2 |
| 152 |  | Oboe D4+Trombone D2 | 411 |  | 中胡(Zhonghu) D3+中阮(Alto Ruan) D3 |
| 153 |  | Trumpet D4+Clarinet D3 | 412 |  | 中阮(Alto Ruan) D3+低音革胡(Bass Gehu) D1 |
| 154 |  | Trumpet D4+saxophone D3 | 413 |  | 中阮(Alto Ruan) D3+革胡(Gehu) D2 |
| 155 |  | Flute D4+Tuba D1 | 414 |  | 中阮(Alto Ruan) D3+马头琴(Matouqin) D3 |
| 156 |  | Flute D4+Trumpet D4 | 415 |  | 板胡(Banhu) D5+扬琴(Yangqin) D4 |
| 157 |  | Flute D4+French horn D3 | 416 |  | 琵琶(Pipa) D4+低音革胡(Bass Gehu) D1 |
| 158 |  | Flute D4+Trombone D2 | 417 | Chinese S+S | 高音笙(Soprano Sheng) D4+南萧(NanXiao) D4 |
| 159 | Western S+N | Piano D4+Tuba D1 | 418 |  | 梆笛(Bangdi) D5+葫芦丝(Hulusi) D4 |
| 160 |  | Piano D4+Trumpet D4 | 419 |  | 梆笛(Bangdi) D5+南萧(NanXiao) D4 |
| 161 |  | Piano D4+French horn D3 | 420 |  | 梆笛(Bangdi) D5+曲笛(Qudi) D4 |
| 162 |  | Piano D4+Trombone D2 | 421 |  | 梆笛(Bangdi) D5+新笛(Xindi) D4 |
| 163 |  | Harp D4+Tuba D1 | 422 |  | 梆笛(Bangdi) D5+中音笙(Tenor Sheng) D3 |
| 164 |  | Harp D4+Trumpet D4 | 423 |  | 梆笛(Bangdi) D5+中音唢呐(Alto Suona) D4 |
| 165 |  | Harp D4+French horn D3 | 424 |  | 高音笙(Soprano Sheng) D4+葫芦丝(Hulusi) D4 |
| 166 |  | Harp D4+Trombone D2 | 425 |  | 高音笙(Soprano Sheng) D4+曲笛(Qudi) D4 |
| 167 |  | Trumpet D4+Harpsichord D3 | 426 |  | 高音笙(Soprano Sheng) D4+新笛(Xindi) D4. |
| 168 |  | Harpsichord D3+Tuba D1 | 427 |  | 高音笙(Soprano Sheng) D4+中音唢呐(Alto Suona) D4 |
| 169 |  | Harpsichord D3+French horn D3 | 428 |  | 高音唢呐(Soprano Suona) D5+中音唢呐(Alto Suona) D4 |
| 170 |  | Harpsichord D3+Trombone D2 | 429 |  | 高音唢呐(Soprano Suona) D5+葫芦丝(Hulusi) D4 |
| 171 |  | Guitar D3+Tuba D1 | 430 |  | 高音唢呐(Soprano Suona) D5+南萧(NanXiao) D4 |
| 172 |  | Guitar D3+French horn D3 | 431 |  | 高音唢呐(Soprano Suona) D5+曲笛(Qudi) D4 |
| 173 |  | Guitar D3+Trombone D2 | 432 |  | 高音唢呐(Soprano Suona) D5+新笛(Xindi) D4 |
| 174 |  | Trumpet D4+Guitar D3 | 433 |  | 管子(guanzi) D4+葫芦丝(Hulusi) D4 |
| 175 | Western S+N | Vibraphone D4+Tuba D1 | 434 |  | 管子(guanzi) D4+南萧(NanXiao) D4 |
| 176 |  | Vibraphone D4+Trumpet D4 | 435 |  | 管子(guanzi) D4+曲笛(Qudi) D4 |
| 177 |  | Vibraphone D4+French horn D3 | 436 |  | 管子(guanzi) D4+新笛(Xindi) D4 |
| 178 |  | Vibraphone D4+Trombone D2 | 437 |  | 管子(guanzi) D4+中音笙(Tenor Sheng) D3 |
| 179 |  | Chimes D4+Tuba D1 | 438 |  | 管子(guanzi) D4+中音唢呐(Alto Suona) D4 |
| 180 |  | Chimes D4+Trumpet D4 | 439 |  | 葫芦丝(Hulusi) D4+南萧(NanXiao) D4 |
| 181 |  | Chimes D4+French horn D3 | 440 |  | 葫芦丝(Hulusi) D4+曲笛(Qudi) D4 |
| 182 |  | Chimes D4+Trombone D2 | 441 |  | 葫芦丝(Hulusi) D4+新笛(Xindi) D4 |
| 183 |  | Marimba D4+Tuba D1 | 442 |  | 葫芦丝(Hulusi) D4+中音笙(Tenor Sheng) D3 |
| 184 |  | Marimba D4+Trumpet D4 | 443 |  | 南萧(NanXiao) D4+曲笛(Qudi) D4 |
| 185 |  | Marimba D4+French horn D3 | 444 |  | 南萧(NanXiao) D4+新笛(Xindi) D4 |
| 186 |  | Marimba D4+Trombone D2 | 445 |  | 南萧(NanXiao) D4+中音笙(Tenor Sheng) D3 |
| 187 |  | Xylophone D5+Tuba D1 | 446 |  | 曲笛(Qudi) D4+新笛(Xindi) D4 |
| 188 |  | Xylophone D5+Trumpet D4 | 447 |  | 曲笛(Qudi) D4+中音笙(Tenor Sheng) D3 |
| 189 |  | Xylophone D5+French horn D3 | 448 |  | 新笛(Xindi) D4+中音笙(Tenor Sheng) D3 |
| 190 |  | Xylophone D5+Trombone D2 | 449 |  | 中音唢呐(Alto Suona) D4+葫芦丝(Hulusi) D4 |
| 191 |  | Glockenspiel D5+Tuba D1 | 450 |  | 中音唢呐(Alto Suona) D4+南萧(NanXiao) D4 |
| 192 |  | Glockenspiel D5+Trumpet D4 | 451 |  | 中音唢呐(Alto Suona) D4+曲笛(Qudi) D4 |
| 193 |  | Glockenspiel D5+French horn D3 | 452 |  | 中音唢呐(Alto Suona) D4+新笛(Xindi) D4 |
| 194 |  | Glockenspiel D5+Trombone D2 | 453 |  | 中音唢呐(Alto Suona) D4+中音笙(Tenor Sheng) D3 |
| 195 | Western S+S | Tuba D1+Double bass D1.wav | 454 |  | 高音笙(Soprano Sheng) D4+梆笛(Bangdi) D5 |
| 196 |  | Cello D2+Tuba D1 | 455 |  | 高音笙(Soprano Sheng) D4+管子(guanzi) D4 |
| 197 |  | Cello D2+Trombone D2 | 456 |  | 高音笙(Soprano Sheng) D4+中音笙(Tenor Sheng) D3 |
| 198 |  | Trumpet D4+Cello D2 | 457 |  | 高音唢呐(Soprano Suona) D5+梆笛(Bangdi) D5 |
| 199 |  | Trumpet D4+Double bass D1 | 458 |  | 高音唢呐(Soprano Suona) D5+高音笙(Soprano Sheng) D4 |
| 200 |  | Trumpet D4+Viola D3 | 459 |  | 高音唢呐(Soprano Suona) D5+管子(guanzi) D4 |
| 201 |  | Violin D4+Tuba D1 | 460 |  | 高音唢呐(Soprano Suona) D5+中音笙(Tenor Sheng) D3 |
| 202 |  | Violin D4+Trumpet D4 | 461 |  | 管子(guanzi) D4+梆笛(Bangdi) D5 |
| 203 |  | Violin D4+French horn D3 | 462 | Chinese N+N | 编磬(Bianqing) D5+编钟(Bell chimes) D5 |
| 204 |  | Violin D4+Trombone D2 | 463 |  | 柳琴(Liuqin) D5+三弦(Sanxian) D3 |
| 205 |  | French horn D3+Cello D2 | 464 |  | 大阮(Bass Ruan) D2+古琴(Guzheng) D2 |
| 206 |  | French horn D3+Double bass D1 | 465 |  | 古筝(Guzheng) D4+大阮(Bass Ruan) D2 |
| 207 |  | Trombone D2+Double bass D1 | 466 |  | 古筝(Guzheng) D4+古琴(Guzheng) D2 |
| 208 |  | Viola D3+Tuba D1 | 467 |  | 古筝(Guzheng) D4+琵琶(Pipa) D4 |
| 209 |  | Viola D3+French horn D3 | 468 |  | 古筝(Guzheng) D4+三弦(Sanxian) D3. |
| 210 |  | Viola D3+Trombone D2 | 469 |  | 古筝(Guzheng) D4+扬琴(Yangqin) D4 |
| 211 | Western N+N | Xylophone D5+Marimba D4 | 470 |  | 古筝(Guzheng) D4+中阮(Alto Ruan) D3 |
| 212 |  | Vibraphone D4+Chimes D4 | 471 |  | 柳琴(Liuqin) D5+大阮(Bass Ruan) D2 |
| 213 |  | Vibraphone D4+Marimba D4 | 472 |  | 柳琴(Liuqin) D5+古琴(Guzheng) D2 |
| 214 |  | Chimes D4+Marimba D4 | 473 |  | 柳琴(Liuqin) D5+古筝(Guzheng) D4 |
| 215 |  | Chimes D4+Xylophone D5 | 474 |  | 柳琴(Liuqin) D5+琵琶(Pipa) D4 |
| 216 |  | Xylophone D5+Vibraphone D4 | 475 |  | 柳琴(Liuqin) D5+扬琴(Yangqin) D4 |
| 217 |  | Xylophone D5+Glockenspiel D5 | 476 |  | 柳琴(Liuqin) D5+中阮(Alto Ruan) D3 |
| 218 |  | Glockenspiel D5+Vibraphone D4 | 477 |  | 琵琶(Pipa) D4+大阮(Bass Ruan) D2 |
| 219 |  | Glockenspiel D5+Chimes D4 | 478 |  | 琵琶(Pipa) D4+古琴(Guzheng) D2 |
| 220 |  | Glockenspiel D5+Marimba D4 | 479 |  | 琵琶(Pipa) D4+三弦(Sanxian) D3 |
| 221 | Western S+S | Cello D2+Double bass D1 | 480 |  | 琵琶(Pipa) D4+中阮(Alto Ruan) D3 |
| 222 |  | Violin D4+Cello D2 | 481 |  | 三弦(Sanxian) D3+大阮(Bass Ruan) D2 |
| 223 |  | Violin D4+Double bass D1 | 482 |  | 三弦(Sanxian) D3+古琴(Guzheng) D2 |
| 224 |  | Violin D4+Viola D3 | 483 |  | 三弦(Sanxian) D3+中阮(Alto Ruan) D3 |
| 225 |  | Viola D3+Cello D2 | 484 |  | 扬琴(Yangqin) D4+大阮(Bass Ruan) D2 |
| 226 |  | Viola D3+Double bass D1 | 485 |  | 扬琴(Yangqin) D4+古琴(Guzheng) D2 |
| 227 | Western N+N | Piano D4+Guitar D3 | 486 |  | 扬琴(Yangqin) D4+琵琶(Pipa) D4 |
| 228 |  | Piano D4+Harp D4 | 487 |  | 扬琴(Yangqin) D4+三弦(Sanxian) D3 |
| 229 |  | Piano D4+Harpsichord D3 | 488 |  | 扬琴(Yangqin) D4+中阮(Alto Ruan) D3 |
| 230 |  | Harp D4+Guitar D3 | 489 |  | 中阮(Alto Ruan) D3+大阮(Bass Ruan) D2 |
| 231 |  | Harp D4+Harpsichord D3 | 490 |  | 中阮(Alto Ruan) D3+古琴(Guzheng) D2 |
| 232 |  | Harpsichord D3+Guitar D3 | 491 | Chinese S+S | 板胡(Banhu) D5+低音革胡(Bass Gehu) D1 |
| 233 | Western S+S | Clarinet D3+Bassoon D2 | 492 |  | 板胡(Banhu) D5+二胡(Erhu) D4 |
| 234 |  | Clarinet D3+saxophone D3 | 493 |  | 二胡(Erhu) D4+革胡(Gehu) D2 |
| 235 |  | Piccolo D5+Bassoon D2 | 494 |  | 高胡(Gaohu) D4+革胡(Gehu) D2 |
| 236 |  | Piccolo D5+Clarinet D3 | 495 |  | 革胡(Gehu) D2+板胡(Banhu) D5 |
| 237 |  | Piccolo D5+Organ D3 | 496 |  | 革胡(Gehu) D2+低音革胡(Bass Gehu) D1 |
| 238 |  | Piccolo D5+saxophone D3 | 497 |  | 京胡(Jinghu) D5+低音革胡(Bass Gehu) D1 |
| 239 |  | Piccolo D5+Oboe D4 | 498 |  | 京胡(Jinghu) D5+二胡(Erhu) D4 |
| 240 |  | Piccolo D5+Flute D4 | 499 |  | 京胡(Jinghu) D5+高胡(Gaohu) D4 |
| 241 |  | Organ D3+Bassoon D2 | 500 |  | 京胡(Jinghu) D5+革胡(Gehu) D2 |
| 242 |  | Organ D3+Clarinet D3 | 501 |  | 京胡(Jinghu) D5+马头琴(Matouqin) D3 |
| 243 |  | Organ D3+saxophone D3 | 502 |  | 京胡(Jinghu) D5+中胡(Zhonghu) D3 |
| 244 |  | saxophone D3+Bassoon D2 | 503 |  | 马头琴(Matouqin) D3+低音革胡(Bass Gehu) D1 |
| 245 |  | Oboe D4+Bassoon D2 | 504 |  | 马头琴(Matouqin) D3+革胡(Gehu) D2 |
| 246 |  | Oboe D4+Organ D3 | 505 |  | 中胡(Zhonghu) D3+低音革胡(Bass Gehu) D1 |
| 247 |  | Oboe D4+saxophone D3 | 506 |  | 中胡(Zhonghu) D3+革胡(Gehu) D2 |
| 248 |  | Oboe D4+Flute D4 | 507 |  | 中胡(Zhonghu) D3+马头琴(Matouqin) D3 |
| 249 |  | Flute D4+Bassoon D2 | 508 |  | 板胡(Banhu) D5+高胡(Gaohu) D4 |
| 250 |  | Flute D4+Clarinet D3 | 509 |  | 板胡(Banhu) D5+京胡(Jinghu) D5 |
| 251 |  | Flute D4+Organ D3 | 510 |  | 板胡(Banhu) D5+马头琴(Matouqin) D3 |
| 252 |  | Flute D4+saxophone D3 | 511 |  | 板胡(Banhu) D5+中胡(Zhonghu) D3 |
| 253 |  | Oboe D4+Clarinet D3 | 512 |  | 二胡(Erhu) D4+中胡(Zhonghu) D3 |
| 254 | Western S+S | Trumpet D4+Tuba D1 | 513 |  | 二胡(Erhu) D4+低音革胡(Bass Gehu) D1 |
| 255 |  | Trumpet D4+French horn D3 | 514 |  | 二胡(Erhu) D4+马头琴(Matouqin) D3 |
| 256 |  | Trumpet D4+Trombone D2 | 515 |  | 高胡(Gaohu) D4+低音革胡(Bass Gehu) D1 |
| 257 |  | French horn D3+Tuba D1 | 516 |  | 高胡(Gaohu) D4+马头琴(Matouqin) D3 |
| 258 |  | French horn D3+Trombone D2 | 517 |  | 高胡(Gaohu) D4+中胡(Zhonghu) D3 |
| 259 |  | Trombone D2+Tuba D1 | 518 |  | 高胡(Gaohu) D4+二胡(Erhu) D4 |

Table 3 the parameters of Speaker

| Parameter | Value |
| --- | --- |
| Frequency response | 35 Hz - 20 kHz (± 2.5 dB) |
| Maximum sound pressure | 124（dB） |
| Long-term sound pressure | 120（dB） |

Table 4 Cronbach alpha of four timbre perception attributes

| variable | Num | Subjects | Cronbach alphas |
| --- | --- | --- | --- |
| fusion | 518 | 32 | 0.932 |
| segregation | 518 | 32 | 0.941 |
| roughness | 518 | 32 | 0.926 |
| pleasantness | 518 | 32 | 0.918 |

Table 5 Frequency distribution statistics of each category of fusion

| **category number** | | **C_1_** | **C_2_** | **C_3_** | **C_4_** | **C_5_** | **C_6_** | **C_7_** | **C_8_** | **C_9_** |
| --- | --- | --- | --- | --- | --- | --- | --- | --- | --- | --- |
| **S+S** | num | 3 | 20 | 17 | 19 | 6 | 30 | 58 | 61 | 12 |
|  | percentage | 1.30% | 8.80% | 7.50% | 8.40% | 2.70% | 13.30% | 25.70% | 27.00% | 5.30% |
| **S+N** | num | 12 | 46 | 78 | 38 | 15 | 21 | 10 | 5 | 2 |
|  | percentage | 5.30% | 20.30% | 34.40% | 16.70% | 6.60% | 9.30% | 4.40% | 2.20% | 0.90% |
| **N+N** | num | 0 | 2 | 11 | 8 | 6 | 9 | 20 | 5 | 4 |
|  | percentage | 0.00% | 3.10% | 16.90% | 12.30% | 9.20% | 13.80% | 30.80% | 7.70% | 6.20% |
| **total** | num | 15 | 68 | 106 | 65 | 27 | 60 | 88 | 71 | 18 |
|  | percentage | 2.90% | 13.10% | 20.50% | 12.50% | 5.20% | 11.60% | 17.00% | 13.70% | 3.50% |

Table 6 One-way ANOVA of fusion (temporal-envelope)

| **instrument types** | **Source** | **Type III Sum of Squares** | **df** | **Mean Square** | **F** | **Sig.** |
| --- | --- | --- | --- | --- | --- | --- |
| **Western instruments** | Corrected Model | 243.343a | 2 | 121.671 | 48.080 | .000 |
|  | Intercept | 5262.950 | 1 | 5262.950 | 2079.731 | .000 |
|  | temporal-envelope | 243.343 | 2 | 121.671 | 48.080 | .000 |
|  | Error | 647.831 | 256 | 2.531 |  |  |
|  | Total | 7196.195 | 259 |  |  |  |
|  | Corrected Total | 891.174 | 258 |  |  |  |
| **Chinese instruments** | Corrected Model | 216.359b | 2 | 108.179 | 44.694 | .000 |
|  | Intercept | 4515.832 | 1 | 4515.832 | 1865.694 | .000 |
|  | temporal-envelope | 216.359 | 2 | 108.179 | 44.694 | .000 |
|  | Error | 619.637 | 256 | 2.420 |  |  |
|  | Total | 7716.618 | 259 |  |  |  |
|  | Corrected Total | 835.996 | 258 |  |  |  |
| a. R Squared = .273 (Adjusted R Squared = .267) | | | | | | |
| b. R Squared = .259 (Adjusted R Squared = .253) | | | | | | |

Table 7 Molecular set of Western instrument fusion evaluation

| **temporal-envelope** | **N** | **Subset 1** | **Subset 2** |
| --- | --- | --- | --- |
| **S+N** | 126 | 3.94 |  |
| **N+N** | 36 |  | 5.67 |
| **S+S** | 97 |  | 5.95 |
| **Sig.** |  | 1.000 | 0.326 |
| Means for groups in homogeneous subsets are displayed.  Based on observed means.  The error term is Mean Square(Error) = 2.531. | | | |
| S-N-K^a,b,c^ | | | |
| a. Uses Harmonic Mean Sample Size = 65.184. | | | |
| b. The group sizes are unequal. The harmonic mean of the group sizes is used. Type I error levels are not guaranteed. | | | |
| c. Alpha = .05. | | | |

Table 8 Molecular set of Chinese instrument fusion evaluation

| **temporal-envelope** | **N** | **Subset 1** | **Subset 2** | **Subset 3** |
| --- | --- | --- | --- | --- |
| **S+N** | 101 | 4.04018755641296 |  |  |
| **N+N** | 29 |  | 5.31294860915812 |  |
| **S+S** | 129 |  |  | 5.99078803653118 |
| **Sig.** |  | 1.000 | 1.000 | 1.000 |
| Means for groups in homogeneous subsets are displayed.  Based on observed means.  The error term is Mean Square(Error) = 2.420. | | | | |
| S-N-K^a,b,c^ | | | | |
| a. Uses Harmonic Mean Sample Size = 57.542. | | | | |
| b. The group sizes are unequal. The harmonic mean of the group sizes is used. Type I error levels are not guaranteed. | | | | |
| c. Alpha = .05. | | | | |

Table 9 One-way ANOVA of fusion (instrument type)

| **temporal-envelope** | **Source** | **Type III Sum of Squares** | **df** | **Mean Square** | **F** | **Sig.** |  |
| --- | --- | --- | --- | --- | --- | --- | --- |
| **S+S** | Corrected Model | .102^a^ | 1 | .102 | .030 | .863 |  |
|  | Intercept | 7891.479 | 1 | 7891.479 | 2285.644 | .000 |  |
|  | instrument types | .102 | 1 | .102 | .030 | .863 |  |
|  | Error | 773.389 | 224 | 3.453 |  |  |  |
|  | Total | 8834.635 | 226 |  |  |  |  |
|  | Corrected Total | 773.491 | 225 |  |  |  |  |
| **S+N** | Corrected Model | .539^b^ | 1 | .539 | .334 | .564 |  |
|  | Intercept | 3572.096 | 1 | 3572.096 | 2210.429 | .000 |  |
|  | instrument types | .539 | 1 | .539 | .334 | .564 |  |
|  | Error | 363.604 | 225 | 1.616 |  |  |  |
|  | Total | 3970.319 | 227 |  |  |  |  |
|  | Corrected Total | 364.143 | 226 |  |  |  |  |
| **N+N** | Corrected Model | 2.088^c^ | 1 | 2.088 | 1.008 | .319 |  |
|  | Intercept | 1938.659 | 1 | 1938.659 | 936.081 | .000 |  |
|  | instrument types | 2.088 | 1 | 2.088 | 1.008 | .319 |  |
|  | Error | 130.475 | 63 | 2.071 |  |  |  |
|  | Total | 2107.858 | 65 |  |  |  |  |
|  | Corrected Total | 132.563 | 64 |  |  |  |  |
| S-N-K^a,b,c^ | | | | | | | |
| a. R Squared = .000 (Adjusted R Squared = -.004) | | | | | | | |
| b. R Squared = .001 (Adjusted R Squared = -.003) | | | | | | |  |
| c. R Squared = .016 (Adjusted R Squared = .000) | | | | | | |  |

Table 10 Two-way ANOVA of fusion

| **Source** | **Type III Sum of Squares** | **df** | **Mean Square** | **F** | **Sig.** |
| --- | --- | --- | --- | --- | --- |
| **Corrected Model** | 465.986a | 5 | 93.197 | 37.647 | .000 |
| **Intercept** | 9731.770 | 1 | 9731.770 | 3931.196 | .000 |
| **instrument types** | .491 | 1 | .491 | .198 | .656 |
| **temporal-envelope** | 451.142 | 2 | 225.571 | 91.120 | .000 |
| **instrument types * temporal-envelope** | 2.695 | 2 | 1.348 | .544 | .581 |
| **Error** | 1267.468 | 512 | 2.476 |  |  |
| **Total** | 14912.812 | 518 |  |  |  |
| **Corrected Total** | 1733.455 | 517 |  |  |  |
| a. R Squared = .269 (Adjusted R Squared = .262) | | | | | |

Table 11 Two-way ANOVA of fusion (main effect)

| **Source** | **Type III Sum of Squares** | **df** | **Mean Square** | **F** | **Sig.** |
| --- | --- | --- | --- | --- | --- |
| **Corrected Model** | 463.291a | 3 | 154.430 | 62.494 | .000 |
| **Intercept** | 9880.371 | 1 | 9880.371 | 3998.312 | .000 |
| **instrument types** | .034 | 1 | .034 | .014 | .906 |
| **temporal-envelope** | 457.006 | 2 | 228.503 | 92.469 | .000 |
| **Error** | 1270.164 | 514 | 2.471 |  |  |
| **Total** | 14912.812 | 518 |  |  |  |
| **Corrected Total** | 1733.455 | 517 |  |  |  |
| a. R Squared = .267 (Adjusted R Squared = .263) | | | | | |

Table 12 Frequency distribution statistics of each category of segregation

| **category number** | | **C_1_** | **C_2_** | **C_3_** | **C_4_** | **C_5_** | **C_6_** | **C_7_** | **C_8_** | **C_9_** |
| --- | --- | --- | --- | --- | --- | --- | --- | --- | --- | --- |
| **S+S** | num | 8 | 45 | 63 | 25 | 14 | 16 | 23 | 25 | 7 |
|  | percentage | 3.5% | 19.9% | 27.9% | 11.1% | 6.2% | 7.1% | 10.2% | 11.1% | 3.1% |
| **S+N** | num | 3 | 4 | 2 | 6 | 6 | 24 | 72 | 85 | 25 |
|  | percentage | 1.3% | 1.8% | 0.9% | 2.6% | 2.6% | 10.6% | 31.7% | 37.4% | 11.0% |
| **N+N** | num | 0 | 6 | 12 | 11 | 4 | 6 | 18 | 7 | 1 |
|  | percentage | 0.0% | 9.2% | 18.5% | 16.9% | 6.2% | 9.2% | 27.7% | 10.8% | 1.5% |
| **total** | num | 11 | 55 | 77 | 42 | 24 | 46 | 113 | 117 | 33 |
|  | percentage | 2.1% | 10.6% | 14.9% | 8.1% | 4.6% | 8.9% | 21.8% | 22.6% | 6.4% |

Table 13 One way ANOVA of segregation (temporal-envelope)

| **instrument types** | **Source** | **Type III Sum of Squares** | **df** | **Mean Square** | **F** | **Sig.** |
| --- | --- | --- | --- | --- | --- | --- |
| **Western instruments** | Corrected Model | 279.472a | 2 | 139.736 | 58.651 | .000 |
|  | Intercept | 5442.168 | 1 | 5442.168 | 2284.206 | .000 |
|  | temporal-envelope | 279.472 | 2 | 139.736 | 58.651 | .000 |
|  | Error | 609.925 | 256 | 2.383 |  |  |
|  | Total | 8793.294 | 259 |  |  |  |
|  | Corrected Total | 889.397 | 258 |  |  |  |
| **Chinese instruments** | Corrected Model | 260.672b | 2 | 130.336 | 54.167 | .000 |
|  | Intercept | 4921.861 | 1 | 4921.861 | 2045.502 | .000 |
|  | temporal-envelope | 260.672 | 2 | 130.336 | 54.167 | .000 |
|  | Error | 615.984 | 256 | 2.406 |  |  |
|  | Total | 8041.912 | 259 |  |  |  |
|  | Corrected Total | 876.656 | 258 |  |  |  |

Table 14 Molecular set of Western instrument segregation evaluation

| **temporal-envelope** | **N** | **Subset 1** | **Subset 2** |
| --- | --- | --- | --- |
| **S+N** | 126 |  | 6.58 |
| **N+N** | 36 | 4.84 |  |
| **S+S** | 97 | 4.40 |  |
| **Sig.** |  | 0.108 | 1.000 |

Table 15 Molecular set of Chinese instrument segregation evaluation

| **temporal-envelope** | **N** | **Subset 1** | **Subset 2** | **Subset 3** |
| --- | --- | --- | --- | --- |
| **S+N** | 101 | 6.47 |  |  |
| **N+N** | 29 |  | 5.23 |  |
| **S+S** | 129 |  |  | 4.32 |
| **Sig.** |  | 1.000 | 1.000 | 1.000 |
| Means for groups in homogeneous subsets are displayed.  Based on observed means.  The error term is Mean Square(Error) = 2.531. | | | | |
| S-N-K^a,b,c^ | | | | |
| a. Uses Harmonic Mean Sample Size = 57.542. | | | | |
| b. The group sizes are unequal. The harmonic mean of the group sizes is used. Type I error levels are not guaranteed. | | | | |
| c. Alpha = .05. | | | | |

Table 16 One-way ANOVA of segregation (instrument type)

| **temporal-envelope** | **Source** | **Type III Sum of Squares** | **df** | **Mean Square** | **F** | **Sig.** |
| --- | --- | --- | --- | --- | --- | --- |
| **S+S** | Corrected Model | .380a | 1 | .380 | .117 | .732 |
|  | Intercept | 4215.546 | 1 | 4215.546 | 1303.162 | .000 |
|  | instrument types | .380 | 1 | .380 | .117 | .732 |
|  | Error | 724.609 | 224 | 3.235 |  |  |
|  | Total | 5015.225 | 226 |  |  |  |
|  | Corrected Total | 724.988 | 225 |  |  |  |
| **S+N** | Corrected Model | .747b | 1 | .747 | .445 | .505 |
|  | Intercept | 9545.046 | 1 | 9545.046 | 5689.042 | .000 |
|  | instrument types | .747 | 1 | .747 | .445 | .505 |
|  | Error | 377.504 | 225 | 1.678 |  |  |
|  | Total | 10059.325 | 227 |  |  |  |
|  | Corrected Total | 378.251 | 226 |  |  |  |
| **N+N** | Corrected Model | 2.455c | 1 | 2.455 | 1.249 | .268 |
|  | Intercept | 1629.041 | 1 | 1629.041 | 829.018 | .000 |
|  | instrument types | 2.455 | 1 | 2.455 | 1.249 | .268 |
|  | Error | 123.797 | 63 | 1.965 |  |  |
|  | Total | 1760.656 | 65 |  |  |  |
|  | Corrected Total | 126.251 | 64 |  |  |  |

Table 17 Two-way ANOVA of segregation

| **Source** | **Type III Sum of Squares** | **df** | **Mean Square** | **F** | **Sig.** |
| --- | --- | --- | --- | --- | --- |
| **Corrected Model** | 549.201a | 5 | 109.840 | 45.875 | .000 |
| **Intercept** | 10331.252 | 1 | 10331.252 | 4314.840 | .000 |
| **instrument types** | .378 | 1 | .378 | .158 | .691 |
| **temporal-envelope** | 530.162 | 2 | 265.081 | 110.711 | .000 |
| **instrument types * temporal-envelope** | 3.402 | 2 | 1.701 | .710 | .492 |
| **Error** | 1225.909 | 512 | 2.394 |  |  |
| **Total** | 16835.206 | 518 |  |  |  |
| **Corrected Total** | 1775.110 | 517 |  |  |  |
| a. R Squared = .309 (Adjusted R Squared = .303) | | | | | |

Table 18 Two-way ANOVA of segregation (main effect)

| **Source** | **Type III Sum of Squares** | **df** | **Mean Square** | **F** | **Sig.** |
| --- | --- | --- | --- | --- | --- |
| **Corrected Model** | 545.799a | 3 | 181.933 | 76.070 | .000 |
| **Intercept** | 10436.165 | 1 | 10436.165 | 4363.573 | .000 |
| **instrument types** | .179 | 1 | .179 | .075 | .785 |
| **temporal-envelope** | 536.742 | 2 | 268.371 | 112.211 | .000 |
| **Error** | 1229.311 | 514 | 2.392 |  |  |
| **Total** | 16835.206 | 518 |  |  |  |
| **Corrected Total** | 1775.110 | 517 |  |  |  |
| a. R Squared = .307 (Adjusted R Squared = .303) | | | | | |

Table 19 Frequency distribution statistics of each category of roughness

| **category number** | | **C_1_** | **C_2_** | **C_3_** | **C_4_** | **C_5_** | **C_6_** | **C_7_** | **C_8_** | **C_9_** |
| --- | --- | --- | --- | --- | --- | --- | --- | --- | --- | --- |
| **S+S** | num | 1 | 24 | 40 | 46 | 23 | 33 | 36 | 21 | 0 |
|  | percentage | 0.4% | 10.7% | 17.8% | 20.4% | 10.2% | 14.7% | 16.0% | 9.3% | 0.0% |
| **S+N** | num | 12 | 46 | 78 | 38 | 15 | 21 | 10 | 5 | 2 |
|  | percentage | 5.3% | 20.3% | 34.4% | 16.7% | 6.6% | 9.3% | 4.4% | 2.2% | 0.9% |
| **N+N** | num | 0 | 2 | 11 | 8 | 6 | 9 | 20 | 5 | 4 |
|  | percentage | 0.0% | 3.1% | 16.9% | 12.3% | 9.2% | 13.8% | 30.8% | 7.7% | 6.2% |
| **total** | num | 2 | 81 | 133 | 103 | 51 | 62 | 49 | 37 | 0 |
|  | percentage | 0.4% | 15.6% | 25.7% | 19.9% | 9.8% | 12.0% | 9.5% | 7.1% | 0.0% |

Table 20 One-way ANOVA of roughness (temporal-envelope)

| **instrument types** | **Source** | **Type III Sum of Squares** | **df** | **Mean Square** | **F** | **Sig.** |
| --- | --- | --- | --- | --- | --- | --- |
| **Western instruments** | Corrected Model | 98.356a | 2 | 49.178 | 15.902 | .000 |
|  | Intercept | 3562.261 | 1 | 3562.261 | 1151.865 | .000 |
|  | temporal-envelope | 98.356 | 2 | 49.178 | 15.902 | .000 |
|  | Error | 791.706 | 256 | 3.093 |  |  |
|  | Total | 6251.156 | 259 |  |  |  |
|  | Corrected Total | 890.062 | 258 |  |  |  |
| **Chinese instruments** | Corrected Model | 26.707b | 2 | 13.353 | 6.833 | .001 |
|  | Intercept | 3312.486 | 1 | 3312.486 | 1695.019 | .000 |
|  | temporal-envelope | 26.707 | 2 | 13.353 | 6.833 | .001 |
|  | Error | 500.287 | 256 | 1.954 |  |  |
|  | Total | 5870.111 | 259 |  |  |  |
|  | Corrected Total | 526.994 | 258 |  |  |  |

Table 21 Molecular set of Western instrument roughness evaluation

| **temporal-envelope** | **N** | **Subset 1** | **Subset 2** | **Subset 3** |
| --- | --- | --- | --- | --- |
| **N+N** | 36 | 3.18 |  |  |
| **S+N** | 126 |  | 4.50 |  |
| **S+S** | 97 |  |  | 5.12 |
| **Sig.** |  | 1.000 | 1.000 | 1.000 |
| Alpha = 0.05。 | | | | |

Table 22 Molecular set of Chinese instrument roughness evaluation

| **temporal-envelope** | **N** | **Subset 1** | **Subset 2** |
| --- | --- | --- | --- |
| **N+N** | 29 | 3.98 |  |
| **S+N** | 101 | 4.31 |  |
| **S+S** | 129 |  | 4.85 |
| **Sig.** |  | .209 | 1.000 |
| Means for groups in homogeneous subsets are displayed.  Based on observed means.  The error term is Mean Square(Error) = 2.420. | | | |
| S-N-K^a,b,c^ | | | |
| a. Uses Harmonic Mean Sample Size = 57.542. | | | |
| b. The group sizes are unequal. The harmonic mean of the group sizes is used. Type I error levels are not guaranteed. | | | |
| c. Alpha = .05. | | | |

Table 23 One-way ANOVA of roughness (instrument type)

| **temporal-envelope** | **Source** | **Type III Sum of Squares** | **df** | **Mean Square** | **F** | **Sig.** |
| --- | --- | --- | --- | --- | --- | --- |
| **S+S** | Corrected Model | 3.916a | 1 | 3.916 | 1.335 | .249 |
|  | Intercept | 5497.614 | 1 | 5497.614 | 1875.004 | .000 |
|  | instrument types | 3.916 | 1 | 3.916 | 1.335 | .249 |
|  | Error | 656.780 | 224 | 2.932 |  |  |
|  | Total | 6228.465 | 226 |  |  |  |
|  | Corrected Total | 660.696 | 225 |  |  |  |
| **S+N** | Corrected Model | 2.106b | 1 | 2.106 | .809 | .369 |
|  | Intercept | 4355.675 | 1 | 4355.675 | 1672.965 | .000 |
|  | instrument types | 2.106 | 1 | 2.106 | .809 | .369 |
|  | Error | 585.802 | 225 | 2.604 |  |  |
|  | Total | 5018.446 | 227 |  |  |  |
|  | Corrected Total | 587.909 | 226 |  |  |  |
| **N+N** | Corrected Model | 10.204c | 1 | 10.204 | 13.010 | .001 |
|  | Intercept | 824.934 | 1 | 824.934 | 1051.811 | .000 |
|  | instrument types | 10.204 | 1 | 10.204 | 13.010 | .001 |
|  | Error | 49.411 | 63 | .784 |  |  |
|  | Total | 874.356 | 65 |  |  |  |
|  | Corrected Total | 59.614 | 64 |  |  |  |

Table 24 Two-way ANOVA of roughness

| **Source** | **Type III Sum of Squares** | **df** | **Mean Square** | **F** | **Sig.** |
| --- | --- | --- | --- | --- | --- |
| **Corrected Model** | 125.070a | 5 | 25.014 | 9.913 | .000 |
| **Intercept** | 6858.036 | 1 | 6858.036 | 2717.749 | .000 |
| **instrument types** | 1.159 | 1 | 1.159 | .459 | .498 |
| **temporal-envelope** | 105.992 | 2 | 52.996 | 21.002 | .000 |
| **instrument types * temporal-envelope** | 14.943 | 2 | 7.471 | 2.961 | .053 |
| **Error** | 1291.994 | 512 | 2.523 |  |  |
| **Total** | 12121.267 | 518 |  |  |  |
| **Corrected Total** | 1417.064 | 517 |  |  |  |
| a. R Squared = . 088 (Adjusted R Squared = . 079) | | | | | |

Table 25 Two-way ANOVA of roughness (main effect)

| **Source** | **Type III Sum of Squares** | **df** | **Mean Square** | **F** | **Sig.** |
| --- | --- | --- | --- | --- | --- |
| **Corrected Model** | 110.127a | 3 | 36.709 | 14.437 | .000 |
| **Intercept** | 6888.183 | 1 | 6888.183 | 2709.027 | .000 |
| **instrument types** | 1.283 | 1 | 1.283 | .505 | .478 |
| **temporal-envelope** | 110.120 | 2 | 55.060 | 21.654 | .000 |
| **Error** | 1306.936 | 514 | 2.543 |  |  |
| **Total** | 12121.267 | 518 |  |  |  |
| **Corrected Total** | 1417.064 | 517 |  |  |  |
| a. R Squared = .078 (Adjusted R Squared = .072) | | | | | |

Table 26 Frequency distribution statistics of each category of pleasantness

| **category number** | | **C_1_** | **C_2_** | **C_3_** | **C_4_** | **C_5_** | **C_6_** | **C_7_** | **C_8_** | **C_9_** |
| --- | --- | --- | --- | --- | --- | --- | --- | --- | --- | --- |
| **S+S** | num | 0 | 23 | 38 | 42 | 49 | 44 | 26 | 4 | 0 |
|  | percentage | 0.00% | 10.22% | 16.89% | 18.67% | 21.78% | 19.56% | 11.56% | 1.78% | 0.00% |
| **S+N** | num | 0 | 14 | 23 | 22 | 43 | 68 | 45 | 12 | 0 |
|  | percentage | 0.00% | 6.19% | 10.18% | 9.73% | 19.03% | 30.09% | 19.91% | 5.31% | 0.00% |
| **N+N** | num | 0 | 0 | 0 | 2 | 8 | 21 | 21 | 13 | 0 |
|  | percentage | 0.00% | 0.00% | 0.00% | 3.13% | 12.50% | 32.81% | 32.81% | 20.31% | 0.00% |
| **total** | num | 0 | 37 | 61 | 66 | 100 | 133 | 92 | 29 | 0 |
|  | percentage | 0.00% | 7.14% | 11.78% | 12.74% | 19.31% | 25.68% | 17.76% | 5.60% | 0.00% |

Table 27 One-way ANOVA of pleasantness (temporal-envelope)

| **instrument types** | **Source** | **Type III Sum of Squares** | **df** | **Mean Square** | **F** | **Sig.** |
| --- | --- | --- | --- | --- | --- | --- |
| **Western instruments** | Corrected Model | 93.578a | 2 | 46.789 | 17.417 | .000 |
|  | Intercept | 5868.551 | 1 | 5868.551 | 2184.596 | .000 |
|  | temporal-envelope | 93.578 | 2 | 46.789 | 17.417 | .000 |
|  | Error | 687.701 | 256 | 2.686 |  |  |
|  | Total | 7792.800 | 259 |  |  |  |
|  | Corrected Total | 781.279 | 258 |  |  |  |
| **Chinese instruments** | Corrected Model | 63.168b | 2 | 31.584 | 18.807 | .000 |
|  | Intercept | 4952.007 | 1 | 4952.007 | 2948.744 | .000 |
|  | temporal-envelope | 63.168 | 2 | 31.584 | 18.807 | .000 |
|  | Error | 429.917 | 256 | 1.679 |  |  |
|  | Total | 7108.369 | 259 |  |  |  |
|  | Corrected Total | 493.085 | 258 |  |  |  |

Table 28 Molecular set of Western instrument pleasantness evaluation

| **temporal-envelope** | **N** | **Subset 1** | **Subset 2** | **Subset 3** |
| --- | --- | --- | --- | --- |
| **S+S** | 97 | 4.65 |  |  |
| **S+N** | 126 |  | 5.25 |  |
| **N+N** | 36 |  |  | 6.54 |
| **Sig.** |  | 1.000 | 1.000 | 1.000 |
| Alpha = 0.05。 | | | | |

Table 29 Molecular set of Chinese instrument pleasantness evaluation

| **temporal-envelope** | **N** | **Subset 1** | **Subset 2** | **Subset 3** |
| --- | --- | --- | --- | --- |
| **S+S** | 129 | 4.63 |  |  |
| **S+N** | 101 |  | 5.27 |  |
| **N+N** | 29 |  |  | 6.16 |
| **Sig.** |  | 1.000 | 1.000 | 1.000 |
| Means for groups in homogeneous subsets are displayed.  Based on observed means.  The error term is Mean Square(Error) = 2.420. | | | | |
| S-N-K^a,b,c^ | | | | |
| a. Uses Harmonic Mean Sample Size = 57.542. | | | | |
| b. The group sizes are unequal. The harmonic mean of the group sizes is used. Type I error levels are not guaranteed. | | | | |
| c. Alpha = .05. | | | | |

Table 30 One-way ANOVA of pleasantness (instrument type)

| **temporal-envelope** | **Source** | **Type III Sum of Squares** | **df** | **Mean Square** | **F** | **Sig.** |
| --- | --- | --- | --- | --- | --- | --- |
| **S+S** | Corrected Model | .019a | 1 | .019 | .009 | .926 |
|  | Intercept | 4774.785 | 1 | 4774.785 | 2129.968 | .000 |
|  | instrument types | .019 | 1 | .019 | .009 | .926 |
|  | Error | 502.145 | 224 | 2.242 |  |  |
|  | Total | 5371.852 | 226 |  |  |  |
|  | Corrected Total | 502.164 | 225 |  |  |  |
| **S+N** | Corrected Model | .039b | 1 | .039 | .016 | .900 |
|  | Intercept | 6202.184 | 1 | 6202.184 | 2539.528 | .000 |
|  | instrument types | .039 | 1 | .039 | .016 | .900 |
|  | Error | 549.508 | 225 | 2.442 |  |  |
|  | Total | 6824.430 | 227 |  |  |  |
|  | Corrected Total | 549.547 | 226 |  |  |  |
| **N+N** | Corrected Model | 2.248c | 1 | 2.248 | 2.147 | .148 |
|  | Intercept | 2589.635 | 1 | 2589.635 | 2473.239 | .000 |
|  | instrument types | 2.248 | 1 | 2.248 | 2.147 | .148 |
|  | Error | 65.965 | 63 | 1.047 |  |  |
|  | Total | 2704.887 | 65 |  |  |  |
|  | Corrected Total | 68.213 | 64 |  |  |  |

Table 31 Two-way ANOVA of pleasantness

| **Source** | **Type III Sum of Squares** | **df** | **Mean Square** | **F** | **Sig.** |
| --- | --- | --- | --- | --- | --- |
| **Corrected Model** | 159.627a | 5 | 31.925 | 14.626 | .000 |
| **Intercept** | 10762.119 | 1 | 10762.119 | 4930.313 | .000 |
| **temporal-envelope** | 151.656 | 2 | 75.828 | 34.738 | .000 |
| **instrument types** | 1.369 | 1 | 1.369 | .627 | .429 |
| **instrument types * temporal-envelope** | 2.062 | 2 | 1.031 | .472 | .624 |
| **Error** | 1117.618 | 512 | 2.183 |  |  |
| **Total** | 14901.168 | 518 |  |  |  |
| **Corrected Total** | 1277.245 | 517 |  |  |  |

Table 32 Two-way ANOVA of pleasantness (main effect)

| **Source** | **Type III Sum of Squares** | **df** | **Mean Square** | **F** | **Sig.** |
| --- | --- | --- | --- | --- | --- |
| **Corrected Model** | 157.565a | 3 | 52.522 | 24.111 | .000 |
| **Intercept** | 10922.575 | 1 | 10922.575 | 5014.114 | .000 |
| **temporal-envelope** | 154.684 | 2 | 77.342 | 35.505 | .000 |
| **instrument types** | .244 | 1 | .244 | .112 | .738 |
| **Error** | 1119.680 | 514 | 2.178 |  |  |
| **Total** | 14901.168 | 518 |  |  |  |
| **Corrected Total** | 1277.245 | 517 |  |  |  |
| a. R Squared = .123 (Adjusted R Squared = .118) | | | | | |


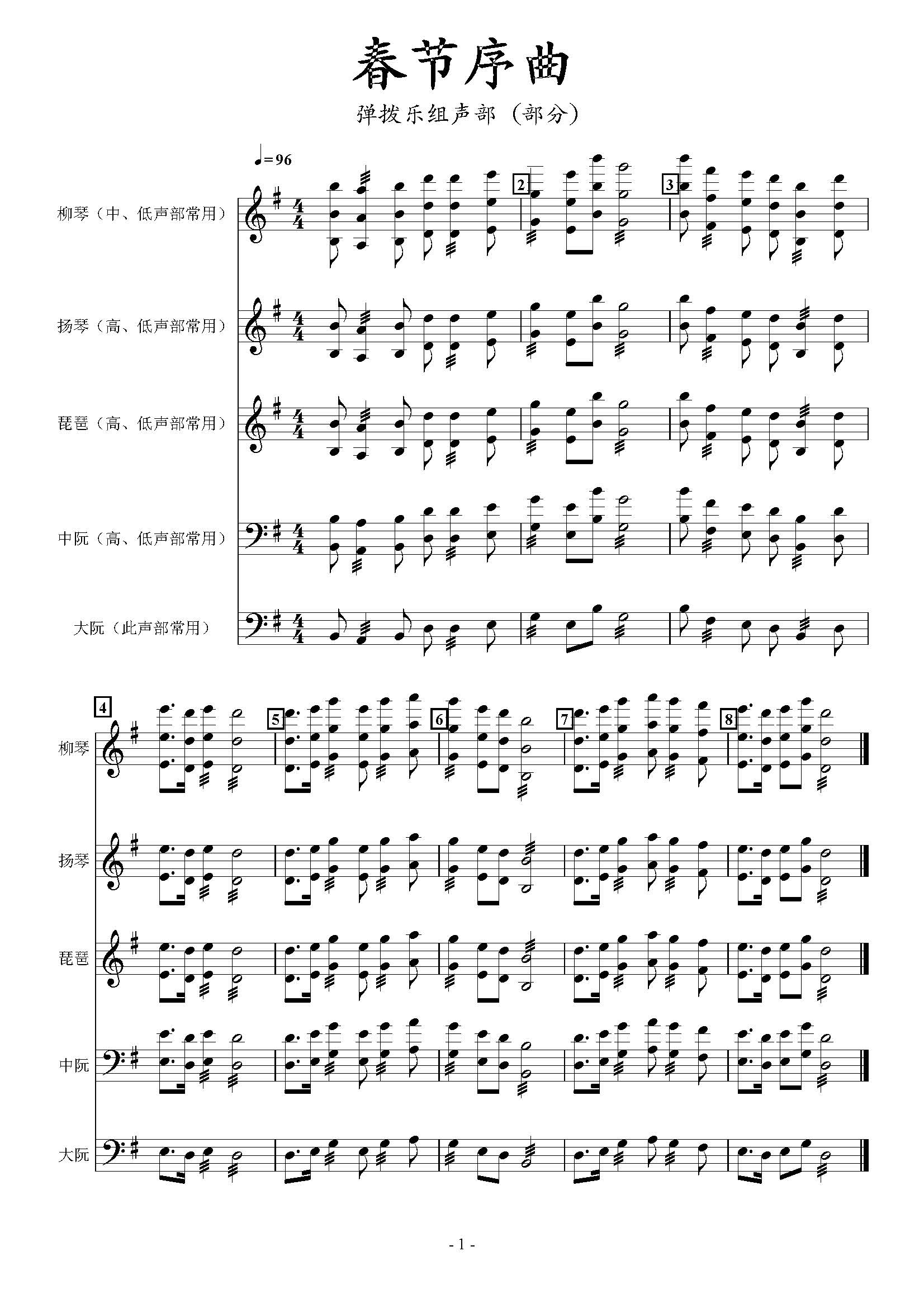


Figure 1 The music score of Spring Festival prelude

Figure 2 Normal P-P distribution of fusion

Figure 3 Normal P-P distribution of fusion

Figure 4 Normal P-P distribution of segregation

Figure 5 Normal P-P distribution of segregation

Figure 6 Normal P-P distribution of roughness

Figure 7 Normal P-P distribution of roughness

Figure 8 Normal P-P distribution of pleasantness

Figure 9 Normal P-P distribution of pleasantness
